# Scalp‐negative medial temporal interictal epileptic discharges alter large‐scale brain networks: A simultaneous high‐density electroencephalographic and intracranial electroencephalographic study

**DOI:** 10.1002/epi.70061

**Published:** 2025-12-22

**Authors:** Nicolas Roehri, Pia De Stefano, Laurent Spinelli, Renaud Marquis, Stanislas Lagarde, Shahan Momjian, Margitta Seeck, Serge Vulliemoz

**Affiliations:** ^1^ EEG and Epilepsy Unit, University Hospitals and Faculty of Medicine University of Geneva Geneva Switzerland; ^2^ Neuro‐Intensive Care Unit, Department of Intensive Care University Hospitals of Geneva Geneva Switzerland; ^3^ Institut de Neurosciences Des Systèmes, Aix‐Marseille Université, INSERM, INS Marseille France; ^4^ Neurosurgery Department, Geneva University Hospitals and Faculty of Medicine University of Geneva Geneva Switzerland; ^5^ Center for Biomedical Imaging Lausanne Switzerland

**Keywords:** epilepsy, interictal epileptiform discharge, simultaneous intracranial and high‐density EEG

## Abstract

**Objective:**

Interictal epileptiform discharges (IEDs) observed on scalp electroencephalography (EEG) serve as a diagnostic hallmark of epilepsy. However, only a small fraction of IEDs recorded by intracranial EEG (iEEG) are detectable on the scalp; the vast majority remain invisible on scalp recordings. Nevertheless, epilepsy is associated with important brain network alterations and cognitive symptoms between seizures. Our study aims to investigate the impact of these scalp‐negative IEDs on the whole‐brain network, particularly those produced in medial temporal regions, given their consistent involvement across patients, their potential implications in cognitive deficit, and their relevance in both epilepsy and Alzheimer disease. To achieve this, we leveraged simultaneous iEEG and high‐density EEG (hdEEG) recordings.

**Methods:**

We analyzed IEDs occurring solely in the medial temporal regions, not visible on the scalp, in nine patients with drug‐resistant epilepsy during simultaneous iEEG‐hdEEG recordings. These scalp‐negative IEDs were compared to epochs without any epileptic activity recorded in either modality. Spectral and network characteristics were investigated at the region of interest (ROI) for both modalities in different canonical frequency bands.

**Results:**

ROI time courses reconstructed with hdEEG during IEDs exhibited sharp wave characteristics (cluster‐based corrected time–frequency analysis), confirming that some information time‐locked to iEEG IEDs reached the scalp. Our findings revealed a significant frequency‐dependent, large‐scale increase in power and network features during scalp‐negative IEDs compared to epochs without IEDs, at the whole‐brain level and within specific ROIs, including the ipsilateral hippocampus (*p* < .05, false discovery rate corrected).

**Significance:**

Despite being localized in medial temporal regions, these IEDs have a widespread impact on functional brain networks, potentially contributing to cognitive impairment in epilepsy. This study lays the groundwork for examining their real‐time effects on cognition, including in neurodegenerative conditions such as Alzheimer disease, where these IEDs can be present and difficult to detect.

**Key points**

- Simultaneous iEEG and hdEEG revealed time‐locked scalp activity during scalp‐negative IEDs.
- Automated detection confirmed that the selected IEDs originated from the medial temporal lobe only.
- Scalp‐negative IEDs increased power and altered brain networks, as shown by iEEG‐ and hdEEG‐derived ROI signals.
- Although limited to medial temporal regions, these scalp‐negative IEDs impacted large‐scale brain networks.
- This large‐scale and frequency‐dependent network alteration could contribute to cognitive impairment.

## 1 INTRODUCTION

Interictal epileptic discharges (IEDs) on scalp electroencephalography (EEG) are a crucial diagnostic feature of epilepsy. However, in many patients, scalp IEDs occur infrequently or may even be entirely absent, which can lead to delays in diagnosis despite the severity of the disease.^1^, ^2^, ^3^ A small fraction of IEDs recorded by intracranial EEG (iEEG), approximately 14%, are apparent on the scalp; in other words, the majority of iEEG IEDs are not visible on routine scalp EEG.^4^ Similar to how magnetic resonance imaging (MRI) scans that do not reveal lesions are referred to as “negative MRIs,” we use the term scalp‐negative IEDs to describe IEDs not visible on scalp EEG, despite being clearly detectable in intracranial recordings. These scalp‐negative IEDs may arise from several factors, such as the volume, depth, and orientation of the cortical sources of IEDs, the level of synchronization of neuronal populations, and the signal‐to‐noise ratio (SNR), notably compared to concomitant activity of superficial regions.^4^, ^5^, ^6^, ^7^, ^8^

Additionally, alterations of physiological brain networks occur even during periods when no IEDs are detectable on scalp EEG. This has been demonstrated through high‐density EEG (hdEEG) studies^9^, ^10^, ^11^ and by simultaneous EEG and functional MRI (EEG‐fMRI) studies.^12^ In some cases, these changes may be driven by scalp‐negative IEDs. Connectivity patterns among regions implicated in epileptic activity remain largely consistent whether IEDs are present or absent on the scalp.^13^ iEEG studies showed differences in connectivity between the epileptogenic zone and the rest of the brain,^14^ even in the absence of IEDs.^15^, ^16^ The occurrence of IEDs further alters connectivity patterns, as demonstrated by IED‐related network changes observed using EEG^17^, ^18^ or EEG‐fMRI.^13^ The role of interictal epileptic activity in (dys)cognitive manifestations represents an open issue in a range of brain disorders, including neurodegenerative (e.g., dementia) conditions. It has been demonstrated that IEDs impact cognitive performance.^19^, ^20^ Moreover, medial temporal scalp‐negative IEDs in Alzheimer disease have been hypothesized to contribute to cognitive deficits,^21^ possibly via transient large‐scale network disruptions. Understanding the impact of subtle medial temporal IEDs on whole‐brain networks is thus a central research question.

Simultaneous hdEEG‐iEEG recordings in patients with drug‐resistant epilepsy offer a rare opportunity to link focal epileptic activity with whole‐brain network dynamics.^22^ Although iEEG provides precise detection of spatially restricted IEDs, including scalp‐negative IEDs, its limited spatial coverage hinders comprehensive whole‐brain network analysis. In contrast, hdEEG allows for whole‐brain network estimation but lacks direct access to deep epileptogenic sources. Combining them allows confident identification of scalp‐negative IEDs and assessment of their whole‐brain network impact.

In this study, we focused our analysis on medial temporal scalp‐negative IEDs for their consistent occurrence across patients and for their relevance to both epilepsy and Alzheimer disease. We hypothesized that even limited medial temporal IEDs can impact large‐scale brain networks. These analyses were conducted at both reconstructed source and iEEG levels, the latter serving as the best available gold standard despite the sampling limitations mentioned above. Such network alteration could have significant implications by helping to (1) confirm large‐scale network effect of focal pathological activity, potentially interacting with physiological/cognitive networks; and (2) design new scalp markers of epileptic activity.

## 2 MATERIALS AND METHODS

### 2.1 Study setting

At the EEG and Epilepsy Unit of Geneva University Hospital, we conducted simultaneous recordings of iEEG and hdEEG as part of a research project during presurgical evaluation of patients with pharmacoresistant focal epilepsy. All simultaneous recordings were performed on the last day before removing the iEEG electrodes and lasted between 30 min and a couple of hours. We studied all consecutive patients between 2014 and 2019 who agreed to take part in this simultaneous recording procedure. We collected neuropsychological scores from the routine clinical presurgical evaluation, specifically the delayed memory recall scores of the Rey Auditory Verbal Learning Test (15 words) and the Rey Visual Design Learning Test (15 figures), assessing verbal and nonverbal episodic memory, respectively. This study was approved by the Regional Research Ethics Committee (13‐004).

### 2.2 Anatomical parcellation and electrode localization

For each patient, we used a structural T1 magnetic resonance image, recorded on a 3‐T scanner (Siemens Prisma) before electrode implantation and a computed tomography (CT) scan after implantation, obtained as part of our clinical procedure for iEEG recordings.

Using Connectome Mapper 3 software,^23^, ^24^ we resampled each MRI to 1‐mm^3^ isotropic resolution using cubic interpolation and performed cortical and subcortical brain parcellation based on the Desikan–Killiany anatomical atlas, removing basal ganglia, thalamus, brainstem, and cerebellum. This resulted in 72 regions of interest (ROIs) accounting for cortical gray matter structures, including hippocampus and amygdala. To localize the iEEG electrodes, we used Cartool^25^ software to manually extract their positions from the CT scan. We then realigned the electrode positions into the patient's MRI space using linear coregistration between the CT and the preimplantation MRI and used the individual atlas to assign one ROI to each iEEG contact. Finally, the individual MRIs were nonlinearly coregistered to Montreal Neurological Institute (MNI) space solely to display all iEEG shafts into the same space using Fieldtrip.^26^

### 2.3 Simultaneous EEG and iEEG acquisition

The simultaneous recording procedure and the steps to minimize infectious risk are detailed in De Stefano et al.^27^ In short, we performed the simultaneous scalp and iEEG recording on the last day before removing the iEEG electrodes. iEEG recordings were performed using intracerebral macroelectrodes (Table S1). We used a geodesic elastic net of 257 plastic cup scalp electrodes (Electrical Geodesic Inc.) with conductive paste. We recorded hdEEG at a sampling rate of 1000 Hz and iEEG at a sampling rate of 2048 Hz.

A board‐certified EEG expert (P.D.S. or S.V.) visually identified and marked IEDs on iEEG. For each patient, we first selected only the most frequent medial temporal IEDs that (1) did not involve the electrode contacts of the lateral temporal cortex, (2) were not visible on scalp EEG, and (3) occurred in the most prevalent arousal state during the individual recording several hours away from clinical seizures. If an IED was labeled as not visible on the scalp EEG, although there may have been a concomitant change in the hdEEG traces, this change did not meet the criteria to be identified as an IED.^28^ These IEDs will be referred to as scalp‐negative IEDs. As a control condition, we selected epochs without any epileptic activity in both modalities that occurred during the same arousal state as the selected scalp‐negative IEDs, referred to as no‐IED epoch. To address sample size bias, we randomly selected an equal number of epochs of the two conditions within each patient. In addition, we employed an automated IED detector (Delphos^29^, ^30^) to quantify both the number and spatial extent of IEDs in close temporal proximity to the identified scalp‐negative IEDs. We report the normalized rates of IEDs, calculated as the rate divided by the maximum rate for each patient. This independent IED detection allows us to validate that the selected scalp‐negative IEDs predominantly involve medial temporal structures. The automated detection further allowed us to quantify the extent of the irritative zone (IZ). An ROI was considered part of the IZ if its average IED rate exceeded 1/10 of the maximum rate observed across all ROIs (Tables S1 and S2).

### 2.4 EEG and iEEG preprocessing

The hdEEG and iEEG recordings were downsampled to 200 Hz and filtered in the frequency band of 1–35 Hz using a zero‐phase distortion fourth‐order Butterworth filter. Independent component analysis was applied to hdEEG to remove oculomotor, cardiac, and muscle artifacts following the recommendation of Jung et al.^31^ Bad electrodes were interpolated using a three‐dimensional spherical spline.^32^ Finally, the hdEEG signals were rereferenced to the common average reference and the iEEG traces were rereferenced in a bipolar manner, which involved subtracting the signal recorded by one channel from the signal recorded by an adjacent channel of the same shaft. Pairs of channels were kept for analysis if at least one of the two channels were located in the gray matter.

### 2.5 Electrical source imaging

Approximately 5000 sources with unconstrained orientation were equally distributed within the gray matter volume. The EEG forward model was computed with the boundary element method for a three‐layer model using OpenMEEG^33^ and default conductivity values based on individual MRI. To compute the inverse operator, we prewhitened the leadfield with the covariance matrix estimated from the no‐IED epochs and applied exact low‐resolution brain electromagnetic tomography^34^ using the FieldTrip toolbox^26^ (regularization parameter set to 3). We considered the first singular vector as the representative time series of each ROI.^35^ These time series are referred to as hdEEG(ROI) signals. For the sake of comparison across patients, we swapped the hemispheres for patients with selected scalp‐negative IEDs originating from the left hemisphere (Table S1). We will therefore refer to the ipsilateral hemisphere as the hemisphere where the iEEG IEDs were marked and the contralateral hemisphere otherwise. To characterize the reconstructed signal, we first computed time‐locked and time–frequency analyses using the ipsilateral hippocampal ROI and the marked temporomedial iEEG signal. Subsequently, we computed the evoked and induced changes for each ROI.

### 2.6 Time‐locked and time–frequency analysis

The analysis window for scalp‐negative IEDs spanned −2 to 3 s, aligned to the iEEG IED peak (0 s). For the no‐IED epochs, a ±2.5‐s window was used, centered on the marker. For the time–frequency analysis, we used a Morse wavelet (symmetry = 3, time bandwidth = 40, 10 voices/octave, frequency limits = 1–40 Hz). We computed the cone of influence (COI) of the wavelet to estimate the minimum temporal resolution per frequency. For each patient and epoch type, we computed the event‐related spectral perturbation (ERSP) in decibesl^36^ and intertrial (phase) coherence (ITC).^37^ ERSP quantifies both evoked and induced components of the response (i.e., whether phase‐locked or not), whereas the ITC only quantifies the evoked (i.e., phase‐locked) part of the response. Cluster‐based permutation tests (512 permutations, i.e., 2^9^ for nine patients) assessed changes using one‐sided paired *t*‐tests.^26^, ^38^ Cluster‐forming thresholds were set at *p* = .0005 (ERSP) and *p* = .05 (ITC) with significance of the summed *t*‐value within clusters set at *p* < .001 (ERSP) and *p* < .05 (ITC).

### 2.7 Adaptive analyzing windows

**FIGURE 1:**
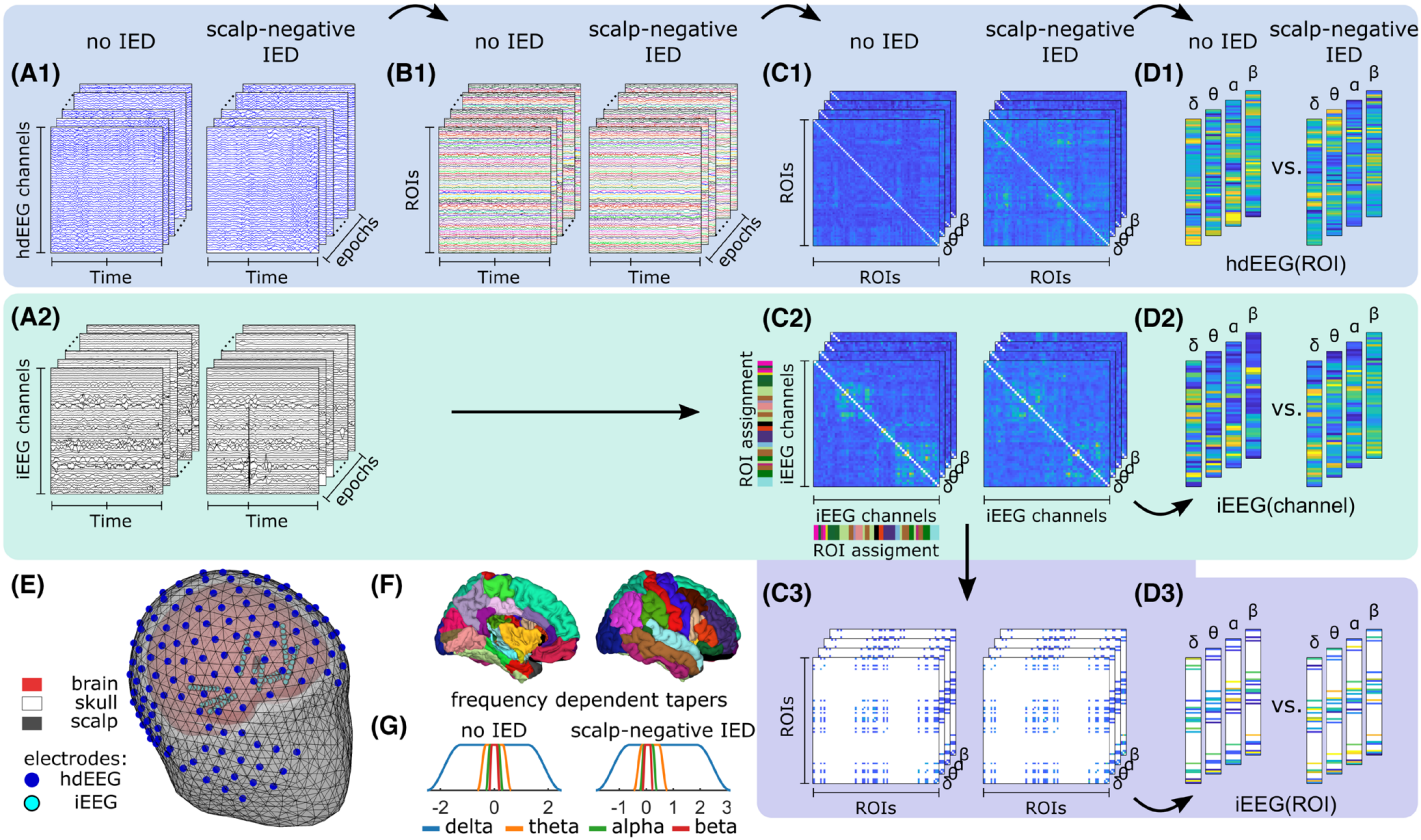
Preprocessing and analyzing pipeline. Epochs of high‐density electroencephalography (hdEEG; A.1) and intracranial EEG (iEEG; A.2) were defined as periods exhibiting medial temporal interictal epileptic discharges (IEDs) visible only on the iEEG (scalp‐negative IEDs) as well as periods characterized by the absence of IEDs on both iEEG and hdEEG (no IED). Individual head models were generated to calculate the leadfield and the inverse operator (E). Regions of interest (ROIs) from the Desikan–Killiany atlas (F) were used to derive ROI‐time courses based on projected hdEEG (B.1). Connectivity analysis was applied to ROI (C.1) and iEEG epochs (C.2) using frequency‐dependent tapers (G). To compare the results of the iEEG with those of the ROI, the connectivity matrix of the iEEG was summarized at the ROI level, including only the regions sampled by the implanted electrodes, referred to as iEEG(ROI) (C.3). ROIs or ROI pairs not covered by iEEG are shown in white. Network features were extracted from each of the three types of connectivity matrices to obtain ROI‐level (D.1–D.3) and channel‐level (D.2) network features. Averaging these features across ROIs or channels yielded the whole‐brain network feature.

In this study, we are interested in the changes induced by scalp‐negative IEDs. We therefore decided to shift in time the analyzing window to include more samples after the IED than before. The windows are positioned so that the peak of the IED occurs at 3/8 of the window length. Moreover, to be able to study different frequency bands and preserve temporal specificity, we adapted the length of the window to the frequency band under investigation and set it to accommodate five cycles of the lowest frequency in the band of interest (e.g., 5 s for the delta frequency band [1–4 Hz]). Finally, we chose a Tukey window rather than the Hann window to preserve the peak of the IEDs (exemplified in Figure 1G). These window settings are used in the subsequent analyses.

### 2.8 Spectrum analysis

For each ROI, we calculated the power spectral density (PSD), quantifying evoked and induced activity, and ITC, measuring evoked activity, of the two types of epochs and two recordings. The PSD and ITC were averaged across trials and further averaged within four frequency bands (delta: [1–4 Hz], theta: [4–8 Hz], alpha: [8–13 Hz], beta: [13–30 Hz]). This resulted in one value of power or ITC per ROI or iEEG electrode, frequency band, and epoch type. To allow for a direct comparison between the results obtained from hdEEG and iEEG, we averaged the power/ITC of the iEEG channels sampling the same ROI. This measure will be referred to as iEEG(ROI) power/ITC.

### 2.9 Connectivity analysis

Functional connectivity was assessed using the weighted phase lag index (wPLI),^39^ a measure that determines the level of phase synchrony between any given pair of ROIs, is robust to spatial leakage, and is less biased by the amount of signal mixing. For both the reconstructed hdEEG(ROI) and the iEEG signals, wPLI was computed and averaged within each frequency band, as for the spectral analysis (Figure 1C). Again, to allow for a direct comparison between hdEEG and iEEG, we calculated ROI‐ROI connectivity based on the connectivity values of the iEEG and their ROI label. Specifically, we averaged connectivity measures of iEEG channels belonging to each pair of ROIs to obtain new values referred to as iEEG(ROI) connectivity (Figure 1C.3).

### 2.10 Network analysis

Brain network topology was assessed at the whole brain network, ROI, and iEEG channel levels, with a focus on two topological features: segregation and integration (Figure 1D).

Segregation pertains to the capacity of specialized processing to occur within densely interconnected groups of regions or modules. The clustering coefficient^40^ was used as a measure of ROI/channel segregation. This parameter quantifies the prevalence of densely interconnected regions around individual regions. The average clustering coefficient provided an assessment of the network's level of segregation.

Integration quantifies the network's ability to rapidly combine information from distributed brain regions.^41^ Integration was evaluated using nodal efficiency (NE), which measures the average of the inverse shortest path length connecting two regions.^41^ The global efficiency, its counterpart at the network level, was obtained by averaging NE over all ROIs/channels. These measures were computed for each frequency band and each patient using the weighted connectivity matrices (i.e., unthresholded matrices).

### 2.11 Statistical analysis for spectral and network measures

A one‐sided Wilcoxon signed‐rank test was applied to the network‐level measures from hdEEG(ROI), iEEG(channel), and iEEG(ROI) data to compare integration and segregation between no‐IED and scalp‐negative IED epochs. We used a one‐sided test due to the relatively small cohort and our primary expectation of an increase in these measures. Multiple comparisons across four frequency bands were controlled using a false discovery rate (FDR) procedure.^42^

The same test assessed ROI‐level increases in power, ITC, segregation, or integration between no‐IED and scalp‐negative IED epochs for both hdEEG(ROI) and iEEG(ROI) values. For iEEG(ROI), we only tested the ROIs that were sampled in at least five patients. FDR correction accounted for the number of ROIs and frequency bands, corresponding to 72 × 4 and 12 × 4 comparisons for hdEEG(ROI) and iEEG(ROI), respectively.

Separate generalized linear mixed‐effects models were used to evaluate the effects of IZ size and neuropsychological test scores on whole‐brain network integration and segregation. Both models used a gamma distribution with logit function and included frequency band and its interaction as covariates, with a random intercept per patient.

## 3 RESULTS

### 3.1 Patient characteristics and number of IEDs

**FIGURE 2:**
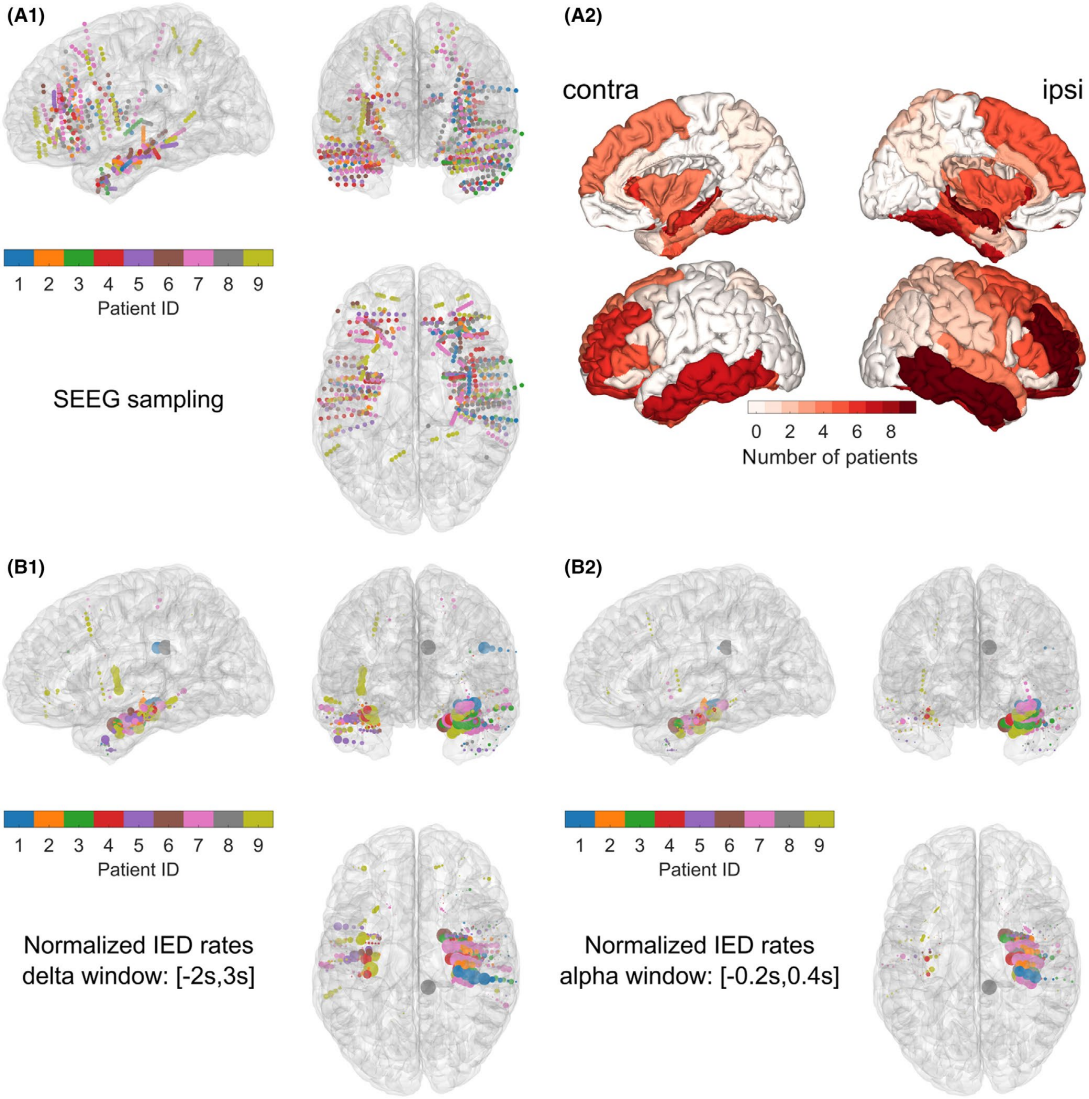
Intracranial electroencephalographic (iEEG) channel localization and normalized interictal epileptic discharge (IED) rates for all patients in the Montreal Neurological Institute (MNI) space. The dots correspond to the midpoint between the two contacts of bipolar channels. For the sake of comparison across patients, the hemispheres of patients with selected scalp‐negative IEDs originating from the left hemisphere were swapped. (A.1) iEEG channels are depicted in the MNI space as dots colored according to the patients. (A.2) Regions of interest (ROIs) of the atlas are color‐coded based on the number of patients for whom at least one iEEG channel samples a given ROI. (B) The normalized IED rates of each patient are represented by dots of different sizes (maximum size corresponds to 1) placed at the location of the iEEG electrodes. These rates are computed on the window used either for the delta band (B.1) or alpha band (B.2) around the scalp‐negative IEDs. Around the selected IEDs, the IED rates are higher in the temporal medial structures. SEEG, stereo‐EEG.

From 10 recorded patients, we analyzed nine patients (median age = 40 years, range = 21–53 years, three females, one during non‐rapid eye movement sleep; Table S1). One recording was excluded due to insufficient data quality. Figure 2A shows the location of the iEEG bipolar channels in MNI space as the midpoint between the adjacent bipolar contacts of each patient. The number of patients for each ROI sampled by iEEG is depicted in Figure 2B. The 12 regions implanted in more than five patients, which were retained for the iEEG(ROI) analysis, included bilaterally the lateral orbitofrontal gyri, rostral middle frontal gyri, inferior and middle temporal gyri, and hippocampus, as well as the ipsilateral fusiform gyrus and ipsilateral amygdala. The normalized rates of IEDs as quantified by Delphos are shown at the location of the iEEG channels around the marked scalp‐negative IEDs (Figure 2B). This analysis confirmed that although some IEDs were observed in non‐medial temporal structures around the selected scalp‐negative IEDs, the IED rates were largely higher in the medial temporal structures, particularly within a shorter time window. This finding confirms that the manual identification of scalp‐negative IEDs accurately targeted paroxysms confined to the medial temporal regions.

### 3.2 Spectral changes

**FIGURE 3:**
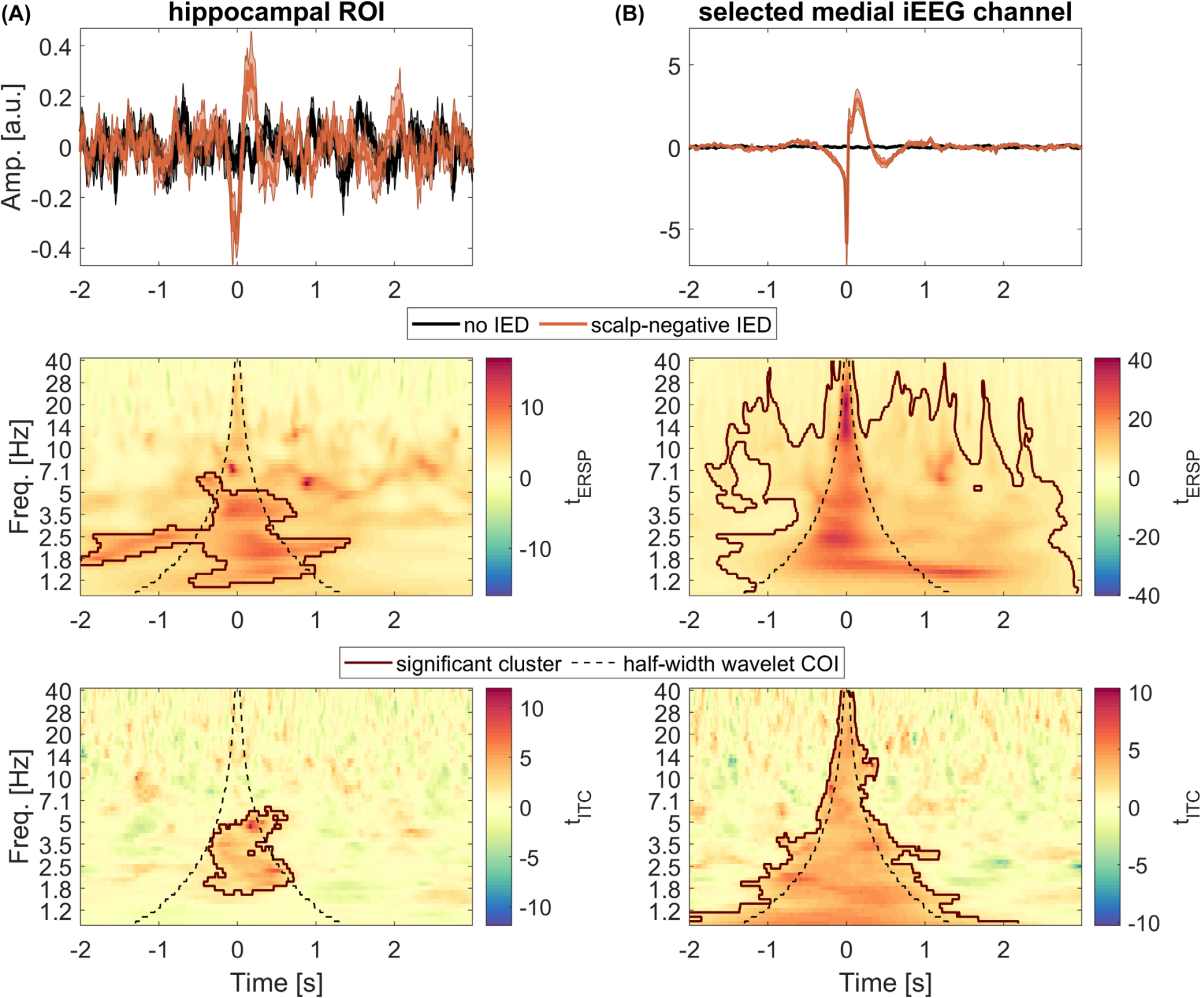
Temporal and time–frequency changes in hippocampal activity during scalp‐negative interictal epileptic discharges (IEDs) for both modalities (reconstructed from scalp electroencephalography [EEG] and intracranial EEG [iEEG]). The grand average is displayed in solid lines (shading shows standard error) for the no‐IED (A) and scalp‐negative IED conditions (B), in orange and black, respectively. The top time–frequency images correspond to the *t*‐statistics comparing scalp‐negative IED against no‐IED periods of the event‐related spectral perturbation (ERSP); the bottom ones correspond to the intertrial coherence (ITC). The significant clusters, obtained after cluster‐based statistics, are circumscribed by a dark orange line. The dashed lines correspond to the half‐width of the wavelet's cone of influence (COI) and serve as a visual reference. Both iEEG and reconstructed hippocampal signals show significant increase in broadband power and phase‐locked activity during scalp‐negative IEDs. a.u.: arbitrary unit; ROI, region of interest.

**FIGURE 4:**
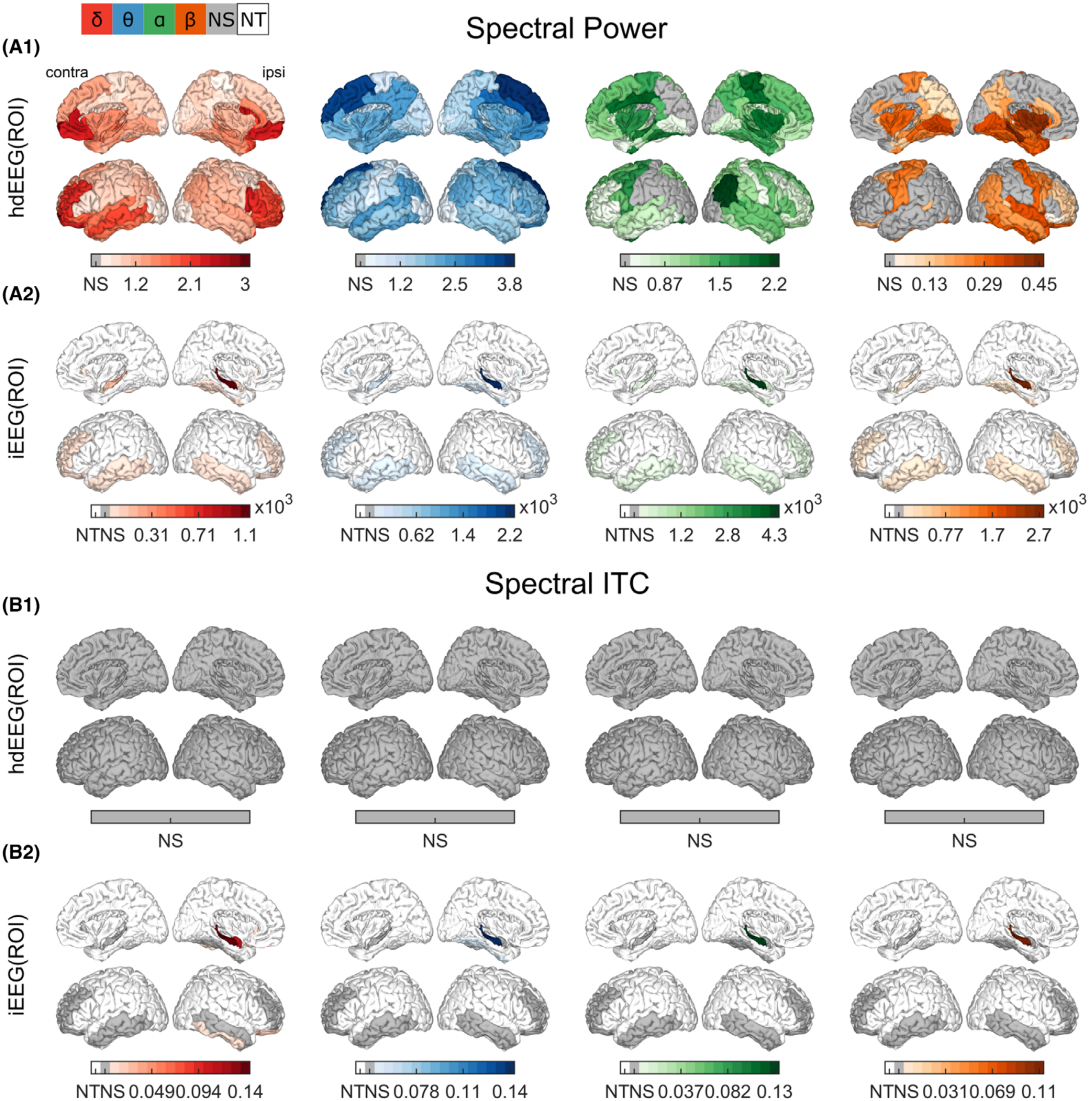
Regional changes in spectral power and intertrial coherence (ITC) for both modalities. For each region of interest (ROI), the median increase (scalp‐negative interictal epileptic discharge [IED]–no‐IED) across patients is reported for either spectral power (A) or ITC (B) computed on high‐density electroencephalography (hdEEG; .1) or intracranial EEG (iEEG; .2). Grayed ROIs are not significant (NS) after false discovery rate correction. White ROIs are those not tested (NT) because they were not sufficiently sampled by iEEG (fewer than five patients). The colors correspond to the four frequency bands under investigation. The darker the ROI, the stronger the increase during scalp‐negative IEDs. There is a broadband and large‐scale change in spectral power in both modalities, and there is a broadband hippocampus‐centered change in spectral ITC in iEEG during scalp‐negative IEDs.

Time‐locked ERSP and ITC analyses compared scalp‐negative IED epochs to no‐IED epochs (Figure 3). Using hdEEG, electrical source imaging was able to reveal time–frequency and phase‐locked changes associated with scalp‐negative IEDs. A significant positive cluster of ERSP (1–7 Hz, −2 s to 1.5 s, *p* < .001) and a significant positive cluster of ITC within the COI (1.8–7 Hz, *p* = .006) were found in the hippocampal ROI (Figure 3A). Whole‐brain analysis showed that the spatial distribution of ROIs with significant power changes (*p* < .05) varied across frequency bands (Figure 4A.1). Beta band increases were centered in ipsilateral medial and lateral temporal regions; theta and delta band changes were widespread, with greater involvement of the prefrontal areas; alpha and beta band maximum changes occurred in medial temporal regions and insula (Figure 4B.1).

iEEG confirmed these findings. In the selected medial temporal iEEG channel, ERSP showed a significant positive cluster (1–40 Hz, −1.5 to 3 s, *p* < .001) with maximum intensity centered around 0 s; ITC increases closely matched the half‐width of the COI of the wavelet (*p* < .001; Figure 3B). ERSP clustering presented maximum increase in the COI due to the selected IEDs, but the increase was also linked to activities occurring in temporal vicinity to the IEDs (possibly sharp activities accompanying the IEDs). iEEG(ROI) analysis revealed significant temporal lateral and anterior prefrontal power increases across all bands bilaterally, except for the orbitofrontal lateral gyri in beta band (*p* < .05; Figure 4A.2). The hippocampus showed the largest power increase across all bands, with the beta band showing 10 times higher power increase compared to other ROIs. For the ITC, only the hippocampus presented a significant increase in all frequency bands (*p* < .05; Figure 4B.2). The ipsilateral fusiform gyrus exhibited a significant increase in the theta band (*p* < .05), whereas the inferior temporal, lateral orbitofrontal, and amygdala regions increased in delta‐band ITC ipsilaterally (*p* < .05). Median differences and statistical details are provided in Tables S3–S6 for both modalities and frequency bands.

### 3.3 Network modification

**FIGURE 5:**
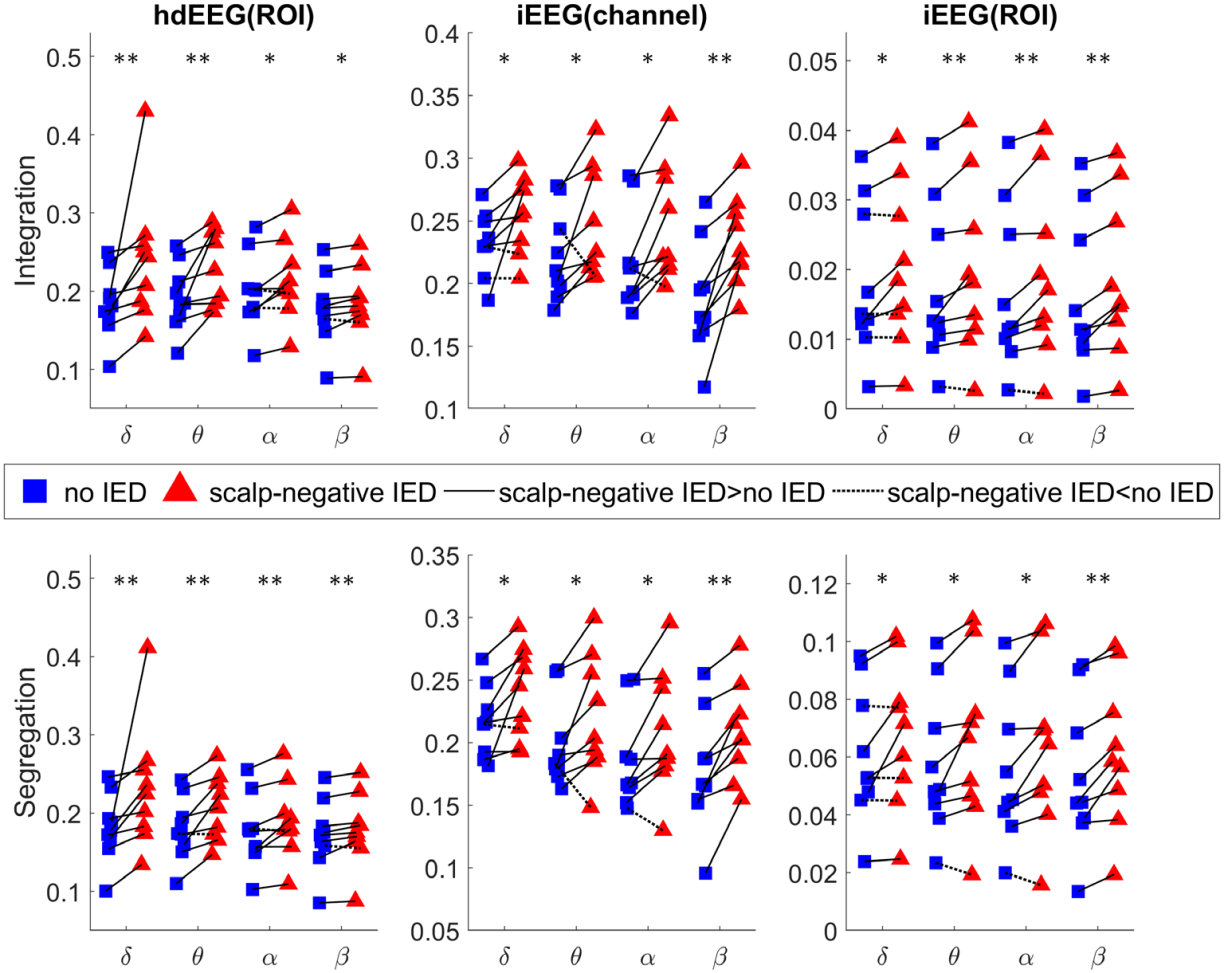
Scatterplots of the network integration and segregation computed for both modalities. The top and bottom rows represent the amount of network integration and segregation for each frequency and each patient, respectively. The network features are computed at either the region of interest (ROI) level using high‐density electroencephalography (hdEEG) or intracranial electroencephalography (iEEG; hdEEG[ROI], iEEG[ROI]), or iEEG channel level (iEEG[channel]). Red triangles depict the values of network features during scalp‐negative interictal epileptic discharges (IEDs) and blue squares during no‐IED. Lines connect the no‐IED and scalp‐negative IED values of the same patients. Lines are solid if values are higher during scalp‐negative IEDs and dotted otherwise. Significance of the signed‐rank test after false discovery rate correction is reported as follows: *p < .05, **p < .01.

Whole‐brain changes in integration and segregation during scalp‐negative IEDs compared to no‐IED period are depicted in Figure 5 (Table S7 details median differences for each frequency band and their respective *p*‐values). This analysis revealed a consistent increase in both integration and segregation across all frequency bands and modalities. These increases in either integration or segregation were observed in at least seven of nine patients for hdEEG(ROI) and iEEG(channel) and in six of nine patients for iEEG(ROI). The size of the IZ and the test scores were not correlated with these measures (uncorrected *p* > .03; Tables S8–S10). As these measures reflect the overall pathology, we did not expect them to explain the effect of the selected spatially limited IEDs. The test scores were significantly correlated with the size of the IZ, regardless of the type of score (Figure S2 and Table S11). In other words, the larger the IZ, the worse the cognitive performance.

**FIGURE 6:**
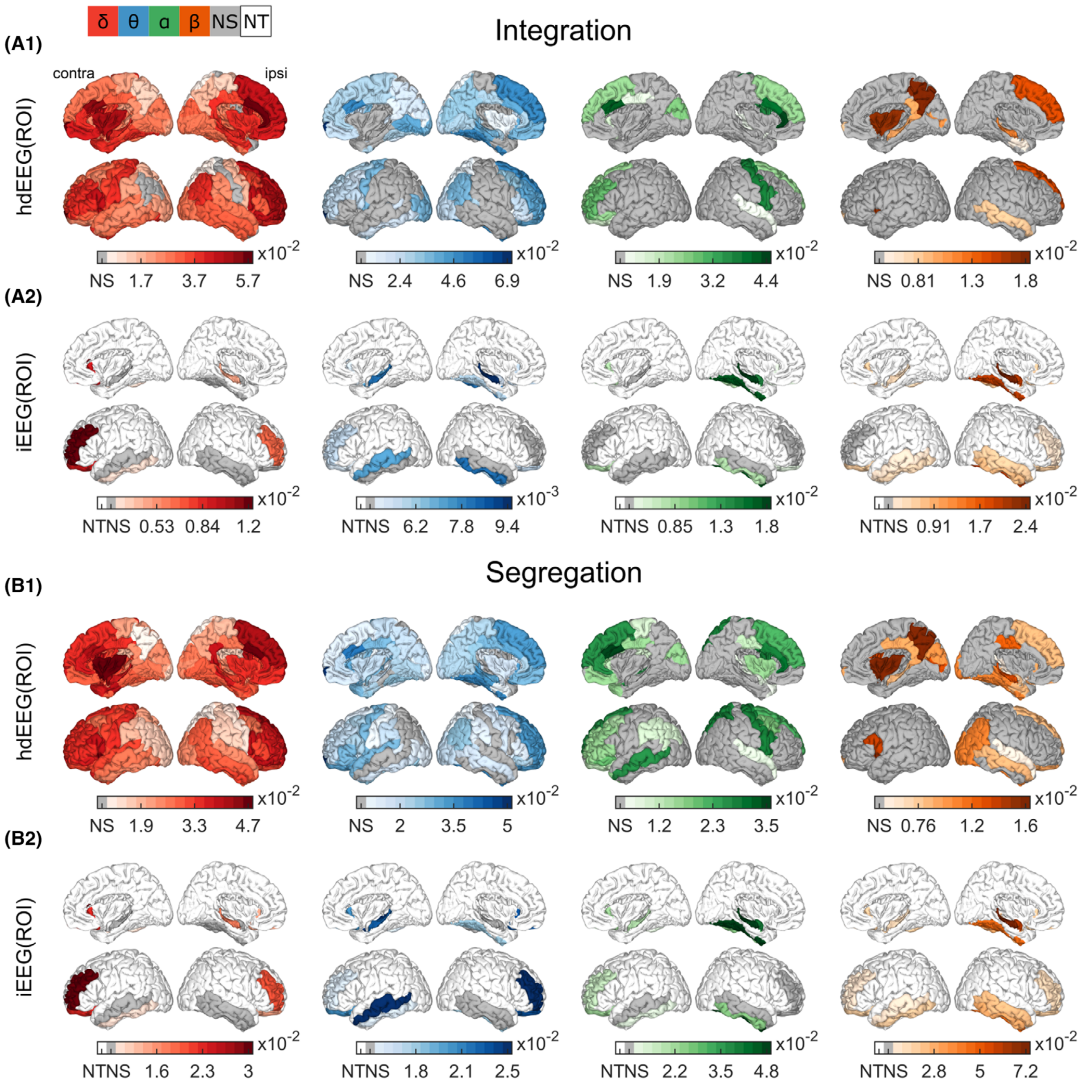
Regional changes in integration and segregation for both modalities. For each region of interest (ROI), the median difference (scalp‐negative interictal epileptic discharge [IED]—no‐IED) across patients is reported for either integration (A) or segregation (B) computed on high‐density electroencephalography (hdEEG; .1) or intracranial EEG (iEEG; .2). Gray‐colored ROIs are not significant (NS) after false discovery rate correction. White‐colored ROIs are those not tested (NT) because they were not sufficiently sampled by iEEG (fewer than five patients). The colors correspond to the four frequency bands under investigation. The darker the ROI, the stronger the increase during scalp‐negative IEDs. There is a broadband and large‐scale change in both modalities and both metrics.

ROI‐level results are illustrated in Figure 6 (median differences and *p*‐values are given in Tables S12–S15). For the hdEEG(ROI), significant concomitant increases in integration and segregation were observed in several ROIs, with a more focal modification in the beta and alpha bands and a more large‐scale change in the theta and delta bands. The significance of the ipsilateral hippocampus (*p* < .05) was consistent across all frequency bands and both measures, except for the alpha band. In the delta band, increases were more pronounced bilaterally in the anterior cingulate cortex, medial temporal and basal regions, insula, and prefrontal cortex. Theta‐band increases were greater bilaterally in the prefrontal and anterior cingulate regions, and in the medial temporal and basal regions ipsilaterally. Alpha‐band increases were bilateral in the anterior cingulate and superior prefrontal cortices, ipsilateral in the precentral gyrus, and contralateral in additional anterior prefrontal areas. Beta‐band changes were ipsilateral in the superior frontal and middle temporal gyri, and contralateral in the anterior insula, precuneus, and posterior cingulate.

For iEEG(ROI), the ipsilateral hippocampus showed significant increases (*p* < .05) in both integration and segregation for each frequency band, except in theta band for the segregation. Along with the hippocampus, other ROIs showed increased integration and/or segregation during scalp‐negative IEDs. Bilateral middle frontal and orbitofrontal gyri were significant in almost all frequency bands and measures. Interestingly, the temporal gyri were significant in the beta band bilaterally but less so in other frequency bands.

## 4 DISCUSSION

In this study, we investigated whether medial temporal IEDs that were not visible on scalp EEG influenced whole‐brain networks. To answer this question, we selected IEDs recorded only on iEEG and studied the simultaneous changes on hdEEG recordings. ROI time courses during IEDs reconstructed with hdEEG exhibited sharp wave characteristics, validating that some information reached the scalp at the exact time of iEEG IED. Our findings also revealed a frequency‐dependent large‐scale increase in power, integration, and segregation during scalp‐negative medial temporal IEDs compared to epochs without IED, at the whole‐brain level and within specific and consistent ROIs. Even though localized in the medial temporal regions, these IEDs exerted a nonuniform/nonlinear impact on remote brain regions, unveiling pathological large‐scale specific modifications of brain networks.

### 4.1 Isolating medial temporal IEDs not visible on the scalp

To ensure that our study focused solely on scalp‐negative IEDs originating from medial temporal regions, we rigorously validated our manual marking. Initially, an automated detector revealed that the majority of IEDs within the analyzed windows were concentrated in these regions. Additionally, in iEEG, the evoked time–frequency cluster had the characteristics of a sharp transient. The ipsilateral hippocampal ROI showed the largest power change and was the only region with significant phase‐locking across all frequencies. These arguments underscore that only the selected medial temporal IEDs were phase‐locked, reinforcing the validity of our marking procedure.

Although IEDs were not visible on scalp EEG, significant broadband evoked and induced clusters were found in the hippocampal ROI obtained with hdEEG and overlapped within the COI. Moreover, the change in power was more pronounced in the alpha and beta bands in the ipsilateral medial temporal ROIs. These findings indicate that information about IEDs reaches the surface, with reconstructed time‐locked activity capturing sharp transient features, consistent with previous studies.^7^, ^8^, ^27^, ^43^

### 4.2 Large‐scale network disruption by medial temporal IEDs

Our study shows that whole‐brain networks modifications due to spatially limited IEDs can be studied using simultaneous iEEG‐hdEEG recordings. Although confined to medial temporal regions, scalp‐negative IEDs have large‐scale effects on brain networks. We observed overall increases in spectral power, segregation, and integration, with these changes being more localized for high frequencies and more widespread for low frequencies. Notably, low‐frequency changes (delta, theta) predominated in prefrontal and temporolateral regions, whereas higher frequencies (alpha, beta) were more focused on areas connected to the medial temporal lobe, such as the insula, cingulate, and temporobasal structures. These hdEEG findings were validated by iEEG analyses in the sampled ROIs, reinforcing their robustness. These results expand upon previous studies demonstrating interictal network alterations even in absence of visible scalp IEDs, likely reflecting background pathology, scalp‐negative IEDs, or both.^9^, ^11^ Our study shows that epochs with scalp‐negative IEDs exhibit stronger network abnormalities than those without. Future work should disentangle their relative contributions on continuous recordings. Overall, our findings emphasize the importance of investigating “normal” recordings, void of visible epileptic activity.

Interictal cognitive deficits in temporal lobe epilepsy (TLE) extend beyond typical temporal functions, such as episodic and verbal memory and language,^19^, ^44^, ^45^ but also affect functions associated with widespread extratemporal networks, including executive functions.^45^, ^46^, ^47^ The transient disruption of large‐scale networks by medial temporal IEDs may contribute to interictal network dysfunction underlying cognitive deficits. This disruption could potentially culminate in paroxysmal transient cognitive impairment associated with IEDs in some patients.^48^ The role and clinical management of covert epileptic activity with predominantly (dys)cognitive manifestations represent an open issue in a range of brain disorders, including neurodegenerative (e.g., dementia) conditions.^19^, ^20^ Scalp‐negative IEDs, identified in Alzheimer disease via foramen ovale electrodes, were hypothesized to worsen cognition and disease progression, possibly through transient network disruptions.^21^ A deep learning algorithm developed to detect these IEDs in these patients^49^ might have identified these reported network changes without explicit supervision. Interestingly, a randomized controlled trial in Alzheimer patients with scalp IEDs showed that treatment with low‐dose antiseizure medication (levetiracetam) improved some cognitive performance measures (i.e., Stroop interference naming, virtual route learning).^50^ This suggests that counteracting the disruption of brain networks by IEDs could improve cognitive deficits. The identification of spatiotemporal EEG characteristics of subtle medial temporal interictal paroxysms could represent a potentially useful biomarker to evaluate their network impact and help guide clinical management. Although our study did not assess this directly, further simultaneous recording research linking cognition to IED‐driven network disruptions may help elucidate this phenomenon.

### 4.3 Methodological considerations

Several methodological points warrant discussion. First, the small sample size reflects the technical difficulty of simultaneous iEEG and hdEEG recordings. Larger cohorts could help replicate findings and refine the affected networks. Second, despite a significant ITC cluster in the hippocampal ROI, no significant phase‐locked activity was identified with hdEEG, possibly due to differences in ITC computation (time–frequency vs. spectral) and ITC being more stringent than ERSP. Third, we used information derived from iEEG as priors to identify scalp‐negative IED and no‐IED epochs. Further investigation will clarify whether these results can be obtained with iEEG‐naive hdEEG recordings and used for diagnostic purposes. Although IEDs and gamma activity are known to contribute to epilepsy, we did not analyze this frequency band, to limit the number of statistical corrections and because the SNR decreases sharply at higher frequencies. Moreover, connectivity analyses do not rely on trial averaging and thus do not benefit from SNR improvement. Because we used a within‐patient design, with no‐IED epochs serving as the baseline, we expect that any bias due to methodological factors such as filtering, wavelet selection, connectivity metrics, or arousal state would be equally present in both conditions and thus minimized. Lastly, due to the low number of left TLE, we analyzed the data as ipsilateral and contralateral. This prevented us from investigating the differential hemispheric effects of IEDs or TLE that have previously been reported.^17^ However, any lateralized effects would most likely be present in both modalities and not obscure our main findings.

## 5 CONCLUSIONS

Simultaneous iEEG and hdEEG recordings revealed that medial temporal IEDs invisible on scalp EEG can induce detectable time–frequency changes through source reconstruction and time‐locked analyses. These spatially limited IEDs also trigger widespread alterations in power and network organization. These findings support the development of methods to better detect subtle scalp EEG correlates of iEEG IEDs and assess their real‐time cognitive impact.

## AUTHOR CONTRIBUTIONS

*Conception and design of the study:* Nicolas Roehri, Shahan Momjian, Margitta Seeck, and Serge Vulliemoz. *Acquisition and analysis of data:* Nicolas Roehri, Pia De Stefano, Laurent Spinelli, Renaud Marquis, and Serge Vulliemoz. *Drafting a significant portion of the manuscript or figures:* Nicolas Roehri, Stanislas Lagarde, and Serge Vulliemoz.

## CONFLICT OF INTEREST STATEMENT

M.S. and S.V. have shares in Clouds of Care. N.R. holds an international patent for Delphos (EP3422935B1). The other authors report no competing interests. We confirm that we have read the Journal's position on issues involved in ethical publication and affirm that this report is consistent with those guidelines.

## SOCIAL MEDIA AND ARTICLE PROMOTION

Hidden medial temporal IEDs leave a large‐scale neural footprint, suggesting a covert contributor to cognitive impairment in epilepsy.

## Supporting information


**DATA S1** Supporting Information

## Data Availability

The raw data that support the findings of this study are available on reasonable request from the corresponding author. The raw data are not publicly available as they could contain information that could compromise the privacy of research participants. Derived data (e.g., connectivity matrices, derived network measures [integration and segregation] for each frequency band) as well as the results of the statistical tests are available here: https://figshare.com/s/a3f3f7c95d60cb48c295.
