# Supplementary material for: Scalp‐negative medial temporal interictal epileptic discharges alter large‐scale brain networks: A simultaneous high‐density electroencephalographic and intracranial electroencephalographic study
Source: Epilepsia. 2025 Dec 22;67(4):1992–2006. doi: 10.1002/epi.70061 (PMC13075616; doi:10.1002/epi.70061)
Supplement: Supplementary file 1 — DATA S1 Supporting Information [file EPI-67-1992-s001.docx]

**Scalp-Negative Medial Temporal Interictal Epileptic Discharges Alter Large-Scale Brain Networks: A Simultaneous High-Density EEG And Intracranial EEG Study**

**Authors:** Nicolas Roehri PhD^1^, Pia De Stefano MD^1,2^, Laurent Spinelli PhD^1^, Renaud Marquis PhD^1^, Stanislas Lagarde MD PhD^1,3^, Shahan Momjian MD^4^, Margitta Seeck MD^1^, Serge Vulliemoz MD PhD^1,5^

| **Patient** | **Sex** | **Age at epilepsy onset (Y)** | **Age at recording (Y)** | **Type of Epilepsy** | **MRI findings** | **Hemisphere with marked hippocampal IEDs** | **#analyzed IEDs** | **Electrode Manufacturer** | **IZ**  **(#ROI)** | **Delayed recall. Score (/15)** | |
| --- | --- | --- | --- | --- | --- | --- | --- | --- | --- | --- | --- |
|  |  |  |  |  |  |  |  |  |  | **Verbal** | **Non Verbal** |
| 1 | F | 14 | 27 | LPLE | FCD | L | 47 | AD-Tech | 8 | 14 | 13 |
| 2 | M | 36 | 48 | RTLE | None | R | 58 | AD-Tech | 12 | 5 | 14.5 |
| 3 | F | 25 | 53 | RTLE | None | R | 204 | AD-Tech | 8 | 15 | 13 |
| 4 | M | 35 | 38 | BTLE | None | R | 22 | AD-Tech | 13 | 3 | 13 |
| 5 | F | 17 | 42 | LTLE | HS | R | 40 | AD-Tech | 14 | 7 | 2 |
| 6 | M | 17 | 21 | BTLE | None | R | 57 | AD-Tech | 8 | 4 | 4.5 |
| 7* | M | 39 | 50 | BTLE | HS | L | 24 | AD-Tech | 13 | 8 | NA |
| 8 | M | 23 | 27 | RTLE | None | R | 49 | AD-Tech | 6 | 6 | 11.5 |
| 9 | M | 10 | 40 | LTLE | HS | R | 43 | Dixi | 21 | 1 | 1 |

**Supplementary Table 1:** Clinical details of the patients. *the only patient for whom the no IED and scalp-negative IED epochs were selected from N2 sleep stage. For all the others, the epochs were selected from awake recordings. Legend: M: male, F: female, Y: years, IED: interictal epileptic discharge, R: Right, L: Left, TLE: Temporal Lobe Epilepsy, BTLE: bilateral TLE, PLE: Parietal Lobe Epilepsy, FCD: Focal Cortical Dysplasia, HS: Hippocampal Sclerosis, IZ (#ROI): number of regions of interest in the irritative zone.

AD-Tech: 6-10 contacts of 2.4mm length, 1.1mm diameter and 2.6mm apart from each other.

Dixi Medical: 10–16 of 2mm length, 0.8mm diameter and 1.5mm apart from each other.

| **ROI name** | **patient** | **ROI name** | **patient** | **ROI name** | **patient** |
| --- | --- | --- | --- | --- | --- |
| lh-caudalmiddlefrontal | 1 | rh-fusiform | 5 | rh-lateralorbitofrontal | 9 |
| lh-supramarginal | 1 | rh-parahippocampal | 5 | rh-parstriangularis | 9 |
| lh-fusiform | 1 | rh-inferiortemporal | 5 | rh-rostralmiddlefrontal | 9 |
| lh-temporalpole | 1 | rh-middletemporal | 5 | rh-fusiform | 9 |
| lh-inferiortemporal | 1 | Right-Hippocampus | 5 | rh-parahippocampal | 9 |
| lh-middletemporal | 1 | Right-Amygdala | 5 | rh-entorhinal | 9 |
| Left-Hippocampus | 1 | lh-lateralorbitofrontal | 5 | rh-inferiortemporal | 9 |
| Left-Amygdala | 1 | lh-rostralanteriorcingulate | 5 | rh-middletemporal | 9 |
| rh-fusiform | 2 | lh-fusiform | 5 | rh-superiortemporal | 9 |
| rh-inferiortemporal | 2 | lh-parahippocampal | 5 | Right-Amygdala | 9 |
| rh-middletemporal | 2 | lh-entorhinal | 5 | lh-parsorbitalis | 9 |
| rh-superiortemporal | 2 | lh-temporalpole | 5 | lh-parstriangularis | 9 |
| Right-Hippocampus | 2 | lh-inferiortemporal | 5 | lh-superiorfrontal | 9 |
| Right-Amygdala | 2 | lh-middletemporal | 5 | lh-caudalmiddlefrontal | 9 |
| lh-fusiform | 2 | rh-middletemporal | 6 | lh-superiorparietal | 9 |
| lh-inferiortemporal | 2 | Right-Amygdala | 6 | lh-precuneus | 9 |
| lh-middletemporal | 2 | lh-entorhinal | 6 | lh-inferiortemporal | 9 |
| lh-insula | 2 | lh-inferiortemporal | 6 | lh-middletemporal | 9 |
| Left-Hippocampus | 2 | lh-middletemporal | 6 | lh-insula | 9 |
| Left-Amygdala | 2 | lh-insula | 6 | Left-Hippocampus | 9 |
| rh-parstriangularis | 3 | Left-Hippocampus | 6 | Left-Amygdala | 9 |
| rh-rostralmiddlefrontal | 3 | Left-Amygdala | 6 |  |  |
| rh-parahippocampal | 3 | rh-superiorfrontal | 7 |  |  |
| rh-inferiortemporal | 3 | rh-fusiform | 7 |  |  |
| rh-middletemporal | 3 | rh-inferiortemporal | 7 |  |  |
| rh-superiortemporal | 3 | rh-middletemporal | 7 |  |  |
| Right-Hippocampus | 3 | lh-superiorfrontal | 7 |  |  |
| Right-Amygdala | 3 | lh-precentral | 7 |  |  |
| rh-parsopercularis | 4 | lh-postcentral | 7 |  |  |
| rh-caudalmiddlefrontal | 4 | lh-superiorparietal | 7 |  |  |
| rh-fusiform | 4 | lh-fusiform | 7 |  |  |
| rh-parahippocampal | 4 | lh-entorhinal | 7 |  |  |
| rh-inferiortemporal | 4 | lh-inferiortemporal | 7 |  |  |
| Right-Hippocampus | 4 | lh-middletemporal | 7 |  |  |
| Right-Amygdala | 4 | Left-Hippocampus | 7 |  |  |
| lh-fusiform | 4 | rh-isthmuscingulate | 8 |  |  |
| lh-entorhinal | 4 | rh-fusiform | 8 |  |  |
| lh-inferiortemporal | 4 | rh-parahippocampal | 8 |  |  |
| lh-middletemporal | 4 | rh-inferiortemporal | 8 |  |  |
| Left-Hippocampus | 4 | Right-Hippocampus | 8 |  |  |
| Left-Amygdala | 4 | Right-Amygdala | 8 |  |  |

**Supplementary Table 2:** List of the region of interest belonging to the irritative zone of each patient. ROI names correspond to the ones of the Desikan atlas.
lh: left hemisphere, rh: right hemisphere, ROI: region of interest.

As a supplementary analysis, we performed network-based statistics (NBS)^1^ using 512 permutations (2^9^=512) to identify network component of increased connectivity. The component forming threshold was set to p<0.0025 for the delta frequency band and p<0.005 for the others, and the statistically significance was set to p<0.05 (Figure S1). NBS was used in addition to our primary regional analysis, as we did not expect consistent pairwise connectivity patterns across patients. However, we hypothesized that similar brain regions would be involved in the networks associated with scalp-negative IEDs, supporting the regional analysis.

The NBS revealed one significant network component in each frequency band, except in the beta band (Figure S1). The connectivity increased between the mesial ipsilateral temporal ROIs and the mesial and orbitofrontal ROIs in delta and alpha frequency band. In theta band, the connectivity increases mainly between the mesial and orbitofrontal ROIs and some parietal ROIs. These regions are in line with the results at the ROI-level.

1. Zalesky A, Fornito A, Bullmore ET. Network-based statistic: Identifying differences in brain networks. *Neuroimage*. 2010;53(4):1197-1207. doi:10.1016/j.neuroimage.2010.06.041


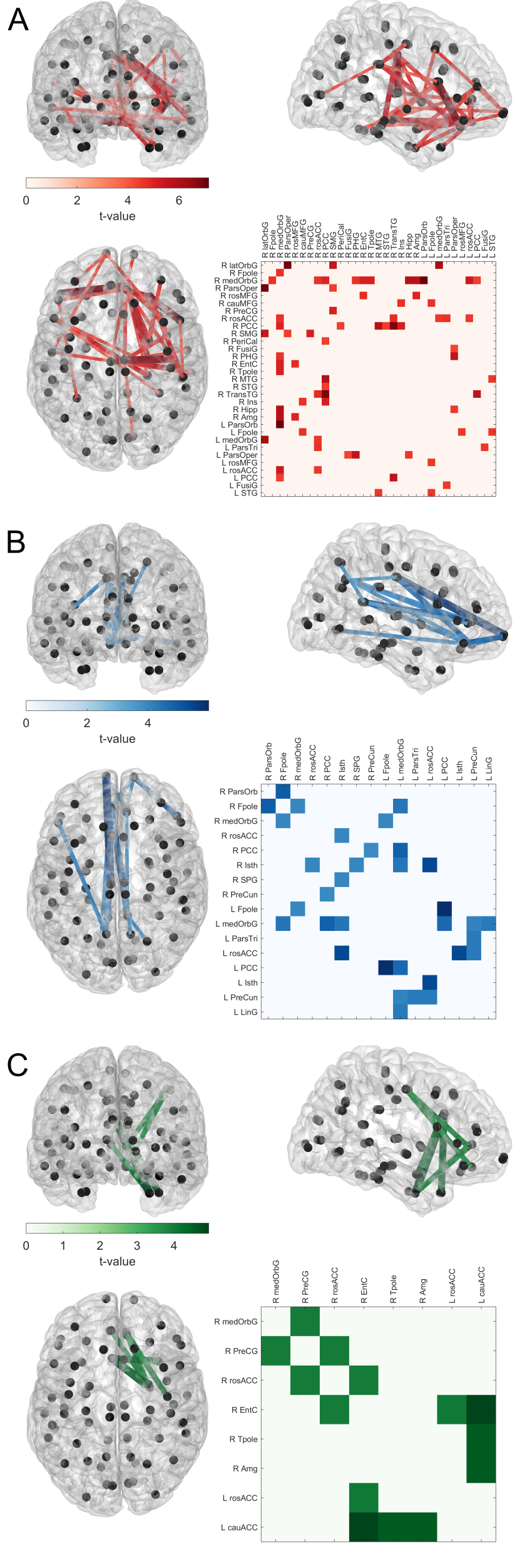
**Figure S1** **Increased network connectivity during scalp-negative IEDs vs no IEDs.** The dots correspond to the ROIs. For the sake of comparison across patients, the hemispheres of patients with selected scalp-negative IEDs originating from the left hemisphere were swapped. Comparisons of networks derived from hdEEG were obtained with the network-based statistics. The statistically significant network components for delta, theta and alpha are displayed in panel A, B and C, respectively. The connectivity increased between the medial ipsilateral temporal ROIs and the mesial and orbitofrontal ROIs in delta and alpha frequency band. In theta band, the connectivity increases mainly between the medial and orbitofrontal ROIs and some parietal ROIs. These results are in line with the results of Figure 6. IED: interictal epileptiform discharge; ROI: region of interest.

| Supplementary Table 3: hdEEG(ROI) power change between scalp-negative IEDs vs no IED | | | | | |
| --- | --- | --- | --- | --- | --- |
| Frequency band | **ROI name** | **Median difference** | **#patients IED>no IED** | **P‑value** | **Adjusted P‑value** |
| delta | rh-lateralorbitofrontal | 2.02 | 9 | 0.002 | **0.0033** |
| delta | rh-parsorbitalis | 1.99 | 9 | 0.002 | **0.0033** |
| delta | rh-frontalpole | 2.47 | 8 | 0.0039 | **0.0057** |
| delta | rh-medialorbitofrontal | 2.3 | 9 | 0.002 | **0.0033** |
| delta | rh-parstriangularis | 2.21 | 9 | 0.002 | **0.0033** |
| delta | rh-parsopercularis | 2.3 | 9 | 0.002 | **0.0033** |
| delta | rh-rostralmiddlefrontal | 1.92 | 9 | 0.002 | **0.0033** |
| delta | rh-superiorfrontal | 1.02 | 9 | 0.002 | **0.0033** |
| delta | rh-caudalmiddlefrontal | 0.86 | 9 | 0.002 | **0.0033** |
| delta | rh-precentral | 0.984 | 9 | 0.002 | **0.0033** |
| delta | rh-paracentral | 0.727 | 9 | 0.002 | **0.0033** |
| delta | rh-rostralanteriorcingulate | 1.61 | 9 | 0.002 | **0.0033** |
| delta | rh-caudalanteriorcingulate | 1.42 | 9 | 0.002 | **0.0033** |
| delta | rh-posteriorcingulate | 0.872 | 9 | 0.002 | **0.0033** |
| delta | rh-isthmuscingulate | 0.899 | 9 | 0.002 | **0.0033** |
| delta | rh-postcentral | 1.19 | 9 | 0.002 | **0.0033** |
| delta | rh-supramarginal | 1.08 | 9 | 0.002 | **0.0033** |
| delta | rh-superiorparietal | 0.988 | 9 | 0.002 | **0.0033** |
| delta | rh-inferiorparietal | 1.32 | 9 | 0.002 | **0.0033** |
| delta | rh-precuneus | 0.995 | 9 | 0.002 | **0.0033** |
| delta | rh-cuneus | 0.865 | 9 | 0.002 | **0.0033** |
| delta | rh-pericalcarine | 0.871 | 9 | 0.002 | **0.0033** |
| delta | rh-lateraloccipital | 0.682 | 9 | 0.002 | **0.0033** |
| delta | rh-lingual | 0.976 | 9 | 0.002 | **0.0033** |
| delta | rh-fusiform | 1.35 | 9 | 0.002 | **0.0033** |
| delta | rh-parahippocampal | 1.42 | 9 | 0.002 | **0.0033** |
| delta | rh-entorhinal | 1.57 | 9 | 0.002 | **0.0033** |
| delta | rh-temporalpole | 1.93 | 9 | 0.002 | **0.0033** |
| delta | rh-inferiortemporal | 1.18 | 9 | 0.002 | **0.0033** |
| delta | rh-middletemporal | 1.11 | 9 | 0.002 | **0.0033** |
| delta | rh-bankssts | 1.15 | 9 | 0.002 | **0.0033** |
| delta | rh-superiortemporal | 1.29 | 9 | 0.002 | **0.0033** |
| delta | rh-transversetemporal | 1.15 | 9 | 0.002 | **0.0033** |
| delta | rh-insula | 1.17 | 9 | 0.002 | **0.0033** |
| delta | Right-Hippocampus | 1.47 | 9 | 0.002 | **0.0033** |
| delta | Right-Amygdala | 1.35 | 9 | 0.002 | **0.0033** |
| delta | lh-lateralorbitofrontal | 2.16 | 9 | 0.002 | **0.0033** |
| delta | lh-parsorbitalis | 1.86 | 9 | 0.002 | **0.0033** |
| delta | lh-frontalpole | 2.99 | 9 | 0.002 | **0.0033** |
| delta | lh-medialorbitofrontal | 2.34 | 9 | 0.002 | **0.0033** |
| delta | lh-parstriangularis | 0.849 | 9 | 0.002 | **0.0033** |
| delta | lh-parsopercularis | 0.812 | 9 | 0.002 | **0.0033** |
| delta | lh-rostralmiddlefrontal | 2.19 | 9 | 0.002 | **0.0033** |
| delta | lh-superiorfrontal | 1.26 | 9 | 0.002 | **0.0033** |
| delta | lh-caudalmiddlefrontal | 1.09 | 9 | 0.002 | **0.0033** |
| delta | lh-precentral | 0.907 | 9 | 0.002 | **0.0033** |
| delta | lh-paracentral | 0.837 | 9 | 0.002 | **0.0033** |
| delta | lh-rostralanteriorcingulate | 2.53 | 9 | 0.002 | **0.0033** |
| delta | lh-caudalanteriorcingulate | 1.06 | 9 | 0.002 | **0.0033** |
| delta | lh-posteriorcingulate | 0.882 | 9 | 0.002 | **0.0033** |
| delta | lh-isthmuscingulate | 1.08 | 9 | 0.002 | **0.0033** |
| delta | lh-postcentral | 0.919 | 9 | 0.002 | **0.0033** |
| delta | lh-supramarginal | 0.993 | 8 | 0.0039 | **0.0057** |
| delta | lh-superiorparietal | 0.982 | 9 | 0.002 | **0.0033** |
| delta | lh-inferiorparietal | 1.14 | 9 | 0.002 | **0.0033** |
| delta | lh-precuneus | 1.06 | 9 | 0.002 | **0.0033** |
| delta | lh-cuneus | 1.02 | 9 | 0.002 | **0.0033** |
| delta | lh-pericalcarine | 0.797 | 9 | 0.002 | **0.0033** |
| delta | lh-lateraloccipital | 0.649 | 8 | 0.0039 | **0.0057** |
| delta | lh-lingual | 1.34 | 8 | 0.0039 | **0.0057** |
| delta | lh-fusiform | 1.17 | 9 | 0.002 | **0.0033** |
| delta | lh-parahippocampal | 1.48 | 9 | 0.002 | **0.0033** |
| delta | lh-entorhinal | 1.35 | 9 | 0.002 | **0.0033** |
| delta | lh-temporalpole | 1.46 | 9 | 0.002 | **0.0033** |
| delta | lh-inferiortemporal | 1.64 | 9 | 0.002 | **0.0033** |
| delta | lh-middletemporal | 1.92 | 9 | 0.002 | **0.0033** |
| delta | lh-bankssts | 1.11 | 9 | 0.002 | **0.0033** |
| delta | lh-superiortemporal | 1.58 | 9 | 0.002 | **0.0033** |
| delta | lh-transversetemporal | 1.49 | 9 | 0.002 | **0.0033** |
| delta | lh-insula | 1.5 | 9 | 0.002 | **0.0033** |
| delta | Left-Hippocampus | 1.52 | 9 | 0.002 | **0.0033** |
| delta | Left-Amygdala | 1.42 | 9 | 0.002 | **0.0033** |
| theta | rh-lateralorbitofrontal | 2.6 | 9 | 0.002 | **0.0033** |
| theta | rh-parsorbitalis | 1.48 | 9 | 0.002 | **0.0033** |
| theta | rh-frontalpole | 1.91 | 9 | 0.002 | **0.0033** |
| theta | rh-medialorbitofrontal | 2.53 | 9 | 0.002 | **0.0033** |
| theta | rh-parstriangularis | 2.05 | 9 | 0.002 | **0.0033** |
| theta | rh-parsopercularis | 1.33 | 9 | 0.002 | **0.0033** |
| theta | rh-rostralmiddlefrontal | 1.96 | 9 | 0.002 | **0.0033** |
| theta | rh-superiorfrontal | 3.78 | 9 | 0.002 | **0.0033** |
| theta | rh-caudalmiddlefrontal | 2.19 | 9 | 0.002 | **0.0033** |
| theta | rh-precentral | 1.8 | 9 | 0.002 | **0.0033** |
| theta | rh-paracentral | 1.42 | 9 | 0.002 | **0.0033** |
| theta | rh-rostralanteriorcingulate | 2.63 | 9 | 0.002 | **0.0033** |
| theta | rh-caudalanteriorcingulate | 3.4 | 9 | 0.002 | **0.0033** |
| theta | rh-posteriorcingulate | 3.12 | 9 | 0.002 | **0.0033** |
| theta | rh-isthmuscingulate | 2.24 | 9 | 0.002 | **0.0033** |
| theta | rh-postcentral | 2.17 | 9 | 0.002 | **0.0033** |
| theta | rh-supramarginal | 1.95 | 9 | 0.002 | **0.0033** |
| theta | rh-superiorparietal | 1.4 | 8 | 0.0039 | **0.0057** |
| theta | rh-inferiorparietal | 2.18 | 9 | 0.002 | **0.0033** |
| theta | rh-precuneus | 1.52 | 9 | 0.002 | **0.0033** |
| theta | rh-cuneus | 1.35 | 9 | 0.002 | **0.0033** |
| theta | rh-pericalcarine | 0.957 | 9 | 0.002 | **0.0033** |
| theta | rh-lateraloccipital | 0.456 | 9 | 0.002 | **0.0033** |
| theta | rh-lingual | 1.44 | 9 | 0.002 | **0.0033** |
| theta | rh-fusiform | 1.95 | 9 | 0.002 | **0.0033** |
| theta | rh-parahippocampal | 2.37 | 9 | 0.002 | **0.0033** |
| theta | rh-entorhinal | 1.89 | 9 | 0.002 | **0.0033** |
| theta | rh-temporalpole | 1.69 | 9 | 0.002 | **0.0033** |
| theta | rh-inferiortemporal | 1.61 | 9 | 0.002 | **0.0033** |
| theta | rh-middletemporal | 1.67 | 9 | 0.002 | **0.0033** |
| theta | rh-bankssts | 1.01 | 9 | 0.002 | **0.0033** |
| theta | rh-superiortemporal | 1.52 | 9 | 0.002 | **0.0033** |
| theta | rh-transversetemporal | 1.57 | 8 | 0.0039 | **0.0057** |
| theta | rh-insula | 2.09 | 9 | 0.002 | **0.0033** |
| theta | Right-Hippocampus | 2.34 | 9 | 0.002 | **0.0033** |
| theta | Right-Amygdala | 1.99 | 9 | 0.002 | **0.0033** |
| theta | lh-lateralorbitofrontal | 1.7 | 9 | 0.002 | **0.0033** |
| theta | lh-parsorbitalis | 1.38 | 9 | 0.002 | **0.0033** |
| theta | lh-frontalpole | 1.63 | 9 | 0.002 | **0.0033** |
| theta | lh-medialorbitofrontal | 1.94 | 9 | 0.002 | **0.0033** |
| theta | lh-parstriangularis | 1.13 | 9 | 0.002 | **0.0033** |
| theta | lh-parsopercularis | 1.3 | 8 | 0.0039 | **0.0057** |
| theta | lh-rostralmiddlefrontal | 1.84 | 9 | 0.002 | **0.0033** |
| theta | lh-superiorfrontal | 3.6 | 9 | 0.002 | **0.0033** |
| theta | lh-caudalmiddlefrontal | 2.08 | 9 | 0.002 | **0.0033** |
| theta | lh-precentral | 1.33 | 8 | 0.0039 | **0.0057** |
| theta | lh-paracentral | 1 | 9 | 0.002 | **0.0033** |
| theta | lh-rostralanteriorcingulate | 2.16 | 9 | 0.002 | **0.0033** |
| theta | lh-caudalanteriorcingulate | 3.52 | 9 | 0.002 | **0.0033** |
| theta | lh-posteriorcingulate | 2.42 | 9 | 0.002 | **0.0033** |
| theta | lh-isthmuscingulate | 2.3 | 8 | 0.0059 | **0.0082** |
| theta | lh-postcentral | 0.768 | 7 | 0.0098 | **0.013** |
| theta | lh-supramarginal | 1.52 | 8 | 0.0039 | **0.0057** |
| theta | lh-superiorparietal | 1.25 | 8 | 0.02 | **0.024** |
| theta | lh-inferiorparietal | 2.41 | 8 | 0.0098 | **0.013** |
| theta | lh-precuneus | 2.27 | 9 | 0.002 | **0.0033** |
| theta | lh-cuneus | 1.22 | 8 | 0.0039 | **0.0057** |
| theta | lh-pericalcarine | 1.09 | 8 | 0.0059 | **0.0082** |
| theta | lh-lateraloccipital | 0.62 | 8 | 0.0039 | **0.0057** |
| theta | lh-lingual | 1.37 | 9 | 0.002 | **0.0033** |
| theta | lh-fusiform | 2.22 | 9 | 0.002 | **0.0033** |
| theta | lh-parahippocampal | 1.97 | 9 | 0.002 | **0.0033** |
| theta | lh-entorhinal | 2.07 | 9 | 0.002 | **0.0033** |
| theta | lh-temporalpole | 1.97 | 9 | 0.002 | **0.0033** |
| theta | lh-inferiortemporal | 2.02 | 9 | 0.002 | **0.0033** |
| theta | lh-middletemporal | 1.62 | 9 | 0.002 | **0.0033** |
| theta | lh-bankssts | 1.79 | 9 | 0.002 | **0.0033** |
| theta | lh-superiortemporal | 2.41 | 9 | 0.002 | **0.0033** |
| theta | lh-transversetemporal | 2.35 | 9 | 0.002 | **0.0033** |
| theta | lh-insula | 2.1 | 9 | 0.002 | **0.0033** |
| theta | Left-Hippocampus | 2.03 | 9 | 0.002 | **0.0033** |
| theta | Left-Amygdala | 2.24 | 9 | 0.002 | **0.0033** |
| alpha | rh-lateralorbitofrontal | 1.07 | 9 | 0.002 | **0.0033** |
| alpha | rh-parsorbitalis | 0.776 | 8 | 0.0059 | **0.0082** |
| alpha | rh-frontalpole | 0.687 | 8 | 0.0059 | **0.0082** |
| alpha | rh-medialorbitofrontal | 1.05 | 9 | 0.002 | **0.0033** |
| alpha | rh-parstriangularis | 1.31 | 7 | 0.02 | **0.024** |
| alpha | rh-parsopercularis | 1.03 | 9 | 0.002 | **0.0033** |
| alpha | rh-rostralmiddlefrontal | 0.736 | 9 | 0.002 | **0.0033** |
| alpha | rh-superiorfrontal | 1.36 | 8 | 0.0098 | **0.013** |
| alpha | rh-caudalmiddlefrontal | 0.685 | 9 | 0.002 | **0.0033** |
| alpha | rh-precentral | 1.3 | 8 | 0.0039 | **0.0057** |
| alpha | rh-paracentral | 2.12 | 9 | 0.002 | **0.0033** |
| alpha | rh-rostralanteriorcingulate | 0.979 | 9 | 0.002 | **0.0033** |
| alpha | rh-caudalanteriorcingulate | 1.44 | 9 | 0.002 | **0.0033** |
| alpha | rh-posteriorcingulate | 1.97 | 9 | 0.002 | **0.0033** |
| alpha | rh-isthmuscingulate | 1.2 | 8 | 0.0039 | **0.0057** |
| alpha | rh-postcentral | 0.581 | 8 | 0.027 | **0.033** |
| alpha | rh-supramarginal | 1.08 | 9 | 0.002 | **0.0033** |
| alpha | rh-superiorparietal | 1.23 | 8 | 0.014 | **0.017** |
| alpha | rh-inferiorparietal | 2.21 | 8 | 0.0039 | **0.0057** |
| alpha | rh-precuneus | 1.44 | 8 | 0.0098 | **0.013** |
| alpha | rh-cuneus | 1.94 | 7 | 0.13 | 0.13 |
| alpha | rh-pericalcarine | 0.749 | 7 | 0.064 | 0.071 |
| alpha | rh-lateraloccipital | 0.274 | 6 | 0.37 | 0.37 |
| alpha | rh-lingual | 0.747 | 9 | 0.002 | **0.0033** |
| alpha | rh-fusiform | 1.5 | 9 | 0.002 | **0.0033** |
| alpha | rh-parahippocampal | 1.52 | 8 | 0.0039 | **0.0057** |
| alpha | rh-entorhinal | 1.66 | 8 | 0.0098 | **0.013** |
| alpha | rh-temporalpole | 1.05 | 8 | 0.0039 | **0.0057** |
| alpha | rh-inferiortemporal | 0.998 | 7 | 0.0098 | **0.013** |
| alpha | rh-middletemporal | 1.32 | 8 | 0.0059 | **0.0082** |
| alpha | rh-bankssts | 1.74 | 9 | 0.002 | **0.0033** |
| alpha | rh-superiortemporal | 1.3 | 8 | 0.0039 | **0.0057** |
| alpha | rh-transversetemporal | 1.79 | 8 | 0.0098 | **0.013** |
| alpha | rh-insula | 1.91 | 9 | 0.002 | **0.0033** |
| alpha | Right-Hippocampus | 1.84 | 8 | 0.0059 | **0.0082** |
| alpha | Right-Amygdala | 1.54 | 8 | 0.0059 | **0.0082** |
| alpha | lh-lateralorbitofrontal | 0.9 | 9 | 0.002 | **0.0033** |
| alpha | lh-parsorbitalis | 0.662 | 7 | 0.027 | **0.033** |
| alpha | lh-frontalpole | 0.915 | 9 | 0.002 | **0.0033** |
| alpha | lh-medialorbitofrontal | 1.1 | 9 | 0.002 | **0.0033** |
| alpha | lh-parstriangularis | 0.506 | 9 | 0.002 | **0.0033** |
| alpha | lh-parsopercularis | 1.31 | 9 | 0.002 | **0.0033** |
| alpha | lh-rostralmiddlefrontal | 0.668 | 9 | 0.002 | **0.0033** |
| alpha | lh-superiorfrontal | 1.35 | 9 | 0.002 | **0.0033** |
| alpha | lh-caudalmiddlefrontal | 1.61 | 8 | 0.0039 | **0.0057** |
| alpha | lh-precentral | 1.78 | 8 | 0.014 | **0.017** |
| alpha | lh-paracentral | 1.58 | 8 | 0.02 | **0.024** |
| alpha | lh-rostralanteriorcingulate | 1.24 | 9 | 0.002 | **0.0033** |
| alpha | lh-caudalanteriorcingulate | 1.92 | 9 | 0.002 | **0.0033** |
| alpha | lh-posteriorcingulate | 2.09 | 8 | 0.02 | **0.024** |
| alpha | lh-isthmuscingulate | 1.53 | 8 | 0.037 | **0.043** |
| alpha | lh-postcentral | 1.04 | 6 | 0.1 | 0.11 |
| alpha | lh-supramarginal | 0.572 | 5 | 0.13 | 0.13 |
| alpha | lh-superiorparietal | 1.18 | 8 | 0.02 | **0.024** |
| alpha | lh-inferiorparietal | 2.84 | 7 | 0.049 | 0.055 |
| alpha | lh-precuneus | 1.44 | 7 | 0.082 | 0.088 |
| alpha | lh-cuneus | 2.26 | 7 | 0.082 | 0.088 |
| alpha | lh-pericalcarine | 0.974 | 6 | 0.082 | 0.088 |
| alpha | lh-lateraloccipital | 0.319 | 6 | 0.21 | 0.22 |
| alpha | lh-lingual | 0.67 | 8 | 0.0039 | **0.0057** |
| alpha | lh-fusiform | 1.86 | 9 | 0.002 | **0.0033** |
| alpha | lh-parahippocampal | 1.36 | 9 | 0.002 | **0.0033** |
| alpha | lh-entorhinal | 0.738 | 9 | 0.002 | **0.0033** |
| alpha | lh-temporalpole | 0.502 | 7 | 0.0098 | **0.013** |
| alpha | lh-inferiortemporal | 0.677 | 8 | 0.02 | **0.024** |
| alpha | lh-middletemporal | 0.863 | 8 | 0.0098 | **0.013** |
| alpha | lh-bankssts | 0.766 | 7 | 0.049 | 0.055 |
| alpha | lh-superiortemporal | 0.956 | 9 | 0.002 | **0.0033** |
| alpha | lh-transversetemporal | 1.72 | 7 | 0.014 | **0.017** |
| alpha | lh-insula | 1.73 | 8 | 0.0039 | **0.0057** |
| alpha | Left-Hippocampus | 1.79 | 8 | 0.0039 | **0.0057** |
| alpha | Left-Amygdala | 1.45 | 9 | 0.002 | **0.0033** |
| beta | rh-lateralorbitofrontal | 0.317 | 9 | 0.002 | **0.0033** |
| beta | rh-parsorbitalis | 0.0459 | 7 | 0.014 | **0.017** |
| beta | rh-frontalpole | 0.0798 | 7 | 0.13 | 0.13 |
| beta | rh-medialorbitofrontal | 0.218 | 7 | 0.082 | 0.088 |
| beta | rh-parstriangularis | 0.0909 | 6 | 0.1 | 0.11 |
| beta | rh-parsopercularis | 0.212 | 9 | 0.002 | **0.0033** |
| beta | rh-rostralmiddlefrontal | 0.146 | 7 | 0.037 | **0.043** |
| beta | rh-superiorfrontal | 0.0337 | 5 | 0.33 | 0.33 |
| beta | rh-caudalmiddlefrontal | 0.259 | 9 | 0.002 | **0.0033** |
| beta | rh-precentral | 0.296 | 8 | 0.037 | **0.043** |
| beta | rh-paracentral | 0.147 | 7 | 0.082 | 0.088 |
| beta | rh-rostralanteriorcingulate | 0.194 | 7 | 0.037 | **0.043** |
| beta | rh-caudalanteriorcingulate | 0.155 | 8 | 0.0098 | **0.013** |
| beta | rh-posteriorcingulate | 0.146 | 6 | 0.064 | 0.071 |
| beta | rh-isthmuscingulate | 0.233 | 9 | 0.002 | **0.0033** |
| beta | rh-postcentral | 0.0614 | 6 | 0.082 | 0.088 |
| beta | rh-supramarginal | 0.0954 | 6 | 0.064 | 0.071 |
| beta | rh-superiorparietal | 0.128 | 5 | 0.13 | 0.13 |
| beta | rh-inferiorparietal | 0.23 | 7 | 0.02 | **0.024** |
| beta | rh-precuneus | 0.149 | 7 | 0.037 | **0.043** |
| beta | rh-cuneus | -0.0399 | 4 | 0.46 | 0.46 |
| beta | rh-pericalcarine | 0.183 | 6 | 0.064 | 0.071 |
| beta | rh-lateraloccipital | 0.0682 | 7 | 0.1 | 0.11 |
| beta | rh-lingual | 0.346 | 7 | 0.014 | **0.017** |
| beta | rh-fusiform | 0.307 | 9 | 0.002 | **0.0033** |
| beta | rh-parahippocampal | 0.45 | 9 | 0.002 | **0.0033** |
| beta | rh-entorhinal | 0.298 | 9 | 0.002 | **0.0033** |
| beta | rh-temporalpole | 0.336 | 9 | 0.002 | **0.0033** |
| beta | rh-inferiortemporal | 0.319 | 6 | 0.027 | **0.033** |
| beta | rh-middletemporal | 0.218 | 8 | 0.0039 | **0.0057** |
| beta | rh-bankssts | 0.141 | 7 | 0.014 | **0.017** |
| beta | rh-superiortemporal | 0.329 | 9 | 0.002 | **0.0033** |
| beta | rh-transversetemporal | 0.29 | 8 | 0.0039 | **0.0057** |
| beta | rh-insula | 0.42 | 9 | 0.002 | **0.0033** |
| beta | Right-Hippocampus | 0.268 | 9 | 0.002 | **0.0033** |
| beta | Right-Amygdala | 0.285 | 9 | 0.002 | **0.0033** |
| beta | lh-lateralorbitofrontal | 0.149 | 8 | 0.037 | **0.043** |
| beta | lh-parsorbitalis | 0.0084 | 5 | 0.46 | 0.46 |
| beta | lh-frontalpole | 0.115 | 6 | 0.18 | 0.18 |
| beta | lh-medialorbitofrontal | 0.18 | 8 | 0.049 | 0.055 |
| beta | lh-parstriangularis | 0.135 | 6 | 0.15 | 0.15 |
| beta | lh-parsopercularis | 0.125 | 7 | 0.1 | 0.11 |
| beta | lh-rostralmiddlefrontal | 0.128 | 6 | 0.15 | 0.15 |
| beta | lh-superiorfrontal | 0.128 | 7 | 0.064 | 0.071 |
| beta | lh-caudalmiddlefrontal | 0.177 | 7 | 0.037 | **0.043** |
| beta | lh-precentral | 0.275 | 6 | 0.037 | **0.043** |
| beta | lh-paracentral | 0.242 | 8 | 0.014 | **0.017** |
| beta | lh-rostralanteriorcingulate | 0.122 | 8 | 0.049 | 0.055 |
| beta | lh-caudalanteriorcingulate | 0.21 | 8 | 0.014 | **0.017** |
| beta | lh-posteriorcingulate | 0.151 | 7 | 0.064 | 0.071 |
| beta | lh-isthmuscingulate | 0.201 | 9 | 0.002 | **0.0033** |
| beta | lh-postcentral | 0.238 | 8 | 0.0059 | **0.0082** |
| beta | lh-supramarginal | 0.17 | 7 | 0.15 | 0.15 |
| beta | lh-superiorparietal | 0.0908 | 6 | 0.33 | 0.33 |
| beta | lh-inferiorparietal | 0.103 | 6 | 0.1 | 0.11 |
| beta | lh-precuneus | 0.106 | 7 | 0.027 | **0.033** |
| beta | lh-cuneus | 0.0956 | 8 | 0.037 | **0.043** |
| beta | lh-pericalcarine | 0.208 | 8 | 0.0059 | **0.0082** |
| beta | lh-lateraloccipital | 0.0777 | 6 | 0.13 | 0.13 |
| beta | lh-lingual | 0.375 | 7 | 0.0098 | **0.013** |
| beta | lh-fusiform | 0.261 | 7 | 0.02 | **0.024** |
| beta | lh-parahippocampal | 0.296 | 9 | 0.002 | **0.0033** |
| beta | lh-entorhinal | 0.133 | 7 | 0.0098 | **0.013** |
| beta | lh-temporalpole | 0.147 | 8 | 0.049 | 0.055 |
| beta | lh-inferiortemporal | 0.127 | 6 | 0.082 | 0.088 |
| beta | lh-middletemporal | 0.116 | 7 | 0.049 | 0.055 |
| beta | lh-bankssts | 0.173 | 7 | 0.02 | **0.024** |
| beta | lh-superiortemporal | 0.0766 | 6 | 0.1 | 0.11 |
| beta | lh-transversetemporal | 0.282 | 9 | 0.002 | **0.0033** |
| beta | lh-insula | 0.291 | 7 | 0.0098 | **0.013** |
| beta | Left-Hippocampus | 0.224 | 9 | 0.002 | **0.0033** |
| beta | Left-Amygdala | 0.159 | 8 | 0.0039 | **0.0057** |
| #patients IED>no IED: number of patients for which the value in the ROI was higher in the scalp negative IED than the no IED condition. ROI names correspond to the ones of the Desikan atlas.  lh: left hemisphere, rh: right hemisphere, ROI: region of interest. | | | | | |

| Supplementary Table 4: iEEG(ROI) power change between scalp-negative IEDs vs rest | | | | | |
| --- | --- | --- | --- | --- | --- |
| Frequency band | **ROI name** | **Median difference** | **#patients IED>no IED** | **P‑value** | **Adjusted P‑value** |
| delta | rh-lateralorbitofrontal | 2.59 | 8 | 0.0039 | **0.0099** |
| delta | rh-rostralmiddlefrontal | 6.26 | 8 | 0.0039 | **0.0099** |
| delta | rh-fusiform | 28.3 | 7 | 0.0078 | **0.013** |
| delta | rh-inferiortemporal | 27.4 | 8 | 0.014 | **0.018** |
| delta | rh-middletemporal | 27.4 | 8 | 0.014 | **0.018** |
| delta | Right-Hippocampus | 1.10E+03 | 8 | 0.0039 | **0.0099** |
| delta | Right-Amygdala | 14.1 | 8 | 0.0039 | **0.0099** |
| delta | lh-lateralorbitofrontal | 2 | 6 | 0.016 | **0.018** |
| delta | lh-rostralmiddlefrontal | 7.96 | 6 | 0.016 | **0.018** |
| delta | lh-inferiortemporal | 20.1 | 7 | 0.0078 | **0.013** |
| delta | lh-middletemporal | 20.8 | 7 | 0.0078 | **0.013** |
| delta | Left-Hippocampus | 123 | 6 | 0.016 | **0.018** |
| theta | rh-lateralorbitofrontal | 9.41 | 8 | 0.0039 | **0.0099** |
| theta | rh-rostralmiddlefrontal | 14.7 | 9 | 0.002 | **0.0099** |
| theta | rh-fusiform | 55.1 | 7 | 0.0078 | **0.013** |
| theta | rh-inferiortemporal | 56.5 | 9 | 0.002 | **0.0099** |
| theta | rh-middletemporal | 67.1 | 9 | 0.002 | **0.0099** |
| theta | Right-Hippocampus | 2.16E+03 | 8 | 0.0039 | **0.0099** |
| theta | Right-Amygdala | 22.4 | 8 | 0.0039 | **0.0099** |
| theta | lh-lateralorbitofrontal | 2.97 | 6 | 0.016 | **0.018** |
| theta | lh-rostralmiddlefrontal | 12.1 | 5 | 0.031 | **0.034** |
| theta | lh-inferiortemporal | 10.3 | 7 | 0.0078 | **0.013** |
| theta | lh-middletemporal | 21.5 | 7 | 0.0078 | **0.013** |
| theta | Left-Hippocampus | 148 | 6 | 0.016 | **0.018** |
| alpha | rh-lateralorbitofrontal | 3.41 | 7 | 0.027 | **0.031** |
| alpha | rh-rostralmiddlefrontal | 15.2 | 9 | 0.002 | **0.0099** |
| alpha | rh-fusiform | 67.9 | 7 | 0.0078 | **0.013** |
| alpha | rh-inferiortemporal | 26.9 | 9 | 0.002 | **0.0099** |
| alpha | rh-middletemporal | 56.3 | 9 | 0.002 | **0.0099** |
| alpha | Right-Hippocampus | 4.29E+03 | 8 | 0.0039 | **0.0099** |
| alpha | Right-Amygdala | 21.9 | 8 | 0.0039 | **0.0099** |
| alpha | lh-lateralorbitofrontal | 0.909 | 6 | 0.016 | **0.018** |
| alpha | lh-rostralmiddlefrontal | 5.61 | 6 | 0.016 | **0.018** |
| alpha | lh-inferiortemporal | 9.91 | 7 | 0.0078 | **0.013** |
| alpha | lh-middletemporal | 17.7 | 7 | 0.0078 | **0.013** |
| alpha | Left-Hippocampus | 75.6 | 6 | 0.016 | **0.018** |
| beta | rh-lateralorbitofrontal | 0.283 | 6 | 0.27 | 0.27 |
| beta | rh-rostralmiddlefrontal | 0.911 | 7 | 0.0098 | **0.015** |
| beta | rh-fusiform | 34.6 | 7 | 0.0078 | **0.013** |
| beta | rh-inferiortemporal | 6.49 | 8 | 0.0039 | **0.0099** |
| beta | rh-middletemporal | 2.67 | 9 | 0.002 | **0.0099** |
| beta | Right-Hippocampus | 2.71E+03 | 8 | 0.0039 | **0.0099** |
| beta | Right-Amygdala | 9.37 | 8 | 0.0039 | **0.0099** |
| beta | lh-lateralorbitofrontal | 0.131 | 4 | 0.078 | 0.08 |
| beta | lh-rostralmiddlefrontal | 0.282 | 5 | 0.047 | **0.049** |
| beta | lh-inferiortemporal | 0.675 | 7 | 0.0078 | **0.013** |
| beta | lh-middletemporal | 1.97 | 6 | 0.039 | **0.042** |
| beta | Left-Hippocampus | 18.2 | 6 | 0.016 | **0.018** |
| #patients IED>no IED: number of patients for which the value in the ROI was higher in the scalp negative IED than the no IED condition. ROI names correspond to the ones of the Desikan atlas.  lh: left hemisphere, rh: right hemisphere, ROI: region of interest. | | | | | |

| Supplementary Table 5: hdEEG(ROI) ITC change between scalp-negative IEDs vs no IED | | | | | |
| --- | --- | --- | --- | --- | --- |
| Frequency band | **ROI name** | **Median difference** | **#patients IED>no IED** | **P‑value** | **Adjusted P‑value** |
| delta | rh-lateralorbitofrontal | 0.0213 | 5 | 0.37 | 0.63 |
| delta | rh-parsorbitalis | 0.00855 | 6 | 0.1 | 0.5 |
| delta | rh-frontalpole | 0.0138 | 6 | 0.37 | 0.63 |
| delta | rh-medialorbitofrontal | 0.0116 | 6 | 0.18 | 0.52 |
| delta | rh-parstriangularis | 0.0021 | 5 | 0.18 | 0.52 |
| delta | rh-parsopercularis | 7.42E-03 | 6 | 0.15 | 0.52 |
| delta | rh-rostralmiddlefrontal | 0.00118 | 5 | 0.63 | 0.77 |
| delta | rh-superiorfrontal | 0.0106 | 6 | 0.15 | 0.52 |
| delta | rh-caudalmiddlefrontal | -0.00344 | 4 | 0.46 | 0.69 |
| delta | rh-precentral | -0.000272 | 4 | 0.59 | 0.74 |
| delta | rh-paracentral | 0.0118 | 5 | 0.29 | 0.55 |
| delta | rh-rostralanteriorcingulate | 0.0126 | 5 | 0.25 | 0.54 |
| delta | rh-caudalanteriorcingulate | 0.0143 | 8 | 0.049 | 0.37 |
| delta | rh-posteriorcingulate | 0.00589 | 5 | 0.21 | 0.54 |
| delta | rh-isthmuscingulate | 0.012 | 8 | 0.037 | 0.37 |
| delta | rh-postcentral | -0.00331 | 4 | 0.5 | 0.71 |
| delta | rh-supramarginal | 0.0136 | 7 | 0.1 | 0.5 |
| delta | rh-superiorparietal | 1.21E-02 | 6 | 0.15 | 0.52 |
| delta | rh-inferiorparietal | 0.0197 | 8 | 0.049 | 0.37 |
| delta | rh-precuneus | 0.0116 | 7 | 0.049 | 0.37 |
| delta | rh-cuneus | -0.0179 | 3 | 0.79 | 0.87 |
| delta | rh-pericalcarine | 0.00696 | 5 | 0.37 | 0.63 |
| delta | rh-lateraloccipital | 0.0155 | 6 | 0.25 | 0.54 |
| delta | rh-lingual | 0.0233 | 6 | 0.13 | 0.52 |
| delta | rh-fusiform | 0.0214 | 7 | 0.027 | 0.37 |
| delta | rh-parahippocampal | 0.0234 | 7 | 0.014 | 0.37 |
| delta | rh-entorhinal | 0.0366 | 8 | 0.0098 | 0.37 |
| delta | rh-temporalpole | 0.0168 | 7 | 0.02 | 0.37 |
| delta | rh-inferiortemporal | 0.0267 | 6 | 0.15 | 0.52 |
| delta | rh-middletemporal | -1.70E-02 | 4 | 0.37 | 0.63 |
| delta | rh-bankssts | 0.0158 | 6 | 0.064 | 0.38 |
| delta | rh-superiortemporal | -0.0108 | 4 | 0.21 | 0.54 |
| delta | rh-transversetemporal | 0.0139 | 6 | 0.15 | 0.52 |
| delta | rh-insula | 0.0166 | 7 | 0.02 | 0.37 |
| delta | Right-Hippocampus | 0.0319 | 7 | 0.02 | 0.37 |
| delta | Right-Amygdala | 0.0263 | 7 | 0.014 | 0.37 |
| delta | lh-lateralorbitofrontal | 0.00274 | 5 | 0.46 | 0.69 |
| delta | lh-parsorbitalis | -0.0108 | 3 | 0.85 | 0.91 |
| delta | lh-frontalpole | 0.0082 | 5 | 0.25 | 0.54 |
| delta | lh-medialorbitofrontal | 0.00774 | 6 | 0.049 | 0.37 |
| delta | lh-parstriangularis | 0.0108 | 5 | 0.41 | 0.66 |
| delta | lh-parsopercularis | 4.61E-03 | 5 | 0.29 | 0.55 |
| delta | lh-rostralmiddlefrontal | 0.0216 | 7 | 0.037 | 0.37 |
| delta | lh-superiorfrontal | -0.00187 | 4 | 0.67 | 0.79 |
| delta | lh-caudalmiddlefrontal | 0.0136 | 5 | 0.18 | 0.52 |
| delta | lh-precentral | -0.000496 | 3 | 0.54 | 0.71 |
| delta | lh-paracentral | 0.00209 | 5 | 0.25 | 0.54 |
| delta | lh-rostralanteriorcingulate | 0.0114 | 6 | 0.049 | 0.37 |
| delta | lh-caudalanteriorcingulate | 0.00257 | 5 | 0.33 | 0.59 |
| delta | lh-posteriorcingulate | -0.000277 | 4 | 0.33 | 0.59 |
| delta | lh-isthmuscingulate | 0.00424 | 6 | 0.25 | 0.54 |
| delta | lh-postcentral | -0.00113 | 4 | 0.59 | 0.74 |
| delta | lh-supramarginal | -0.0204 | 3 | 0.75 | 0.86 |
| delta | lh-superiorparietal | 0.00559 | 6 | 0.59 | 0.74 |
| delta | lh-inferiorparietal | -0.00398 | 3 | 0.71 | 0.82 |
| delta | lh-precuneus | 0.000421 | 5 | 0.54 | 0.71 |
| delta | lh-cuneus | -0.00756 | 4 | 0.85 | 0.91 |
| delta | lh-pericalcarine | -0.00659 | 3 | 0.75 | 0.86 |
| delta | lh-lateraloccipital | 0.00498 | 6 | 0.15 | 0.52 |
| delta | lh-lingual | 0.00294 | 5 | 0.18 | 0.52 |
| delta | lh-fusiform | 0.0301 | 6 | 0.15 | 0.52 |
| delta | lh-parahippocampal | 0.00841 | 5 | 0.37 | 0.63 |
| delta | lh-entorhinal | 0.00624 | 7 | 0.15 | 0.52 |
| delta | lh-temporalpole | 0.00894 | 5 | 0.54 | 0.71 |
| delta | lh-inferiortemporal | -0.00889 | 4 | 0.92 | 0.94 |
| delta | lh-middletemporal | -0.0155 | 1 | 0.99 | 0.99 |
| delta | lh-bankssts | -0.0139 | 2 | 0.9 | 0.94 |
| delta | lh-superiortemporal | -0.0179 | 2 | 0.97 | 0.98 |
| delta | lh-transversetemporal | -0.00322 | 3 | 0.82 | 0.89 |
| delta | lh-insula | 0.000927 | 5 | 0.67 | 0.79 |
| delta | Left-Hippocampus | 0.00372 | 5 | 0.29 | 0.55 |
| delta | Left-Amygdala | 0.00264 | 5 | 0.63 | 0.77 |
| theta | rh-lateralorbitofrontal | 0.0248 | 7 | 0.1 | 0.5 |
| theta | rh-parsorbitalis | -0.00184 | 4 | 0.46 | 0.69 |
| theta | rh-frontalpole | 0.00287 | 5 | 0.25 | 0.54 |
| theta | rh-medialorbitofrontal | 0.0222 | 7 | 0.049 | 0.37 |
| theta | rh-parstriangularis | 0.0126 | 5 | 0.54 | 0.71 |
| theta | rh-parsopercularis | 0.0209 | 5 | 0.21 | 0.54 |
| theta | rh-rostralmiddlefrontal | -0.0012 | 4 | 0.46 | 0.69 |
| theta | rh-superiorfrontal | -0.0258 | 2 | 0.85 | 0.91 |
| theta | rh-caudalmiddlefrontal | 0.0277 | 6 | 0.064 | 0.38 |
| theta | rh-precentral | 0.0116 | 6 | 0.18 | 0.52 |
| theta | rh-paracentral | -0.0258 | 2 | 0.97 | 0.98 |
| theta | rh-rostralanteriorcingulate | 0.0288 | 5 | 0.21 | 0.54 |
| theta | rh-caudalanteriorcingulate | 0.00919 | 6 | 0.21 | 0.54 |
| theta | rh-posteriorcingulate | 0.021 | 6 | 0.082 | 0.44 |
| theta | rh-isthmuscingulate | 0.0303 | 5 | 0.15 | 0.52 |
| theta | rh-postcentral | 0.0127 | 5 | 0.18 | 0.52 |
| theta | rh-supramarginal | 0.00906 | 5 | 0.29 | 0.55 |
| theta | rh-superiorparietal | 0.0133 | 5 | 0.5 | 0.71 |
| theta | rh-inferiorparietal | 0.0178 | 5 | 0.21 | 0.54 |
| theta | rh-precuneus | 0.0388 | 6 | 0.29 | 0.55 |
| theta | rh-cuneus | 0.00673 | 6 | 0.18 | 0.52 |
| theta | rh-pericalcarine | 0.0225 | 7 | 0.082 | 0.44 |
| theta | rh-lateraloccipital | 0.0216 | 6 | 0.15 | 0.52 |
| theta | rh-lingual | 0.0383 | 6 | 0.049 | 0.37 |
| theta | rh-fusiform | 0.00322 | 5 | 0.25 | 0.54 |
| theta | rh-parahippocampal | 0.0219 | 5 | 0.15 | 0.52 |
| theta | rh-entorhinal | 0.0307 | 6 | 0.18 | 0.52 |
| theta | rh-temporalpole | 0.0344 | 5 | 0.33 | 0.59 |
| theta | rh-inferiortemporal | -0.00363 | 4 | 0.54 | 0.71 |
| theta | rh-middletemporal | -0.00398 | 4 | 0.71 | 0.82 |
| theta | rh-bankssts | -0.0257 | 4 | 0.54 | 0.71 |
| theta | rh-superiortemporal | 0.0421 | 6 | 0.13 | 0.52 |
| theta | rh-transversetemporal | 0.0229 | 7 | 0.027 | 0.37 |
| theta | rh-insula | 0.0568 | 7 | 0.037 | 0.37 |
| theta | Right-Hippocampus | 0.0373 | 6 | 0.13 | 0.52 |
| theta | Right-Amygdala | 0.0394 | 7 | 0.082 | 0.44 |
| theta | lh-lateralorbitofrontal | 0.0188 | 6 | 0.049 | 0.37 |
| theta | lh-parsorbitalis | 0.0244 | 7 | 0.064 | 0.38 |
| theta | lh-frontalpole | -0.00763 | 4 | 0.54 | 0.71 |
| theta | lh-medialorbitofrontal | 0.0149 | 5 | 0.29 | 0.55 |
| theta | lh-parstriangularis | 0.0286 | 7 | 0.15 | 0.52 |
| theta | lh-parsopercularis | -0.0186 | 4 | 0.71 | 0.82 |
| theta | lh-rostralmiddlefrontal | 0.018 | 6 | 0.25 | 0.54 |
| theta | lh-superiorfrontal | 0.0138 | 6 | 0.25 | 0.54 |
| theta | lh-caudalmiddlefrontal | 0.00758 | 6 | 0.41 | 0.66 |
| theta | lh-precentral | -0.00248 | 3 | 0.75 | 0.86 |
| theta | lh-paracentral | -0.0221 | 4 | 0.9 | 0.94 |
| theta | lh-rostralanteriorcingulate | 0.0151 | 5 | 0.5 | 0.71 |
| theta | lh-caudalanteriorcingulate | 0.00904 | 5 | 0.54 | 0.71 |
| theta | lh-posteriorcingulate | 0.0338 | 6 | 0.18 | 0.52 |
| theta | lh-isthmuscingulate | -0.00422 | 4 | 0.46 | 0.69 |
| theta | lh-postcentral | 0.0156 | 5 | 0.33 | 0.59 |
| theta | lh-supramarginal | 0.00818 | 5 | 0.46 | 0.69 |
| theta | lh-superiorparietal | 0.0208 | 6 | 0.54 | 0.71 |
| theta | lh-inferiorparietal | -0.000515 | 4 | 0.59 | 0.74 |
| theta | lh-precuneus | 0.0287 | 7 | 0.21 | 0.54 |
| theta | lh-cuneus | -0.016 | 3 | 0.94 | 0.95 |
| theta | lh-pericalcarine | 0.0184 | 5 | 0.33 | 0.59 |
| theta | lh-lateraloccipital | -0.00369 | 4 | 0.54 | 0.71 |
| theta | lh-lingual | 0.0424 | 6 | 0.29 | 0.55 |
| theta | lh-fusiform | 0.00863 | 6 | 0.37 | 0.63 |
| theta | lh-parahippocampal | 0.00386 | 5 | 0.46 | 0.69 |
| theta | lh-entorhinal | 2.16E-05 | 5 | 0.67 | 0.79 |
| theta | lh-temporalpole | 0.000996 | 5 | 0.54 | 0.71 |
| theta | lh-inferiortemporal | 0.00489 | 5 | 0.25 | 0.54 |
| theta | lh-middletemporal | -0.0131 | 4 | 0.54 | 0.71 |
| theta | lh-bankssts | -0.00652 | 4 | 0.54 | 0.71 |
| theta | lh-superiortemporal | -0.0128 | 3 | 0.94 | 0.95 |
| theta | lh-transversetemporal | -0.000551 | 4 | 0.5 | 0.71 |
| theta | lh-insula | 0.0155 | 7 | 0.15 | 0.52 |
| theta | Left-Hippocampus | -0.0172 | 3 | 0.88 | 0.92 |
| theta | Left-Amygdala | -0.0116 | 4 | 0.54 | 0.71 |
| alpha | rh-lateralorbitofrontal | 0.0252 | 6 | 0.21 | 0.54 |
| alpha | rh-parsorbitalis | -0.0446 | 2 | 0.97 | 0.98 |
| alpha | rh-frontalpole | 0.00635 | 6 | 0.15 | 0.52 |
| alpha | rh-medialorbitofrontal | 0.0494 | 7 | 0.0098 | 0.37 |
| alpha | rh-parstriangularis | 0.0472 | 7 | 0.15 | 0.52 |
| alpha | rh-parsopercularis | 0.0329 | 9 | 0.002 | 0.37 |
| alpha | rh-rostralmiddlefrontal | 0.0484 | 7 | 0.027 | 0.37 |
| alpha | rh-superiorfrontal | 0.00903 | 6 | 0.29 | 0.55 |
| alpha | rh-caudalmiddlefrontal | 0.0027 | 5 | 0.5 | 0.71 |
| alpha | rh-precentral | 0.00324 | 5 | 0.5 | 0.71 |
| alpha | rh-paracentral | -0.0125 | 3 | 0.85 | 0.91 |
| alpha | rh-rostralanteriorcingulate | 0.0647 | 8 | 0.064 | 0.38 |
| alpha | rh-caudalanteriorcingulate | -0.0123 | 4 | 0.41 | 0.66 |
| alpha | rh-posteriorcingulate | -0.00177 | 4 | 0.29 | 0.55 |
| alpha | rh-isthmuscingulate | 0.0238 | 6 | 0.18 | 0.52 |
| alpha | rh-postcentral | 0.0145 | 6 | 0.064 | 0.38 |
| alpha | rh-supramarginal | 0.00417 | 5 | 0.54 | 0.71 |
| alpha | rh-superiorparietal | 0.00917 | 5 | 0.37 | 0.63 |
| alpha | rh-inferiorparietal | 0.0238 | 6 | 0.5 | 0.71 |
| alpha | rh-precuneus | 0.00052 | 5 | 0.37 | 0.63 |
| alpha | rh-cuneus | 0.0454 | 7 | 0.037 | 0.37 |
| alpha | rh-pericalcarine | 0.002 | 5 | 0.5 | 0.71 |
| alpha | rh-lateraloccipital | 0.00102 | 5 | 0.63 | 0.77 |
| alpha | rh-lingual | 0.0123 | 6 | 0.25 | 0.54 |
| alpha | rh-fusiform | 0.000306 | 5 | 0.41 | 0.66 |
| alpha | rh-parahippocampal | 0.00168 | 5 | 0.37 | 0.63 |
| alpha | rh-entorhinal | 0.0131 | 5 | 0.41 | 0.66 |
| alpha | rh-temporalpole | -0.00205 | 4 | 0.82 | 0.89 |
| alpha | rh-inferiortemporal | -0.00532 | 4 | 0.54 | 0.71 |
| alpha | rh-middletemporal | 0.022 | 7 | 0.037 | 0.37 |
| alpha | rh-bankssts | -0.00545 | 4 | 0.5 | 0.71 |
| alpha | rh-superiortemporal | 0.0389 | 7 | 0.037 | 0.37 |
| alpha | rh-transversetemporal | 0.0187 | 6 | 0.21 | 0.54 |
| alpha | rh-insula | 0.00588 | 5 | 0.25 | 0.54 |
| alpha | Right-Hippocampus | 0.0025 | 5 | 0.33 | 0.59 |
| alpha | Right-Amygdala | 0.0237 | 6 | 0.25 | 0.54 |
| alpha | lh-lateralorbitofrontal | 0.00328 | 5 | 0.54 | 0.71 |
| alpha | lh-parsorbitalis | -0.027 | 3 | 0.88 | 0.92 |
| alpha | lh-frontalpole | -0.0275 | 4 | 0.92 | 0.94 |
| alpha | lh-medialorbitofrontal | 0.0377 | 6 | 0.082 | 0.44 |
| alpha | lh-parstriangularis | -0.00356 | 4 | 0.33 | 0.59 |
| alpha | lh-parsopercularis | -0.0155 | 3 | 0.82 | 0.89 |
| alpha | lh-rostralmiddlefrontal | -0.0303 | 3 | 0.88 | 0.92 |
| alpha | lh-superiorfrontal | -0.00549 | 4 | 0.71 | 0.82 |
| alpha | lh-caudalmiddlefrontal | 0.00312 | 5 | 0.63 | 0.77 |
| alpha | lh-precentral | -0.0321 | 2 | 0.9 | 0.94 |
| alpha | lh-paracentral | -0.00371 | 4 | 0.59 | 0.74 |
| alpha | lh-rostralanteriorcingulate | 0.0348 | 6 | 0.049 | 0.37 |
| alpha | lh-caudalanteriorcingulate | -0.00163 | 4 | 0.46 | 0.69 |
| alpha | lh-posteriorcingulate | 0.00242 | 5 | 0.37 | 0.63 |
| alpha | lh-isthmuscingulate | 0.0306 | 8 | 0.037 | 0.37 |
| alpha | lh-postcentral | 0.0108 | 5 | 0.67 | 0.79 |
| alpha | lh-supramarginal | -0.0639 | 3 | 0.94 | 0.95 |
| alpha | lh-superiorparietal | -0.0169 | 4 | 0.79 | 0.87 |
| alpha | lh-inferiorparietal | -0.0187 | 4 | 0.79 | 0.87 |
| alpha | lh-precuneus | 0.0192 | 5 | 0.46 | 0.69 |
| alpha | lh-cuneus | 0.045 | 8 | 0.014 | 0.37 |
| alpha | lh-pericalcarine | 0.0529 | 8 | 0.0039 | 0.37 |
| alpha | lh-lateraloccipital | 0.011 | 8 | 0.037 | 0.37 |
| alpha | lh-lingual | 0.065 | 7 | 0.014 | 0.37 |
| alpha | lh-fusiform | 0.0327 | 6 | 0.064 | 0.38 |
| alpha | lh-parahippocampal | 0.0125 | 5 | 0.15 | 0.52 |
| alpha | lh-entorhinal | 0.0045 | 5 | 0.25 | 0.54 |
| alpha | lh-temporalpole | 0.0158 | 5 | 0.25 | 0.54 |
| alpha | lh-inferiortemporal | 0.0233 | 5 | 0.29 | 0.55 |
| alpha | lh-middletemporal | -0.00763 | 4 | 0.63 | 0.77 |
| alpha | lh-bankssts | 0.00227 | 6 | 0.13 | 0.52 |
| alpha | lh-superiortemporal | 0.0513 | 6 | 0.13 | 0.52 |
| alpha | lh-transversetemporal | 0.00683 | 5 | 0.21 | 0.54 |
| alpha | lh-insula | 0.0313 | 8 | 0.0059 | 0.37 |
| alpha | Left-Hippocampus | 0.04 | 8 | 0.027 | 0.37 |
| alpha | Left-Amygdala | 0.0513 | 6 | 0.1 | 0.5 |
| beta | rh-lateralorbitofrontal | -0.0105 | 4 | 0.63 | 0.77 |
| beta | rh-parsorbitalis | -3.90E-06 | 4 | 0.41 | 0.66 |
| beta | rh-frontalpole | 0.0294 | 6 | 0.027 | 0.37 |
| beta | rh-medialorbitofrontal | 0.022 | 6 | 0.15 | 0.52 |
| beta | rh-parstriangularis | -0.0179 | 3 | 0.79 | 0.87 |
| beta | rh-parsopercularis | 0.00681 | 5 | 0.25 | 0.54 |
| beta | rh-rostralmiddlefrontal | 0.0158 | 7 | 0.064 | 0.38 |
| beta | rh-superiorfrontal | 0.00141 | 5 | 0.59 | 0.74 |
| beta | rh-caudalmiddlefrontal | -0.00424 | 4 | 0.67 | 0.79 |
| beta | rh-precentral | 0.0149 | 7 | 0.049 | 0.37 |
| beta | rh-paracentral | -0.0254 | 4 | 0.92 | 0.94 |
| beta | rh-rostralanteriorcingulate | 0.00868 | 5 | 0.18 | 0.52 |
| beta | rh-caudalanteriorcingulate | 0.0031 | 5 | 0.29 | 0.55 |
| beta | rh-posteriorcingulate | 0.0003 | 5 | 0.5 | 0.71 |
| beta | rh-isthmuscingulate | -0.0187 | 3 | 0.92 | 0.94 |
| beta | rh-postcentral | 0.0152 | 6 | 0.29 | 0.55 |
| beta | rh-supramarginal | 0.00259 | 5 | 0.63 | 0.77 |
| beta | rh-superiorparietal | -0.0168 | 3 | 0.79 | 0.87 |
| beta | rh-inferiorparietal | -0.0104 | 3 | 0.79 | 0.87 |
| beta | rh-precuneus | -0.00804 | 4 | 0.82 | 0.89 |
| beta | rh-cuneus | 0.0158 | 6 | 0.082 | 0.44 |
| beta | rh-pericalcarine | 0.0155 | 7 | 0.064 | 0.38 |
| beta | rh-lateraloccipital | 0.00209 | 6 | 0.18 | 0.52 |
| beta | rh-lingual | 0.023 | 7 | 0.049 | 0.37 |
| beta | rh-fusiform | -0.000897 | 4 | 0.54 | 0.71 |
| beta | rh-parahippocampal | -1.94E-05 | 4 | 0.25 | 0.54 |
| beta | rh-entorhinal | 0.0219 | 7 | 0.049 | 0.37 |
| beta | rh-temporalpole | 0.0033 | 6 | 0.21 | 0.54 |
| beta | rh-inferiortemporal | -0.0135 | 4 | 0.41 | 0.66 |
| beta | rh-middletemporal | -0.00559 | 3 | 0.54 | 0.71 |
| beta | rh-bankssts | -0.00402 | 4 | 0.63 | 0.77 |
| beta | rh-superiortemporal | 0.00625 | 5 | 0.18 | 0.52 |
| beta | rh-transversetemporal | 0.0128 | 5 | 0.29 | 0.55 |
| beta | rh-insula | 0.0203 | 5 | 0.15 | 0.52 |
| beta | Right-Hippocampus | 0.00496 | 6 | 0.15 | 0.52 |
| beta | Right-Amygdala | 0.0148 | 6 | 0.064 | 0.38 |
| beta | lh-lateralorbitofrontal | 0.0108 | 6 | 0.33 | 0.59 |
| beta | lh-parsorbitalis | 0.00276 | 5 | 0.37 | 0.63 |
| beta | lh-frontalpole | 0.00683 | 5 | 0.54 | 0.71 |
| beta | lh-medialorbitofrontal | 0.0422 | 6 | 0.18 | 0.52 |
| beta | lh-parstriangularis | -0.00148 | 4 | 0.59 | 0.74 |
| beta | lh-parsopercularis | 0.000322 | 5 | 0.54 | 0.71 |
| beta | lh-rostralmiddlefrontal | 0.00457 | 5 | 0.25 | 0.54 |
| beta | lh-superiorfrontal | -0.0118 | 4 | 0.67 | 0.79 |
| beta | lh-caudalmiddlefrontal | -0.00624 | 3 | 0.67 | 0.79 |
| beta | lh-precentral | 0.0116 | 6 | 0.064 | 0.38 |
| beta | lh-paracentral | 0.00572 | 5 | 0.67 | 0.79 |
| beta | lh-rostralanteriorcingulate | 0.0493 | 8 | 0.064 | 0.38 |
| beta | lh-caudalanteriorcingulate | 0.00309 | 5 | 0.25 | 0.54 |
| beta | lh-posteriorcingulate | -0.0117 | 3 | 0.82 | 0.89 |
| beta | lh-isthmuscingulate | 0.0114 | 6 | 0.18 | 0.52 |
| beta | lh-postcentral | 0.0138 | 8 | 0.0059 | 0.37 |
| beta | lh-supramarginal | -0.00381 | 4 | 0.5 | 0.71 |
| beta | lh-superiorparietal | 0.00502 | 5 | 0.25 | 0.54 |
| beta | lh-inferiorparietal | -0.00464 | 4 | 0.33 | 0.59 |
| beta | lh-precuneus | 0.000987 | 5 | 0.41 | 0.66 |
| beta | lh-cuneus | 0.0168 | 6 | 0.46 | 0.69 |
| beta | lh-pericalcarine | 0.0293 | 6 | 0.25 | 0.54 |
| beta | lh-lateraloccipital | 0.0257 | 7 | 0.21 | 0.54 |
| beta | lh-lingual | 0.0181 | 7 | 0.1 | 0.5 |
| beta | lh-fusiform | 0.00194 | 5 | 0.29 | 0.55 |
| beta | lh-parahippocampal | 0.0154 | 6 | 0.18 | 0.52 |
| beta | lh-entorhinal | 0.00954 | 6 | 0.29 | 0.55 |
| beta | lh-temporalpole | 0.00449 | 5 | 0.63 | 0.77 |
| beta | lh-inferiortemporal | -0.0123 | 4 | 0.79 | 0.87 |
| beta | lh-middletemporal | -0.0128 | 2 | 0.96 | 0.98 |
| beta | lh-bankssts | 0.0097 | 6 | 0.41 | 0.66 |
| beta | lh-superiortemporal | -0.0241 | 3 | 0.79 | 0.87 |
| beta | lh-transversetemporal | 0.008 | 5 | 0.46 | 0.69 |
| beta | lh-insula | 0.00846 | 5 | 0.59 | 0.74 |
| beta | Left-Hippocampus | 0.0347 | 6 | 0.18 | 0.52 |
| beta | Left-Amygdala | 0.0134 | 6 | 0.29 | 0.55 |
| #patients IED>no IED: number of patients for which the value in the ROI was higher in the scalp negative IED than the no IED condition. ROI names correspond to the ones of the Desikan atlas.  lh: left hemisphere, rh: right hemisphere, ROI: region of interest. | | | | | |

| Supplementary Table 6: iEEG(ROI) ITC change between scalp-negative IEDs vs no IED | | | | | |
| --- | --- | --- | --- | --- | --- |
| Frequency band | **ROI name** | **Median difference** | **#patients IED>no IED** | **P‑value** | **Adjusted P‑value** |
| delta | rh-lateralorbitofrontal | 0.0209 | 7 | 0.0078 | **0.047** |
| delta | rh-rostralmiddlefrontal | 0.00585 | 5 | 0.18 | 0.32 |
| delta | rh-fusiform | 0.0759 | 5 | 0.039 | 0.099 |
| delta | rh-inferiortemporal | 0.0132 | 8 | 0.0039 | **0.047** |
| delta | rh-middletemporal | 0.0162 | 8 | 0.049 | 0.11 |
| delta | Right-Hippocampus | 1.38E-01 | 7 | 0.0078 | **0.047** |
| delta | Right-Amygdala | 0.101 | 8 | 0.0039 | **0.047** |
| delta | lh-lateralorbitofrontal | -0.000893 | 3 | 0.66 | 0.77 |
| delta | lh-rostralmiddlefrontal | -0.00696 | 2 | 0.84 | 0.92 |
| delta | lh-inferiortemporal | 0.00791 | 5 | 0.15 | 0.27 |
| delta | lh-middletemporal | 0.00359 | 4 | 0.53 | 0.65 |
| delta | Left-Hippocampus | 3.06E-05 | 3 | 0.5 | 0.63 |
| theta | rh-lateralorbitofrontal | 0.0261 | 7 | 0.012 | 0.051 |
| theta | rh-rostralmiddlefrontal | 0.0143 | 7 | 0.29 | 0.47 |
| theta | rh-fusiform | 0.0526 | 7 | 0.0078 | **0.047** |
| theta | rh-inferiortemporal | 0.029 | 6 | 0.027 | 0.077 |
| theta | rh-middletemporal | 0.0224 | 7 | 0.027 | 0.077 |
| theta | Right-Hippocampus | 1.40E-01 | 7 | 0.0078 | **0.047** |
| theta | Right-Amygdala | 0.0795 | 7 | 0.012 | 0.051 |
| theta | lh-lateralorbitofrontal | 0.00261 | 3 | 0.34 | 0.5 |
| theta | lh-rostralmiddlefrontal | -0.00393 | 3 | 0.42 | 0.58 |
| theta | lh-inferiortemporal | -0.0167 | 1 | 0.89 | 0.93 |
| theta | lh-middletemporal | 0.0102 | 5 | 0.11 | 0.21 |
| theta | Left-Hippocampus | -0.0348 | 1 | 0.98 | 0.98 |
| alpha | rh-lateralorbitofrontal | 0.0159 | 5 | 0.42 | 0.58 |
| alpha | rh-rostralmiddlefrontal | 0.00536 | 5 | 0.63 | 0.76 |
| alpha | rh-fusiform | 0.0566 | 6 | 0.016 | 0.058 |
| alpha | rh-inferiortemporal | 0.0351 | 6 | 0.064 | 0.14 |
| alpha | rh-middletemporal | 0.0061 | 7 | 0.037 | 0.099 |
| alpha | Right-Hippocampus | 1.28E-01 | 8 | 0.0039 | **0.047** |
| alpha | Right-Amygdala | 0.0687 | 7 | 0.012 | 0.051 |
| alpha | lh-lateralorbitofrontal | 0.00935 | 4 | 0.34 | 0.5 |
| alpha | lh-rostralmiddlefrontal | -0.00319 | 3 | 0.5 | 0.63 |
| alpha | lh-inferiortemporal | -0.0327 | 2 | 0.92 | 0.94 |
| alpha | lh-middletemporal | 0.00523 | 5 | 0.34 | 0.5 |
| alpha | Left-Hippocampus | 0.0178 | 5 | 0.078 | 0.16 |
| beta | rh-lateralorbitofrontal | 0.00479 | 4 | 0.32 | 0.5 |
| beta | rh-rostralmiddlefrontal | 0.0123 | 5 | 0.29 | 0.47 |
| beta | rh-fusiform | 0.0299 | 6 | 0.016 | 0.058 |
| beta | rh-inferiortemporal | 0.0276 | 6 | 0.049 | 0.11 |
| beta | rh-middletemporal | 0.0185 | 7 | 0.02 | 0.063 |
| beta | Right-Hippocampus | 1.07E-01 | 8 | 0.0039 | **0.047** |
| beta | Right-Amygdala | 0.0576 | 7 | 0.02 | 0.063 |
| beta | lh-lateralorbitofrontal | -0.0157 | 2 | 0.84 | 0.92 |
| beta | lh-rostralmiddlefrontal | -0.00175 | 3 | 0.5 | 0.63 |
| beta | lh-inferiortemporal | 0.0109 | 5 | 0.078 | 0.16 |
| beta | lh-middletemporal | -0.000502 | 3 | 0.71 | 0.81 |
| beta | Left-Hippocampus | -0.0106 | 1 | 0.89 | 0.93 |
| #patients IED>no IED: number of patients for which the value in the ROI was higher in the scalp negative IED than the no IED condition. ROI names correspond to the ones of the Desikan atlas.  lh: left hemisphere, rh: right hemisphere, ROI: region of interest. | | | | | |

| Supplementary Table 7: Whole brain integration and segregation changes between scalp-negative IEDs vs no IED | | | | | | |
| --- | --- | --- | --- | --- | --- | --- |
| Network Metric | **Modality** | **Frequency band** | **Median difference** | **#patients IED>no IED** | **P‑value** | **Adjusted P‑value** |
| Integration | hdEEG(ROI) | delta | 0.0354 | 9 | 0.002 | **0.0039** |
| Integration | hdEEG(ROI) | theta | 0.0151 | 9 | 0.002 | **0.0039** |
| Integration | hdEEG(ROI) | alpha | 0.0113 | 7 | 0.027 | **0.027** |
| Integration | hdEEG(ROI) | beta | 0.00634 | 8 | 0.0098 | **0.013** |
| Integration | iEEG(channel) | delta | 0.0214 | 7 | 0.02 | **0.026** |
| Integration | iEEG(channel) | theta | 0.0252 | 8 | 0.037 | **0.037** |
| Integration | iEEG(channel) | alpha | 0.0327 | 8 | 0.0098 | **0.02** |
| Integration | iEEG(channel) | beta | 0.0312 | 9 | 0.002 | **0.0078** |
| Integration | iEEG(ROI) | delta | 0.00185 | 6 | 0.037 | **0.037** |
| Integration | iEEG(ROI) | theta | 0.00108 | 8 | 0.0039 | **0.0078** |
| Integration | iEEG(ROI) | alpha | 0.00185 | 8 | 0.0059 | **0.0078** |
| Integration | iEEG(ROI) | beta | 0.00261 | 9 | 0.002 | **0.0078** |
| Segregation | hdEEG(ROI) | delta | 0.0338 | 9 | 0.002 | **0.0078** |
| Segregation | hdEEG(ROI) | theta | 0.0149 | 8 | 0.0039 | **0.0078** |
| Segregation | hdEEG(ROI) | alpha | 0.0114 | 8 | 0.0098 | **0.0098** |
| Segregation | hdEEG(ROI) | beta | 0.0064 | 8 | 0.0059 | **0.0078** |
| Segregation | iEEG(channel) | delta | 0.0203 | 8 | 0.0059 | **0.012** |
| Segregation | iEEG(channel) | theta | 0.023 | 8 | 0.037 | **0.037** |
| Segregation | iEEG(channel) | alpha | 0.0231 | 8 | 0.014 | **0.018** |
| Segregation | iEEG(channel) | beta | 0.0227 | 9 | 0.002 | **0.0078** |
| Segregation | iEEG(ROI) | delta | 0.00684 | 6 | 0.027 | **0.027** |
| Segregation | iEEG(ROI) | theta | 0.00424 | 8 | 0.014 | **0.018** |
| Segregation | iEEG(ROI) | alpha | 0.00597 | 8 | 0.014 | **0.018** |
| Segregation | iEEG(ROI) | beta | 0.00693 | 9 | 0.002 | **0.0078** |
| #patients IED>no IED: number of patients for which the value in the ROI was higher in the scalp negative IED than the no IED condition. ROI names correspond to the ones of the Desikan atlas.  ROI: region of interest. | | | | | | |

| **Epoch** | **Modalities** | **Metrics** | **R^2^** | **Fstat** | **P-value** |
| --- | --- | --- | --- | --- | --- |
| noIED | EEG | integration | 0.98 | 2.8 | 0.1 |
| noIED | EEG | segregation | 0.98 | 2.7 | 0.11 |
| scalpnegIED | EEG | integration | 0.73 | 1.8 | 0.19 |
| scalpnegIED | EEG | segregation | 0.77 | 2.3 | 0.14 |
| noIED | iEEG | integration | 0.72 | 0.8 | 0.38 |
| noIED | iEEG | segregation | 0.82 | 1.4 | 0.24 |
| scalpnegIED | iEEG | integration | 0.78 | 0.13 | 0.72 |
| scalpnegIED | iEEG | segregation | 0.9 | 0.43 | 0.52 |
| noIED | iEEG(ROI) | integration | 0.99 | 4.6 | *0.041* |
| noIED | iEEG(ROI) | segregation | 0.98 | 4 | 0.056 |
| scalpnegIED | iEEG(ROI) | integration | 0.99 | 3.3 | 0.08 |
| scalpnegIED | iEEG(ROI) | segregation | 0.98 | 2.3 | 0.14 |
| scalpnegIED-noIED | EEG | integration | 0.25 | 0.078 | 0.78 |
| scalpnegIED-noIED | EEG | segregation | 0.12 | 0.26 | 0.61 |
| scalpnegIED-noIED | iEEG | integration | 0.14 | 0.36 | 0.55 |
| scalpnegIED-noIED | iEEG | segregation | 0.23 | 0.37 | 0.55 |
| scalpnegIED-noIED | iEEG(ROI) | integration | 0.7 | 0.36 | 0.55 |
| scalpnegIED-noIED | iEEG(ROI) | segregation | 0.69 | 0.52 | 0.48 |

**Supplementary table 8:** Results of the regression of the size of the irritative zone and whole brain network measures depending on the condition, modality and metric types. P-values are uncorrected. The formula of the generalized random effect model is: Network measure ~ (IZ size)*(frequency band) + ( 1 | Patient ID). The model used a gamma distribution with a logit link function. R^2^: adjusted R-square.

| **Epoch** | **Modalities** | **Metrics** | **R^2^** | **F-stat** | **P-value** |
| --- | --- | --- | --- | --- | --- |
| noIED | EEG | integration | 0.98 | 4.1 | 0.054 |
| noIED | EEG | segregation | 0.98 | 4.1 | 0.053 |
| scalpnegIED | EEG | integration | 0.74 | 2.1 | 0.16 |
| scalpnegIED | EEG | segregation | 0.77 | 2.3 | 0.14 |
| noIED | iEEG | integration | 0.81 | 0.082 | 0.78 |
| noIED | iEEG | segregation | 0.89 | 0.41 | 0.53 |
| scalpnegIED | iEEG | integration | 0.78 | 0.063 | 0.8 |
| scalpnegIED | iEEG | segregation | 0.9 | 0.91 | 0.35 |
| noIED | iEEG(ROI) | integration | 0.99 | 4.5 | *0.043* |
| noIED | iEEG(ROI) | segregation | 0.98 | 3.7 | 0.065 |
| scalpnegIED | iEEG(ROI) | integration | 0.99 | 4.8 | *0.038* |
| scalpnegIED | iEEG(ROI) | segregation | 0.98 | 3.9 | 0.059 |
| scalpnegIED-noIED | EEG | integration | 0.22 | 0.015 | 0.9 |
| scalpnegIED-noIED | EEG | segregation | 0.1 | 0.018 | 0.9 |
| scalpnegIED-noIED | iEEG | integration | 0.34 | 0.54 | 0.47 |
| scalpnegIED-noIED | iEEG | segregation | 0.39 | 0.46 | 0.5 |
| scalpnegIED-noIED | iEEG(ROI) | integration | 0.66 | 0.36 | 0.55 |
| scalpnegIED-noIED | iEEG(ROI) | segregation | 0.68 | 0.32 | 0.58 |

**Supplementary table 9:** Results of the regression of the neuropsychological score on verbal episodic memory and whole brain network measures depending on the condition, modality and metric types. P-values are uncorrected. The formula of the generalized linear model with random intercept for the patients: Network measure ~ (score)*(frequency band) + ( 1 | Patient ID). The model used a gamma distribution with a logit link function. R^2^: adjusted R-square.

| **Epoch** | **Modalities** | **Metrics** | **R^2^** | **F-stat** | **P-value** |
| --- | --- | --- | --- | --- | --- |
| noIED | EEG | integration | 0.97 | 0.17 | 0.68 |
| noIED | EEG | segregation | 0.97 | 0.19 | 0.67 |
| scalpnegIED | EEG | integration | 0.71 | 1.3 | 0.26 |
| scalpnegIED | EEG | segregation | 0.75 | 1.5 | 0.23 |
| noIED | iEEG | integration | 0.67 | 0.19 | 0.66 |
| noIED | iEEG | segregation | 0.79 | 0.002 | 0.96 |
| scalpnegIED | iEEG | integration | 0.75 | 0.4 | 0.53 |
| scalpnegIED | iEEG | segregation | 0.88 | 0.063 | 0.8 |
| noIED | iEEG(ROI) | integration | 0.99 | 0.82 | 0.37 |
| noIED | iEEG(ROI) | segregation | 0.97 | 0.58 | 0.45 |
| scalpnegIED | iEEG(ROI) | integration | 0.99 | 0.63 | 0.43 |
| scalpnegIED | iEEG(ROI) | segregation | 0.98 | 0.35 | 0.56 |
| scalpnegIED-noIED | EEG | integration | 0.29 | 3.6 | 0.07 |
| scalpnegIED-noIED | EEG | segregation | 0.2 | 4.6 | *0.042* |
| scalpnegIED-noIED | iEEG | integration | 0.21 | 0.043 | 0.84 |
| scalpnegIED-noIED | iEEG | segregation | 0.26 | 0.07 | 0.79 |
| scalpnegIED-noIED | iEEG(ROI) | integration | 0.67 | 0.034 | 0.85 |
| scalpnegIED-noIED | iEEG(ROI) | segregation | 0.68 | 0.026 | 0.87 |

**Supplementary table 10**: Results of the regression of the neuropsychological score on non-verbal episodic memory and whole brain network measures depending on the condition, modality and metric types. P-values are uncorrected. The formula of the generalized linear model with random intercept for the patients is: Network measure ~ (score)*(frequency band) + ( 1 | Patient ID). The model used a gamma distribution with a logit link function. R^2^: adjusted R-square.

| **Name** | **Estimate** | **SE** | **t-value** | **DF** | **P-value** |
| --- | --- | --- | --- | --- | --- |
| Intercept | 2.7 | 0.9 | 3 | 13 | **0.0093** |
| Test | 2 | 1.7 | 1.1 | 13 | 0.27 |
| IZ_size | -0.25 | 0.058 | -4.3 | 13 | **0.00085** |
| Interaction | -0.1 | 0.1 | -1 | 13 | 0.34 |

**Supplementary table 11**: Summary of the generalized linear mixed model assessing the influence of test type (verbal vs. non-verbal episodic memory) and IZ size on neuropsychological scores. The model was defined as Score ~ (Test type) × (IZ size) + (1 | Patient ID), fitted with a Gamma distribution and logit link. Scores were normalized by dividing by 15 (maximum possible score) to fit within the (0, 1) interval required for the logit transformation. Estimate: estimate of the regression coefficient, SE: standard error, DF: degree of freedom.


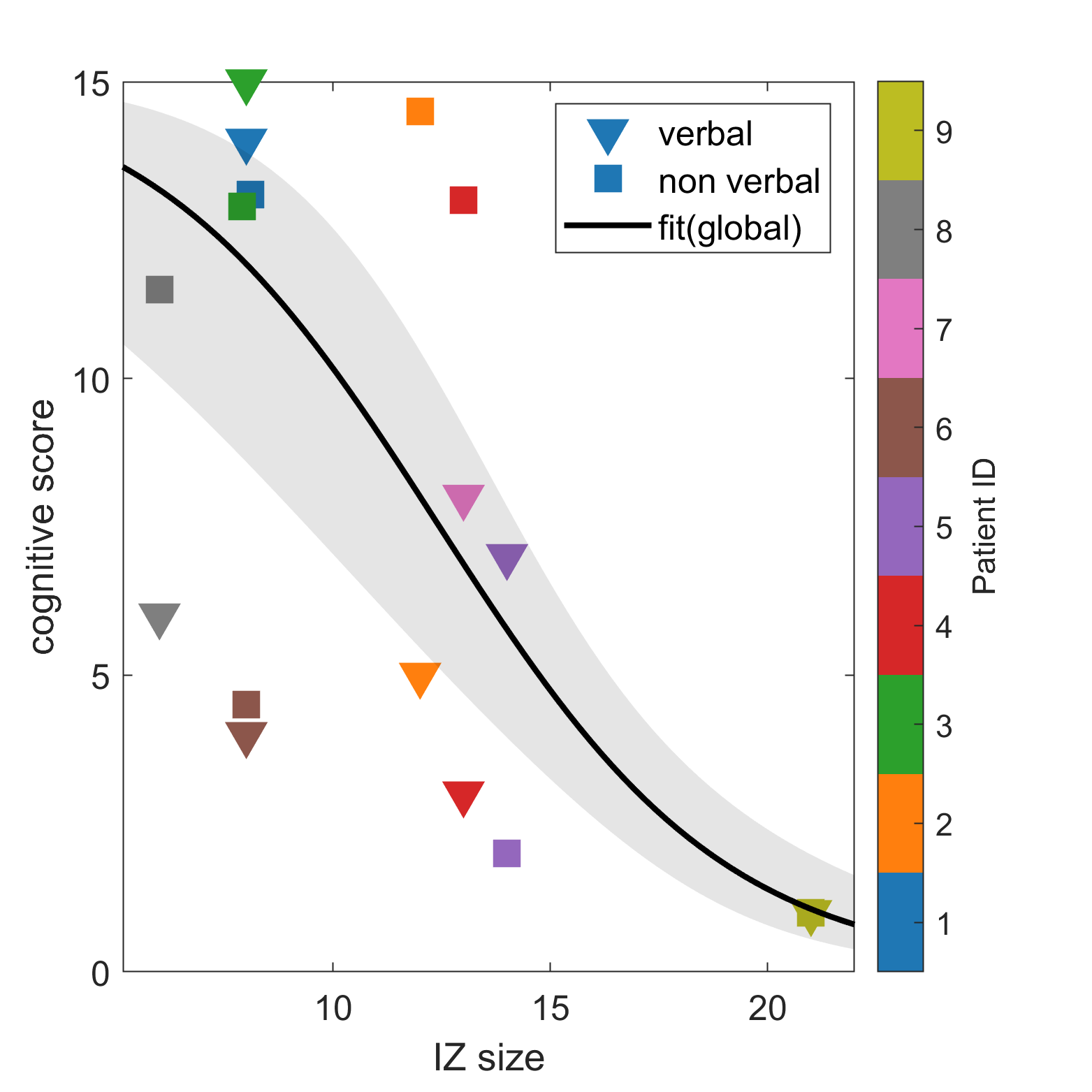


**Figure S2**: Scatter plot showing the relationship between neuropsychological test scores and IZ size. Verbal episodic memory scores (triangles) and non-verbal scores (squares) are color-coded by patient. The fitted generalized linear mixed model (Score ~ (Test type) × (IZ size) + (1 | Patient ID)) used a Gamma distribution with a logit link; scores were divided by 15 to fit the (0, 1) range and rescaled for plotting. IZ size was significantly negatively correlated with performance across both test types (p<0.001, Supp. Table 11). Non-verbal scores for patients 1 and 3 were slightly offset to prevent overlap.

| Supplementary Table 12: hdEEG(ROI) integration changes between scalp-negative IEDs vs no IED | | | | | |
| --- | --- | --- | --- | --- | --- |
| Frequency band | **ROI name** | **Median difference** | **#patients IED>no IED** | **P-value** | **Adjusted P‑value** |
| delta | rh-lateralorbitofrontal | 0.0376 | 9 | 0.002 | **0.017** |
| delta | rh-parsorbitalis | 0.0454 | 9 | 0.002 | **0.017** |
| delta | rh-frontalpole | 0.0529 | 9 | 0.002 | **0.017** |
| delta | rh-medialorbitofrontal | 0.0414 | 9 | 0.002 | **0.017** |
| delta | rh-parstriangularis | 0.0324 | 8 | 0.0098 | **0.028** |
| delta | rh-parsopercularis | 0.0468 | 9 | 0.002 | **0.017** |
| delta | rh-rostralmiddlefrontal | 0.0505 | 8 | 0.0039 | **0.017** |
| delta | rh-superiorfrontal | 0.0471 | 9 | 0.002 | **0.017** |
| delta | rh-caudalmiddlefrontal | 0.036 | 9 | 0.002 | **0.017** |
| delta | rh-precentral | 0.0169 | 7 | 0.02 | **0.041** |
| delta | rh-paracentral | 0.0119 | 8 | 0.02 | **0.041** |
| delta | rh-rostralanteriorcingulate | 0.0563 | 9 | 0.002 | **0.017** |
| delta | rh-caudalanteriorcingulate | 0.0572 | 9 | 0.002 | **0.017** |
| delta | rh-posteriorcingulate | 0.034 | 8 | 0.0059 | **0.02** |
| delta | rh-isthmuscingulate | 0.0404 | 8 | 0.014 | **0.034** |
| delta | rh-postcentral | 0.0155 | 7 | 0.027 | 0.052 |
| delta | rh-supramarginal | 0.0228 | 8 | 0.0059 | **0.02** |
| delta | rh-superiorparietal | 0.00559 | 8 | 0.0059 | **0.02** |
| delta | rh-inferiorparietal | 0.0397 | 9 | 0.002 | **0.017** |
| delta | rh-precuneus | 0.0171 | 7 | 0.02 | **0.041** |
| delta | rh-cuneus | 0.0321 | 8 | 0.0059 | **0.02** |
| delta | rh-pericalcarine | 0.0261 | 8 | 0.0039 | **0.017** |
| delta | rh-lateraloccipital | 0.0285 | 8 | 0.0059 | **0.02** |
| delta | rh-lingual | 0.0291 | 9 | 0.002 | **0.017** |
| delta | rh-fusiform | 0.028 | 7 | 0.02 | **0.041** |
| delta | rh-parahippocampal | 0.0387 | 7 | 0.02 | **0.041** |
| delta | rh-entorhinal | 0.04 | 7 | 0.0098 | **0.028** |
| delta | rh-temporalpole | 0.0344 | 6 | 0.037 | 0.066 |
| delta | rh-inferiortemporal | 0.0307 | 8 | 0.0059 | **0.02** |
| delta | rh-middletemporal | 0.0325 | 7 | 0.0098 | **0.028** |
| delta | rh-bankssts | 0.0265 | 8 | 0.0039 | **0.017** |
| delta | rh-superiortemporal | 0.0283 | 7 | 0.0098 | **0.028** |
| delta | rh-transversetemporal | 0.0241 | 8 | 0.0059 | **0.02** |
| delta | rh-insula | 0.0391 | 8 | 0.014 | **0.034** |
| delta | Right-Hippocampus | 0.0378 | 7 | 0.014 | **0.034** |
| delta | Right-Amygdala | 0.0428 | 7 | 0.0098 | **0.028** |
| delta | lh-lateralorbitofrontal | 0.0243 | 8 | 0.0059 | **0.02** |
| delta | lh-parsorbitalis | 0.0323 | 8 | 0.0039 | **0.017** |
| delta | lh-frontalpole | 0.0557 | 9 | 0.002 | **0.017** |
| delta | lh-medialorbitofrontal | 0.0413 | 9 | 0.002 | **0.017** |
| delta | lh-parstriangularis | 0.039 | 9 | 0.002 | **0.017** |
| delta | lh-parsopercularis | 0.0474 | 8 | 0.0039 | **0.017** |
| delta | lh-rostralmiddlefrontal | 0.0403 | 9 | 0.002 | **0.017** |
| delta | lh-superiorfrontal | 0.0292 | 8 | 0.0039 | **0.017** |
| delta | lh-caudalmiddlefrontal | 0.0234 | 8 | 0.0059 | **0.02** |
| delta | lh-precentral | 0.0355 | 7 | 0.014 | **0.034** |
| delta | lh-paracentral | 0.0191 | 9 | 0.002 | **0.017** |
| delta | lh-rostralanteriorcingulate | 0.0405 | 9 | 0.002 | **0.017** |
| delta | lh-caudalanteriorcingulate | 0.0523 | 8 | 0.0039 | **0.017** |
| delta | lh-posteriorcingulate | 0.0331 | 9 | 0.002 | **0.017** |
| delta | lh-isthmuscingulate | 0.0243 | 8 | 0.0039 | **0.017** |
| delta | lh-postcentral | 0.0371 | 7 | 0.02 | **0.041** |
| delta | lh-supramarginal | 0.0203 | 8 | 0.0039 | **0.017** |
| delta | lh-superiorparietal | 0.0173 | 8 | 0.0039 | **0.017** |
| delta | lh-inferiorparietal | 0.0136 | 7 | 0.027 | 0.052 |
| delta | lh-precuneus | 0.0123 | 8 | 0.0059 | **0.02** |
| delta | lh-cuneus | 0.0281 | 8 | 0.0059 | **0.02** |
| delta | lh-pericalcarine | 0.0225 | 8 | 0.0039 | **0.017** |
| delta | lh-lateraloccipital | 0.0221 | 8 | 0.0039 | **0.017** |
| delta | lh-lingual | 0.0285 | 9 | 0.002 | **0.017** |
| delta | lh-fusiform | 0.0369 | 9 | 0.002 | **0.017** |
| delta | lh-parahippocampal | 0.0357 | 8 | 0.0039 | **0.017** |
| delta | lh-entorhinal | 0.0398 | 8 | 0.0039 | **0.017** |
| delta | lh-temporalpole | 0.0385 | 8 | 0.0039 | **0.017** |
| delta | lh-inferiortemporal | 0.0269 | 8 | 0.0039 | **0.017** |
| delta | lh-middletemporal | 0.0239 | 7 | 0.014 | **0.034** |
| delta | lh-bankssts | 0.021 | 8 | 0.0039 | **0.017** |
| delta | lh-superiortemporal | 0.0259 | 8 | 0.0059 | **0.02** |
| delta | lh-transversetemporal | 0.0447 | 9 | 0.002 | **0.017** |
| delta | lh-insula | 0.0486 | 8 | 0.0039 | **0.017** |
| delta | Left-Hippocampus | 0.039 | 9 | 0.002 | **0.017** |
| delta | Left-Amygdala | 0.0482 | 8 | 0.0039 | **0.017** |
| theta | rh-lateralorbitofrontal | 0.0391 | 9 | 0.002 | **0.017** |
| theta | rh-parsorbitalis | 0.0212 | 8 | 0.0039 | **0.017** |
| theta | rh-frontalpole | 0.0464 | 9 | 0.002 | **0.017** |
| theta | rh-medialorbitofrontal | 0.0342 | 9 | 0.002 | **0.017** |
| theta | rh-parstriangularis | 0.0231 | 9 | 0.002 | **0.017** |
| theta | rh-parsopercularis | 0.0181 | 7 | 0.049 | 0.081 |
| theta | rh-rostralmiddlefrontal | 0.0392 | 8 | 0.0039 | **0.017** |
| theta | rh-superiorfrontal | 0.0479 | 8 | 0.0039 | **0.017** |
| theta | rh-caudalmiddlefrontal | 0.0315 | 7 | 0.02 | **0.041** |
| theta | rh-precentral | 0.0223 | 6 | 0.082 | 0.12 |
| theta | rh-paracentral | 0.0229 | 8 | 0.027 | 0.052 |
| theta | rh-rostralanteriorcingulate | 0.027 | 9 | 0.002 | **0.017** |
| theta | rh-caudalanteriorcingulate | 0.0391 | 8 | 0.0039 | **0.017** |
| theta | rh-posteriorcingulate | 0.0292 | 7 | 0.014 | **0.034** |
| theta | rh-isthmuscingulate | 0.0324 | 9 | 0.002 | **0.017** |
| theta | rh-postcentral | 0.0214 | 6 | 0.1 | 0.14 |
| theta | rh-supramarginal | 0.0223 | 7 | 0.027 | 0.052 |
| theta | rh-superiorparietal | 0.0111 | 8 | 0.0039 | **0.017** |
| theta | rh-inferiorparietal | 0.0342 | 9 | 0.002 | **0.017** |
| theta | rh-precuneus | 0.0304 | 9 | 0.002 | **0.017** |
| theta | rh-cuneus | 0.0327 | 8 | 0.014 | **0.034** |
| theta | rh-pericalcarine | 0.0303 | 7 | 0.014 | **0.034** |
| theta | rh-lateraloccipital | 0.0222 | 7 | 0.02 | **0.041** |
| theta | rh-lingual | 0.0257 | 8 | 0.02 | **0.041** |
| theta | rh-fusiform | 0.0478 | 8 | 0.0059 | **0.02** |
| theta | rh-parahippocampal | 0.0417 | 8 | 0.014 | **0.034** |
| theta | rh-entorhinal | 0.014 | 7 | 0.027 | 0.052 |
| theta | rh-temporalpole | 0.023 | 7 | 0.037 | 0.066 |
| theta | rh-inferiortemporal | 0.00078 | 5 | 0.29 | 0.32 |
| theta | rh-middletemporal | 0.0166 | 7 | 0.027 | 0.052 |
| theta | rh-bankssts | 0.028 | 8 | 0.0039 | **0.017** |
| theta | rh-superiortemporal | 0.0125 | 7 | 0.037 | 0.066 |
| theta | rh-transversetemporal | 0.0282 | 7 | 0.02 | **0.041** |
| theta | rh-insula | 0.0125 | 8 | 0.0039 | **0.017** |
| theta | Right-Hippocampus | 0.0368 | 7 | 0.02 | **0.041** |
| theta | Right-Amygdala | 0.0294 | 8 | 0.0098 | **0.028** |
| theta | lh-lateralorbitofrontal | 0.0264 | 7 | 0.027 | 0.052 |
| theta | lh-parsorbitalis | 0.0373 | 6 | 0.037 | 0.066 |
| theta | lh-frontalpole | 0.0692 | 9 | 0.002 | **0.017** |
| theta | lh-medialorbitofrontal | 0.0238 | 9 | 0.002 | **0.017** |
| theta | lh-parstriangularis | 0.0295 | 7 | 0.027 | 0.052 |
| theta | lh-parsopercularis | 0.0219 | 8 | 0.0039 | **0.017** |
| theta | lh-rostralmiddlefrontal | 0.0242 | 9 | 0.002 | **0.017** |
| theta | lh-superiorfrontal | 0.0268 | 8 | 0.02 | **0.041** |
| theta | lh-caudalmiddlefrontal | 0.019 | 8 | 0.0039 | **0.017** |
| theta | lh-precentral | 0.0352 | 8 | 0.0098 | **0.028** |
| theta | lh-paracentral | 0.0287 | 8 | 0.02 | **0.041** |
| theta | lh-rostralanteriorcingulate | 0.0283 | 8 | 0.0039 | **0.017** |
| theta | lh-caudalanteriorcingulate | 0.0466 | 8 | 0.0098 | **0.028** |
| theta | lh-posteriorcingulate | 0.0297 | 8 | 0.02 | **0.041** |
| theta | lh-isthmuscingulate | 0.014 | 7 | 0.0098 | **0.028** |
| theta | lh-postcentral | 0.0217 | 7 | 0.049 | 0.081 |
| theta | lh-supramarginal | 0.0243 | 5 | 0.082 | 0.12 |
| theta | lh-superiorparietal | -0.00542 | 4 | 0.21 | 0.25 |
| theta | lh-inferiorparietal | 0.0164 | 7 | 0.13 | 0.17 |
| theta | lh-precuneus | 0.0209 | 8 | 0.0059 | **0.02** |
| theta | lh-cuneus | 0.0201 | 8 | 0.02 | **0.041** |
| theta | lh-pericalcarine | 0.0127 | 8 | 0.0039 | **0.017** |
| theta | lh-lateraloccipital | 0.0371 | 8 | 0.0039 | **0.017** |
| theta | lh-lingual | 0.0408 | 7 | 0.014 | **0.034** |
| theta | lh-fusiform | 0.0277 | 7 | 0.02 | **0.041** |
| theta | lh-parahippocampal | 0.0247 | 5 | 0.1 | 0.14 |
| theta | lh-entorhinal | 0.029 | 6 | 0.049 | 0.081 |
| theta | lh-temporalpole | 0.0338 | 7 | 0.027 | 0.052 |
| theta | lh-inferiortemporal | 0.0185 | 7 | 0.0098 | **0.028** |
| theta | lh-middletemporal | 0.0272 | 6 | 0.064 | 0.098 |
| theta | lh-bankssts | 0.0176 | 8 | 0.0039 | **0.017** |
| theta | lh-superiortemporal | 0.0282 | 6 | 0.049 | 0.081 |
| theta | lh-transversetemporal | 0.0221 | 6 | 0.082 | 0.12 |
| theta | lh-insula | 0.018 | 7 | 0.037 | 0.066 |
| theta | Left-Hippocampus | 0.022 | 7 | 0.064 | 0.098 |
| theta | Left-Amygdala | 0.028 | 6 | 0.027 | 0.052 |
| alpha | rh-lateralorbitofrontal | 0.0201 | 5 | 0.25 | 0.29 |
| alpha | rh-parsorbitalis | 0.00701 | 6 | 0.37 | 0.4 |
| alpha | rh-frontalpole | -0.00879 | 2 | 0.92 | 0.92 |
| alpha | rh-medialorbitofrontal | 0.0263 | 6 | 0.13 | 0.17 |
| alpha | rh-parstriangularis | 0.00116 | 5 | 0.25 | 0.29 |
| alpha | rh-parsopercularis | 0.0357 | 8 | 0.0039 | **0.017** |
| alpha | rh-rostralmiddlefrontal | 0.0203 | 7 | 0.15 | 0.19 |
| alpha | rh-superiorfrontal | 0.0248 | 7 | 0.02 | **0.041** |
| alpha | rh-caudalmiddlefrontal | 0.0168 | 7 | 0.037 | 0.066 |
| alpha | rh-precentral | 0.0373 | 8 | 0.0098 | **0.028** |
| alpha | rh-paracentral | 0.00719 | 6 | 0.13 | 0.17 |
| alpha | rh-rostralanteriorcingulate | 0.0444 | 7 | 0.014 | **0.034** |
| alpha | rh-caudalanteriorcingulate | 0.0401 | 8 | 0.0098 | **0.028** |
| alpha | rh-posteriorcingulate | 0.0145 | 6 | 0.049 | 0.081 |
| alpha | rh-isthmuscingulate | 0.0154 | 7 | 0.1 | 0.14 |
| alpha | rh-postcentral | 0.023 | 8 | 0.049 | 0.081 |
| alpha | rh-supramarginal | 0.00646 | 6 | 0.18 | 0.22 |
| alpha | rh-superiorparietal | 0.0153 | 6 | 0.037 | 0.066 |
| alpha | rh-inferiorparietal | 0.0145 | 6 | 0.21 | 0.25 |
| alpha | rh-precuneus | 0.0213 | 7 | 0.13 | 0.17 |
| alpha | rh-cuneus | 0.00736 | 5 | 0.29 | 0.32 |
| alpha | rh-pericalcarine | -0.00866 | 4 | 0.63 | 0.65 |
| alpha | rh-lateraloccipital | 0.00205 | 5 | 0.67 | 0.69 |
| alpha | rh-lingual | 0.00369 | 5 | 0.54 | 0.56 |
| alpha | rh-fusiform | 0.00514 | 5 | 0.33 | 0.37 |
| alpha | rh-parahippocampal | 0.0165 | 7 | 0.18 | 0.22 |
| alpha | rh-entorhinal | 0.00149 | 5 | 0.29 | 0.32 |
| alpha | rh-temporalpole | 0.00184 | 6 | 0.13 | 0.17 |
| alpha | rh-inferiortemporal | -0.0127 | 4 | 0.54 | 0.56 |
| alpha | rh-middletemporal | 0.00357 | 6 | 0.29 | 0.32 |
| alpha | rh-bankssts | 0.00965 | 7 | 0.13 | 0.17 |
| alpha | rh-superiortemporal | 0.0127 | 8 | 0.0039 | **0.017** |
| alpha | rh-transversetemporal | 0.00674 | 7 | 0.1 | 0.14 |
| alpha | rh-insula | 0.029 | 7 | 0.064 | 0.098 |
| alpha | Right-Hippocampus | 0.00964 | 6 | 0.25 | 0.29 |
| alpha | Right-Amygdala | 0.00425 | 5 | 0.15 | 0.19 |
| alpha | lh-lateralorbitofrontal | 0.0162 | 7 | 0.0098 | **0.028** |
| alpha | lh-parsorbitalis | 0.024 | 8 | 0.0098 | **0.028** |
| alpha | lh-frontalpole | 0.00648 | 6 | 0.37 | 0.4 |
| alpha | lh-medialorbitofrontal | 0.0204 | 6 | 0.064 | 0.098 |
| alpha | lh-parstriangularis | 0.0161 | 5 | 0.1 | 0.14 |
| alpha | lh-parsopercularis | -0.000786 | 4 | 0.25 | 0.29 |
| alpha | lh-rostralmiddlefrontal | 0.0279 | 7 | 0.0098 | **0.028** |
| alpha | lh-superiorfrontal | 0.0231 | 7 | 0.02 | **0.041** |
| alpha | lh-caudalmiddlefrontal | 0.0243 | 7 | 0.064 | 0.098 |
| alpha | lh-precentral | 0.017 | 5 | 0.15 | 0.19 |
| alpha | lh-paracentral | 0.0233 | 7 | 0.049 | 0.081 |
| alpha | lh-rostralanteriorcingulate | 0.0288 | 8 | 0.027 | 0.052 |
| alpha | lh-caudalanteriorcingulate | 0.0416 | 7 | 0.0098 | **0.028** |
| alpha | lh-posteriorcingulate | 0.0156 | 7 | 0.02 | **0.041** |
| alpha | lh-isthmuscingulate | 0.0175 | 7 | 0.15 | 0.19 |
| alpha | lh-postcentral | -0.00717 | 3 | 0.67 | 0.69 |
| alpha | lh-supramarginal | 0.0155 | 7 | 0.064 | 0.098 |
| alpha | lh-superiorparietal | 0.00251 | 6 | 0.13 | 0.17 |
| alpha | lh-inferiorparietal | 0.0111 | 6 | 0.21 | 0.25 |
| alpha | lh-precuneus | 0.0118 | 7 | 0.037 | 0.066 |
| alpha | lh-cuneus | 0.0274 | 8 | 0.014 | **0.034** |
| alpha | lh-pericalcarine | 0.00233 | 6 | 0.1 | 0.14 |
| alpha | lh-lateraloccipital | -0.0151 | 4 | 0.75 | 0.76 |
| alpha | lh-lingual | 0.00658 | 6 | 0.41 | 0.44 |
| alpha | lh-fusiform | -0.00306 | 4 | 0.21 | 0.25 |
| alpha | lh-parahippocampal | 0.00484 | 5 | 0.18 | 0.22 |
| alpha | lh-entorhinal | 0.00926 | 7 | 0.037 | 0.066 |
| alpha | lh-temporalpole | 0.0153 | 8 | 0.027 | 0.052 |
| alpha | lh-inferiortemporal | -0.00307 | 4 | 0.21 | 0.25 |
| alpha | lh-middletemporal | 0.0165 | 7 | 0.064 | 0.098 |
| alpha | lh-bankssts | 0.0216 | 7 | 0.082 | 0.12 |
| alpha | lh-superiortemporal | 0.019 | 7 | 0.049 | 0.081 |
| alpha | lh-transversetemporal | 0.026 | 6 | 0.1 | 0.14 |
| alpha | lh-insula | 0.00215 | 5 | 0.33 | 0.37 |
| alpha | Left-Hippocampus | -0.000789 | 4 | 0.41 | 0.44 |
| alpha | Left-Amygdala | 0.0091 | 7 | 0.064 | 0.098 |
| beta | rh-lateralorbitofrontal | 0.00599 | 7 | 0.064 | 0.098 |
| beta | rh-parsorbitalis | -0.00125 | 3 | 0.54 | 0.56 |
| beta | rh-frontalpole | 0.00988 | 6 | 0.064 | 0.098 |
| beta | rh-medialorbitofrontal | 0.00227 | 6 | 0.13 | 0.17 |
| beta | rh-parstriangularis | -0.00107 | 2 | 0.88 | 0.88 |
| beta | rh-parsopercularis | 0.000393 | 5 | 0.41 | 0.44 |
| beta | rh-rostralmiddlefrontal | 0.0113 | 5 | 0.18 | 0.22 |
| beta | rh-superiorfrontal | 0.015 | 7 | 0.014 | **0.034** |
| beta | rh-caudalmiddlefrontal | -0.00338 | 4 | 0.75 | 0.76 |
| beta | rh-precentral | 0.00146 | 5 | 0.54 | 0.56 |
| beta | rh-paracentral | 0.00872 | 6 | 0.1 | 0.14 |
| beta | rh-rostralanteriorcingulate | -0.000996 | 4 | 0.37 | 0.4 |
| beta | rh-caudalanteriorcingulate | -0.00122 | 4 | 0.46 | 0.48 |
| beta | rh-posteriorcingulate | 0.0104 | 6 | 0.064 | 0.098 |
| beta | rh-isthmuscingulate | 0.0051 | 5 | 0.29 | 0.32 |
| beta | rh-postcentral | -0.00252 | 4 | 0.21 | 0.25 |
| beta | rh-supramarginal | 0.00711 | 8 | 0.064 | 0.098 |
| beta | rh-superiorparietal | 0.000837 | 5 | 0.21 | 0.25 |
| beta | rh-inferiorparietal | 0.0121 | 6 | 0.13 | 0.17 |
| beta | rh-precuneus | 0.00305 | 6 | 0.13 | 0.17 |
| beta | rh-cuneus | 0.00298 | 7 | 0.15 | 0.19 |
| beta | rh-pericalcarine | 0.00256 | 6 | 0.18 | 0.22 |
| beta | rh-lateraloccipital | 0.0093 | 8 | 0.049 | 0.081 |
| beta | rh-lingual | 0.0165 | 6 | 0.13 | 0.17 |
| beta | rh-fusiform | 0.00565 | 6 | 0.064 | 0.098 |
| beta | rh-parahippocampal | 0.00133 | 7 | 0.064 | 0.098 |
| beta | rh-entorhinal | 0.00533 | 8 | 0.02 | **0.041** |
| beta | rh-temporalpole | 0.0102 | 5 | 0.18 | 0.22 |
| beta | rh-inferiortemporal | 0.00758 | 7 | 0.1 | 0.14 |
| beta | rh-middletemporal | 0.00801 | 7 | 0.0098 | **0.028** |
| beta | rh-bankssts | 0.007 | 5 | 0.15 | 0.19 |
| beta | rh-superiortemporal | 0.00775 | 5 | 0.082 | 0.12 |
| beta | rh-transversetemporal | 0.00384 | 5 | 0.15 | 0.19 |
| beta | rh-insula | 0.00852 | 7 | 0.049 | 0.081 |
| beta | Right-Hippocampus | 0.0115 | 8 | 0.02 | **0.041** |
| beta | Right-Amygdala | 0.011 | 7 | 0.037 | 0.066 |
| beta | lh-lateralorbitofrontal | 0.0028 | 6 | 0.41 | 0.44 |
| beta | lh-parsorbitalis | -0.00606 | 4 | 0.54 | 0.56 |
| beta | lh-frontalpole | 0.00808 | 8 | 0.02 | **0.041** |
| beta | lh-medialorbitofrontal | 0.00852 | 6 | 0.25 | 0.29 |
| beta | lh-parstriangularis | 0.00898 | 6 | 0.13 | 0.17 |
| beta | lh-parsopercularis | 0.0181 | 7 | 0.037 | 0.066 |
| beta | lh-rostralmiddlefrontal | 0.000957 | 6 | 0.15 | 0.19 |
| beta | lh-superiorfrontal | -0.00246 | 4 | 0.41 | 0.44 |
| beta | lh-caudalmiddlefrontal | -0.00114 | 4 | 0.21 | 0.25 |
| beta | lh-precentral | 0.00418 | 5 | 0.25 | 0.29 |
| beta | lh-paracentral | 0.00744 | 7 | 0.18 | 0.22 |
| beta | lh-rostralanteriorcingulate | 0.0098 | 6 | 0.29 | 0.32 |
| beta | lh-caudalanteriorcingulate | -0.00029 | 4 | 0.37 | 0.4 |
| beta | lh-posteriorcingulate | 0.0143 | 7 | 0.027 | 0.052 |
| beta | lh-isthmuscingulate | 0.00995 | 8 | 0.014 | **0.034** |
| beta | lh-postcentral | -0.00377 | 4 | 0.37 | 0.4 |
| beta | lh-supramarginal | -0.0054 | 4 | 0.41 | 0.44 |
| beta | lh-superiorparietal | 0.00709 | 6 | 0.082 | 0.12 |
| beta | lh-inferiorparietal | 0.000576 | 5 | 0.41 | 0.44 |
| beta | lh-precuneus | 0.0183 | 7 | 0.014 | **0.034** |
| beta | lh-cuneus | 0.00879 | 7 | 0.027 | 0.052 |
| beta | lh-pericalcarine | 0.0109 | 8 | 0.0059 | **0.02** |
| beta | lh-lateraloccipital | -0.000274 | 4 | 0.41 | 0.44 |
| beta | lh-lingual | 0.00733 | 6 | 0.25 | 0.29 |
| beta | lh-fusiform | 0.00283 | 5 | 0.5 | 0.53 |
| beta | lh-parahippocampal | 0.00542 | 7 | 0.064 | 0.098 |
| beta | lh-entorhinal | 0.00354 | 5 | 0.18 | 0.22 |
| beta | lh-temporalpole | -0.00724 | 3 | 0.9 | 0.9 |
| beta | lh-inferiortemporal | -0.00298 | 3 | 0.54 | 0.56 |
| beta | lh-middletemporal | -0.002 | 4 | 0.59 | 0.61 |
| beta | lh-bankssts | -0.00823 | 4 | 0.67 | 0.69 |
| beta | lh-superiortemporal | 0.00291 | 7 | 0.064 | 0.098 |
| beta | lh-transversetemporal | 0.00598 | 7 | 0.1 | 0.14 |
| beta | lh-insula | 0.0174 | 8 | 0.014 | **0.034** |
| beta | Left-Hippocampus | 0.0103 | 8 | 0.0059 | **0.02** |
| beta | Left-Amygdala | 0.0073 | 6 | 0.13 | 0.17 |
| #patients IED>no IED: number of patients for which the value in the ROI was higher in the scalp negative IED than the no IED condition. ROI names correspond to the ones of the Desikan atlas.  lh: left hemisphere, rh: right hemisphere, ROI: region of interest. | | | | | |

| Supplementary Table 13: iEEG(ROI) integration changes between scalp-negative IEDs vs no IED | | | | | |
| --- | --- | --- | --- | --- | --- |
| Frequency band | **ROI name** | **Median difference** | **#patients IED>no IED** | **P-value** | **Adjusted P‑value** |
| delta | rh-lateralorbitofrontal | 0.00549 | 6 | 0.039 | 0.054 |
| delta | rh-rostralmiddlefrontal | 0.00646 | 8 | 0.0059 | **0.029** |
| delta | rh-fusiform | 0.00315 | 6 | 0.078 | 0.089 |
| delta | rh-inferiortemporal | 0.00596 | 6 | 0.082 | 0.092 |
| delta | rh-middletemporal | -0.000468 | 4 | 0.25 | 0.25 |
| delta | Right-Hippocampus | 0.005 | 6 | 0.02 | **0.036** |
| delta | Right-Amygdala | 0.0023 | 5 | 0.13 | 0.13 |
| delta | lh-lateralorbitofrontal | 0.00928 | 6 | 0.016 | **0.03** |
| delta | lh-rostralmiddlefrontal | 0.0116 | 6 | 0.016 | **0.03** |
| delta | lh-inferiortemporal | 0.0028 | 7 | 0.0078 | **0.029** |
| delta | lh-middletemporal | 0.00441 | 5 | 0.039 | 0.054 |
| delta | Left-Hippocampus | 0.00301 | 4 | 0.16 | 0.16 |
| theta | rh-lateralorbitofrontal | 0.00554 | 7 | 0.0078 | **0.029** |
| theta | rh-rostralmiddlefrontal | 0.00672 | 6 | 0.049 | 0.062 |
| theta | rh-fusiform | 0.0059 | 6 | 0.016 | **0.03** |
| theta | rh-inferiortemporal | 0.00808 | 8 | 0.014 | **0.03** |
| theta | rh-middletemporal | 0.00632 | 6 | 0.064 | 0.079 |
| theta | Right-Hippocampus | 0.00939 | 7 | 0.027 | **0.044** |
| theta | Right-Amygdala | 0.0051 | 7 | 0.027 | **0.044** |
| theta | lh-lateralorbitofrontal | 0.00488 | 6 | 0.016 | **0.03** |
| theta | lh-rostralmiddlefrontal | 0.00568 | 6 | 0.016 | **0.03** |
| theta | lh-inferiortemporal | 0.00451 | 6 | 0.039 | 0.054 |
| theta | lh-middletemporal | 0.00699 | 7 | 0.0078 | **0.029** |
| theta | Left-Hippocampus | 0.00803 | 6 | 0.016 | **0.03** |
| alpha | rh-lateralorbitofrontal | 0.0048 | 7 | 0.0078 | **0.029** |
| alpha | rh-rostralmiddlefrontal | 0.0055 | 6 | 0.037 | 0.054 |
| alpha | rh-fusiform | 0.0169 | 6 | 0.023 | **0.042** |
| alpha | rh-inferiortemporal | 0.00728 | 8 | 0.014 | **0.03** |
| alpha | rh-middletemporal | 0.00288 | 6 | 0.049 | 0.062 |
| alpha | Right-Hippocampus | 0.0176 | 7 | 0.0078 | **0.029** |
| alpha | Right-Amygdala | 0.0143 | 7 | 0.0078 | **0.029** |
| alpha | lh-lateralorbitofrontal | 0.00725 | 6 | 0.016 | **0.03** |
| alpha | lh-rostralmiddlefrontal | 0.00297 | 5 | 0.078 | 0.089 |
| alpha | lh-inferiortemporal | 0.00617 | 5 | 0.11 | 0.12 |
| alpha | lh-middletemporal | 0.00444 | 4 | 0.15 | 0.15 |
| alpha | Left-Hippocampus | 0.00698 | 5 | 0.047 | 0.062 |
| beta | rh-lateralorbitofrontal | 0.00645 | 6 | 0.027 | **0.044** |
| beta | rh-rostralmiddlefrontal | 0.00313 | 8 | 0.0098 | **0.03** |
| beta | rh-fusiform | 0.0199 | 7 | 0.0078 | **0.029** |
| beta | rh-inferiortemporal | 0.00766 | 8 | 0.0039 | **0.029** |
| beta | rh-middletemporal | 0.00585 | 9 | 0.002 | **0.029** |
| beta | Right-Hippocampus | 0.0241 | 8 | 0.0039 | **0.029** |
| beta | Right-Amygdala | 0.0194 | 8 | 0.0039 | **0.029** |
| beta | lh-lateralorbitofrontal | 0.00659 | 6 | 0.016 | **0.03** |
| beta | lh-rostralmiddlefrontal | 0.00259 | 5 | 0.078 | 0.089 |
| beta | lh-inferiortemporal | 0.00428 | 7 | 0.0078 | **0.029** |
| beta | lh-middletemporal | 0.00329 | 6 | 0.016 | **0.03** |
| beta | Left-Hippocampus | 0.00422 | 5 | 0.031 | **0.048** |
| #patients IED>no IED: number of patients for which the value in the ROI was higher in the scalp negative IED than the no IED condition. ROI names correspond to the ones of the Desikan atlas.  lh: left hemisphere, rh: right hemisphere, ROI: region of interest. | | | | | |

| Supplementary Table 14: hdEEG(ROI) segregation changes between scalp-negative IEDs vs no IED | | | | | |
| --- | --- | --- | --- | --- | --- |
| Frequency band | **ROI name** | **Median difference** | **#patients IED>no IED** | **P-value** | **Adjusted P‑value** |
| delta | rh-lateralorbitofrontal | 0.0325 | 9 | 0.002 | **0.01** |
| delta | rh-parsorbitalis | 0.04 | 9 | 0.002 | **0.01** |
| delta | rh-frontalpole | 0.0385 | 9 | 0.002 | **0.01** |
| delta | rh-medialorbitofrontal | 0.0348 | 9 | 0.002 | **0.01** |
| delta | rh-parstriangularis | 0.0326 | 8 | 0.0039 | **0.013** |
| delta | rh-parsopercularis | 4.04E-02 | 9 | 0.002 | **0.01** |
| delta | rh-rostralmiddlefrontal | 0.0365 | 9 | 0.002 | **0.01** |
| delta | rh-superiorfrontal | 0.0414 | 9 | 0.002 | **0.01** |
| delta | rh-caudalmiddlefrontal | 0.0273 | 9 | 0.002 | **0.01** |
| delta | rh-precentral | 0.0144 | 8 | 0.0059 | **0.016** |
| delta | rh-paracentral | 0.0199 | 8 | 0.014 | **0.026** |
| delta | rh-rostralanteriorcingulate | 3.60E-02 | 9 | 0.002 | **0.01** |
| delta | rh-caudalanteriorcingulate | 0.047 | 9 | 0.002 | **0.01** |
| delta | rh-posteriorcingulate | 0.0259 | 8 | 0.0059 | **0.016** |
| delta | rh-isthmuscingulate | 0.0381 | 8 | 0.0098 | **0.021** |
| delta | rh-postcentral | 0.016 | 7 | 0.014 | **0.026** |
| delta | rh-supramarginal | 0.0176 | 8 | 0.0039 | **0.013** |
| delta | rh-superiorparietal | 1.16E-02 | 9 | 0.002 | **0.01** |
| delta | rh-inferiorparietal | 0.0299 | 9 | 0.002 | **0.01** |
| delta | rh-precuneus | 0.0215 | 7 | 0.0098 | **0.021** |
| delta | rh-cuneus | 0.0268 | 8 | 0.0039 | **0.013** |
| delta | rh-pericalcarine | 0.0253 | 8 | 0.0039 | **0.013** |
| delta | rh-lateraloccipital | 0.0244 | 8 | 0.0039 | **0.013** |
| delta | rh-lingual | 0.029 | 9 | 0.002 | **0.01** |
| delta | rh-fusiform | 0.0304 | 7 | 0.014 | **0.026** |
| delta | rh-parahippocampal | 0.0371 | 7 | 0.02 | **0.035** |
| delta | rh-entorhinal | 0.0329 | 7 | 0.0098 | **0.021** |
| delta | rh-temporalpole | 0.0293 | 8 | 0.0059 | **0.016** |
| delta | rh-inferiortemporal | 0.0307 | 8 | 0.0059 | **0.016** |
| delta | rh-middletemporal | 2.80E-02 | 8 | 0.0059 | **0.016** |
| delta | rh-bankssts | 0.0254 | 8 | 0.0039 | **0.013** |
| delta | rh-superiortemporal | 0.0248 | 9 | 0.002 | **0.01** |
| delta | rh-transversetemporal | 0.0224 | 8 | 0.0059 | **0.016** |
| delta | rh-insula | 0.0334 | 8 | 0.0098 | **0.021** |
| delta | Right-Hippocampus | 0.033 | 7 | 0.014 | **0.026** |
| delta | Right-Amygdala | 0.0339 | 7 | 0.0098 | **0.021** |
| delta | lh-lateralorbitofrontal | 0.0311 | 8 | 0.0039 | **0.013** |
| delta | lh-parsorbitalis | 0.0364 | 9 | 0.002 | **0.01** |
| delta | lh-frontalpole | 0.036 | 9 | 0.002 | **0.01** |
| delta | lh-medialorbitofrontal | 0.0293 | 9 | 0.002 | **0.01** |
| delta | lh-parstriangularis | 0.0348 | 9 | 0.002 | **0.01** |
| delta | lh-parsopercularis | 3.93E-02 | 9 | 0.002 | **0.01** |
| delta | lh-rostralmiddlefrontal | 0.0342 | 9 | 0.002 | **0.01** |
| delta | lh-superiorfrontal | 0.0339 | 9 | 0.002 | **0.01** |
| delta | lh-caudalmiddlefrontal | 0.0277 | 8 | 0.0039 | **0.013** |
| delta | lh-precentral | 0.0309 | 7 | 0.0098 | **0.021** |
| delta | lh-paracentral | 0.0228 | 9 | 0.002 | **0.01** |
| delta | lh-rostralanteriorcingulate | 0.036 | 9 | 0.002 | **0.01** |
| delta | lh-caudalanteriorcingulate | 0.0425 | 9 | 0.002 | **0.01** |
| delta | lh-posteriorcingulate | 0.0334 | 9 | 0.002 | **0.01** |
| delta | lh-isthmuscingulate | 0.0232 | 9 | 0.002 | **0.01** |
| delta | lh-postcentral | 0.0354 | 8 | 0.0098 | **0.021** |
| delta | lh-supramarginal | 0.018 | 9 | 0.002 | **0.01** |
| delta | lh-superiorparietal | 0.0219 | 9 | 0.002 | **0.01** |
| delta | lh-inferiorparietal | 0.0157 | 8 | 0.0059 | **0.016** |
| delta | lh-precuneus | 0.0121 | 9 | 0.002 | **0.01** |
| delta | lh-cuneus | 0.0241 | 8 | 0.0039 | **0.013** |
| delta | lh-pericalcarine | 0.023 | 9 | 0.002 | **0.01** |
| delta | lh-lateraloccipital | 0.0177 | 9 | 0.002 | **0.01** |
| delta | lh-lingual | 0.0274 | 9 | 0.002 | **0.01** |
| delta | lh-fusiform | 0.0283 | 9 | 0.002 | **0.01** |
| delta | lh-parahippocampal | 0.0296 | 8 | 0.0039 | **0.013** |
| delta | lh-entorhinal | 0.0397 | 8 | 0.0039 | **0.013** |
| delta | lh-temporalpole | 0.0361 | 8 | 0.0039 | **0.013** |
| delta | lh-inferiortemporal | 0.0254 | 9 | 0.002 | **0.01** |
| delta | lh-middletemporal | 0.028 | 8 | 0.0098 | **0.021** |
| delta | lh-bankssts | 0.0235 | 9 | 0.002 | **0.01** |
| delta | lh-superiortemporal | 0.0329 | 9 | 0.002 | **0.01** |
| delta | lh-transversetemporal | 0.0461 | 9 | 0.002 | **0.01** |
| delta | lh-insula | 0.0467 | 8 | 0.0039 | **0.013** |
| delta | Left-Hippocampus | 0.0376 | 9 | 0.002 | **0.01** |
| delta | Left-Amygdala | 0.0418 | 8 | 0.0039 | **0.013** |
| theta | rh-lateralorbitofrontal | 0.0335 | 8 | 0.0039 | **0.013** |
| theta | rh-parsorbitalis | 0.0233 | 8 | 0.0039 | **0.013** |
| theta | rh-frontalpole | 0.0279 | 9 | 0.002 | **0.01** |
| theta | rh-medialorbitofrontal | 0.0284 | 8 | 0.0059 | **0.016** |
| theta | rh-parstriangularis | 0.0151 | 9 | 0.002 | **0.01** |
| theta | rh-parsopercularis | 0.0193 | 7 | 0.037 | 0.055 |
| theta | rh-rostralmiddlefrontal | 0.0341 | 7 | 0.0098 | **0.021** |
| theta | rh-superiorfrontal | 0.0333 | 8 | 0.0039 | **0.013** |
| theta | rh-caudalmiddlefrontal | 0.0164 | 7 | 0.02 | **0.035** |
| theta | rh-precentral | 0.0187 | 7 | 0.027 | **0.043** |
| theta | rh-paracentral | 0.0233 | 8 | 0.014 | **0.026** |
| theta | rh-rostralanteriorcingulate | 0.0223 | 9 | 0.002 | **0.01** |
| theta | rh-caudalanteriorcingulate | 0.0302 | 8 | 0.0039 | **0.013** |
| theta | rh-posteriorcingulate | 0.0285 | 7 | 0.0098 | **0.021** |
| theta | rh-isthmuscingulate | 0.0255 | 9 | 0.002 | **0.01** |
| theta | rh-postcentral | 0.0141 | 5 | 0.13 | 0.15 |
| theta | rh-supramarginal | 0.0163 | 7 | 0.027 | **0.043** |
| theta | rh-superiorparietal | 0.0138 | 8 | 0.0059 | **0.016** |
| theta | rh-inferiorparietal | 0.0246 | 9 | 0.002 | **0.01** |
| theta | rh-precuneus | 0.0232 | 8 | 0.0039 | **0.013** |
| theta | rh-cuneus | 0.0244 | 8 | 0.014 | **0.026** |
| theta | rh-pericalcarine | 0.0225 | 7 | 0.0098 | **0.021** |
| theta | rh-lateraloccipital | 0.0201 | 7 | 0.0098 | **0.021** |
| theta | rh-lingual | 0.0227 | 7 | 0.027 | **0.043** |
| theta | rh-fusiform | 0.0352 | 8 | 0.0059 | **0.016** |
| theta | rh-parahippocampal | 0.0351 | 7 | 0.0098 | **0.021** |
| theta | rh-entorhinal | 0.0124 | 8 | 0.014 | **0.026** |
| theta | rh-temporalpole | 0.0188 | 6 | 0.037 | 0.055 |
| theta | rh-inferiortemporal | 0.00214 | 6 | 0.15 | 0.17 |
| theta | rh-middletemporal | 0.0168 | 6 | 0.027 | **0.043** |
| theta | rh-bankssts | 0.0193 | 9 | 0.002 | **0.01** |
| theta | rh-superiortemporal | 0.0102 | 7 | 0.037 | 0.055 |
| theta | rh-transversetemporal | 0.0245 | 8 | 0.0059 | **0.016** |
| theta | rh-insula | 0.017 | 9 | 0.002 | **0.01** |
| theta | Right-Hippocampus | 0.0322 | 8 | 0.0098 | **0.021** |
| theta | Right-Amygdala | 0.0149 | 8 | 0.0059 | **0.016** |
| theta | lh-lateralorbitofrontal | 0.0304 | 7 | 0.02 | **0.035** |
| theta | lh-parsorbitalis | 0.0295 | 7 | 0.0098 | **0.021** |
| theta | lh-frontalpole | 0.0499 | 9 | 0.002 | **0.01** |
| theta | lh-medialorbitofrontal | 0.0188 | 8 | 0.0039 | **0.013** |
| theta | lh-parstriangularis | 0.029 | 7 | 0.014 | **0.026** |
| theta | lh-parsopercularis | 0.0223 | 8 | 0.0039 | **0.013** |
| theta | lh-rostralmiddlefrontal | 0.021 | 9 | 0.002 | **0.01** |
| theta | lh-superiorfrontal | 0.0153 | 7 | 0.0098 | **0.021** |
| theta | lh-caudalmiddlefrontal | 0.022 | 9 | 0.002 | **0.01** |
| theta | lh-precentral | 0.0275 | 8 | 0.0059 | **0.016** |
| theta | lh-paracentral | 0.0215 | 8 | 0.02 | **0.035** |
| theta | lh-rostralanteriorcingulate | 0.0217 | 8 | 0.0039 | **0.013** |
| theta | lh-caudalanteriorcingulate | 0.0397 | 8 | 0.0059 | **0.016** |
| theta | lh-posteriorcingulate | 0.0291 | 8 | 0.014 | **0.026** |
| theta | lh-isthmuscingulate | 0.022 | 9 | 0.002 | **0.01** |
| theta | lh-postcentral | 0.0129 | 7 | 0.0098 | **0.021** |
| theta | lh-supramarginal | 0.0216 | 7 | 0.02 | **0.035** |
| theta | lh-superiorparietal | 0.00747 | 7 | 0.049 | 0.071 |
| theta | lh-inferiorparietal | 0.0167 | 7 | 0.15 | 0.17 |
| theta | lh-precuneus | 0.0209 | 8 | 0.0039 | **0.013** |
| theta | lh-cuneus | 0.0194 | 7 | 0.027 | **0.043** |
| theta | lh-pericalcarine | 0.0143 | 8 | 0.0039 | **0.013** |
| theta | lh-lateraloccipital | 0.0162 | 9 | 0.002 | **0.01** |
| theta | lh-lingual | 0.0239 | 8 | 0.0059 | **0.016** |
| theta | lh-fusiform | 0.0253 | 7 | 0.0098 | **0.021** |
| theta | lh-parahippocampal | 0.0126 | 6 | 0.064 | 0.087 |
| theta | lh-entorhinal | 2.36E-02 | 6 | 0.027 | **0.043** |
| theta | lh-temporalpole | 0.0284 | 7 | 0.02 | **0.035** |
| theta | lh-inferiortemporal | 0.0188 | 9 | 0.002 | **0.01** |
| theta | lh-middletemporal | 0.0227 | 6 | 0.037 | 0.055 |
| theta | lh-bankssts | 0.0152 | 8 | 0.0039 | **0.013** |
| theta | lh-superiortemporal | 0.0275 | 6 | 0.027 | **0.043** |
| theta | lh-transversetemporal | 0.0169 | 6 | 0.037 | 0.055 |
| theta | lh-insula | 0.0193 | 7 | 0.0098 | **0.021** |
| theta | Left-Hippocampus | 0.0216 | 7 | 0.027 | **0.043** |
| theta | Left-Amygdala | 0.025 | 6 | 0.027 | **0.043** |
| alpha | rh-lateralorbitofrontal | 0.00557 | 5 | 0.25 | 0.27 |
| alpha | rh-parsorbitalis | 0.0104 | 5 | 0.29 | 0.3 |
| alpha | rh-frontalpole | -0.00181 | 4 | 0.79 | 0.79 |
| alpha | rh-medialorbitofrontal | 0.0127 | 6 | 0.18 | 0.2 |
| alpha | rh-parstriangularis | 0.00172 | 5 | 0.25 | 0.27 |
| alpha | rh-parsopercularis | 0.0286 | 8 | 0.0039 | **0.013** |
| alpha | rh-rostralmiddlefrontal | 0.0161 | 7 | 0.13 | 0.15 |
| alpha | rh-superiorfrontal | 0.0232 | 6 | 0.027 | **0.043** |
| alpha | rh-caudalmiddlefrontal | 0.0179 | 7 | 0.0098 | **0.021** |
| alpha | rh-precentral | 0.0278 | 8 | 0.0059 | **0.016** |
| alpha | rh-paracentral | 0.0138 | 7 | 0.064 | 0.087 |
| alpha | rh-rostralanteriorcingulate | 0.0304 | 7 | 0.014 | **0.026** |
| alpha | rh-caudalanteriorcingulate | 0.0278 | 8 | 0.0098 | **0.021** |
| alpha | rh-posteriorcingulate | 0.0117 | 7 | 0.0098 | **0.021** |
| alpha | rh-isthmuscingulate | 0.0127 | 7 | 0.064 | 0.087 |
| alpha | rh-postcentral | 0.0154 | 8 | 0.049 | 0.071 |
| alpha | rh-supramarginal | 0.00772 | 7 | 0.1 | 0.13 |
| alpha | rh-superiorparietal | 0.029 | 7 | 0.0098 | **0.021** |
| alpha | rh-inferiorparietal | 0.014 | 6 | 0.064 | 0.087 |
| alpha | rh-precuneus | 0.0115 | 6 | 0.15 | 0.17 |
| alpha | rh-cuneus | 0.00213 | 5 | 0.41 | 0.43 |
| alpha | rh-pericalcarine | -0.00895 | 4 | 0.5 | 0.51 |
| alpha | rh-lateraloccipital | -0.00239 | 4 | 0.46 | 0.47 |
| alpha | rh-lingual | 0.00545 | 5 | 0.29 | 0.3 |
| alpha | rh-fusiform | 0.00644 | 6 | 0.21 | 0.24 |
| alpha | rh-parahippocampal | 0.0141 | 7 | 0.082 | 0.11 |
| alpha | rh-entorhinal | 0.00449 | 6 | 0.18 | 0.2 |
| alpha | rh-temporalpole | 0.00529 | 8 | 0.027 | **0.043** |
| alpha | rh-inferiortemporal | 0.00552 | 5 | 0.29 | 0.3 |
| alpha | rh-middletemporal | 0.000175 | 5 | 0.18 | 0.2 |
| alpha | rh-bankssts | 0.0049 | 7 | 0.1 | 0.13 |
| alpha | rh-superiortemporal | 0.00752 | 9 | 0.002 | **0.01** |
| alpha | rh-transversetemporal | 0.00942 | 7 | 0.049 | 0.071 |
| alpha | rh-insula | 0.0161 | 8 | 0.02 | **0.035** |
| alpha | Right-Hippocampus | 0.00857 | 7 | 0.064 | 0.087 |
| alpha | Right-Amygdala | 0.00828 | 7 | 0.027 | **0.043** |
| alpha | lh-lateralorbitofrontal | 0.0118 | 9 | 0.002 | **0.01** |
| alpha | lh-parsorbitalis | 0.0197 | 7 | 0.0098 | **0.021** |
| alpha | lh-frontalpole | 0.014 | 7 | 0.064 | 0.087 |
| alpha | lh-medialorbitofrontal | 0.0158 | 8 | 0.014 | **0.026** |
| alpha | lh-parstriangularis | 0.0163 | 7 | 0.027 | **0.043** |
| alpha | lh-parsopercularis | 0.0118 | 6 | 0.082 | 0.11 |
| alpha | lh-rostralmiddlefrontal | 0.0162 | 8 | 0.0039 | **0.013** |
| alpha | lh-superiorfrontal | 0.0267 | 8 | 0.0098 | **0.021** |
| alpha | lh-caudalmiddlefrontal | 0.0135 | 7 | 0.027 | **0.043** |
| alpha | lh-precentral | 0.0201 | 5 | 0.15 | 0.17 |
| alpha | lh-paracentral | 0.00883 | 8 | 0.027 | **0.043** |
| alpha | lh-rostralanteriorcingulate | 0.0275 | 9 | 0.002 | **0.01** |
| alpha | lh-caudalanteriorcingulate | 0.035 | 8 | 0.0039 | **0.013** |
| alpha | lh-posteriorcingulate | 0.0158 | 8 | 0.0059 | **0.016** |
| alpha | lh-isthmuscingulate | 0.00679 | 5 | 0.37 | 0.38 |
| alpha | lh-postcentral | -0.000933 | 4 | 0.5 | 0.51 |
| alpha | lh-supramarginal | 0.00877 | 8 | 0.014 | **0.026** |
| alpha | lh-superiorparietal | 0.00582 | 7 | 0.064 | 0.087 |
| alpha | lh-inferiorparietal | 0.0096 | 7 | 0.02 | **0.035** |
| alpha | lh-precuneus | 0.00662 | 6 | 0.15 | 0.17 |
| alpha | lh-cuneus | 0.0163 | 8 | 0.02 | **0.035** |
| alpha | lh-pericalcarine | 0.0136 | 7 | 0.037 | 0.055 |
| alpha | lh-lateraloccipital | -0.000426 | 4 | 0.5 | 0.51 |
| alpha | lh-lingual | 0.00259 | 7 | 0.18 | 0.2 |
| alpha | lh-fusiform | -0.00245 | 4 | 0.21 | 0.24 |
| alpha | lh-parahippocampal | 0.0048 | 6 | 0.1 | 0.13 |
| alpha | lh-entorhinal | 0.00856 | 5 | 0.1 | 0.13 |
| alpha | lh-temporalpole | 0.0168 | 8 | 0.0059 | **0.016** |
| alpha | lh-inferiortemporal | 0.000386 | 6 | 0.13 | 0.15 |
| alpha | lh-middletemporal | 0.0274 | 7 | 0.02 | **0.035** |
| alpha | lh-bankssts | 0.0147 | 7 | 0.064 | 0.087 |
| alpha | lh-superiortemporal | 0.018 | 7 | 0.049 | 0.071 |
| alpha | lh-transversetemporal | 0.0225 | 6 | 0.1 | 0.13 |
| alpha | lh-insula | 0.0102 | 7 | 0.064 | 0.087 |
| alpha | Left-Hippocampus | 0.00105 | 6 | 0.13 | 0.15 |
| alpha | Left-Amygdala | 0.00889 | 7 | 0.037 | 0.055 |
| beta | rh-lateralorbitofrontal | 0.00936 | 8 | 0.027 | **0.043** |
| beta | rh-parsorbitalis | -9.12E-04 | 3 | 0.5 | 0.51 |
| beta | rh-frontalpole | 0.00781 | 6 | 0.037 | 0.055 |
| beta | rh-medialorbitofrontal | 0.00393 | 6 | 0.13 | 0.15 |
| beta | rh-parstriangularis | -0.00185 | 4 | 0.63 | 0.64 |
| beta | rh-parsopercularis | 0.000976 | 5 | 0.33 | 0.35 |
| beta | rh-rostralmiddlefrontal | 0.00881 | 6 | 0.1 | 0.13 |
| beta | rh-superiorfrontal | 0.00824 | 9 | 0.002 | **0.01** |
| beta | rh-caudalmiddlefrontal | -0.000944 | 4 | 0.63 | 0.64 |
| beta | rh-precentral | 0.00159 | 6 | 0.29 | 0.3 |
| beta | rh-paracentral | 0.00709 | 8 | 0.037 | 0.055 |
| beta | rh-rostralanteriorcingulate | -0.000307 | 4 | 0.25 | 0.27 |
| beta | rh-caudalanteriorcingulate | -0.00192 | 4 | 0.46 | 0.47 |
| beta | rh-posteriorcingulate | 0.0118 | 8 | 0.02 | **0.035** |
| beta | rh-isthmuscingulate | 0.00325 | 5 | 0.18 | 0.2 |
| beta | rh-postcentral | 0.000309 | 5 | 0.18 | 0.2 |
| beta | rh-supramarginal | 0.00756 | 8 | 0.064 | 0.087 |
| beta | rh-superiorparietal | 0.00588 | 6 | 0.1 | 0.13 |
| beta | rh-inferiorparietal | 0.0108 | 7 | 0.02 | **0.035** |
| beta | rh-precuneus | 0.004 | 6 | 0.1 | 0.13 |
| beta | rh-cuneus | 0.00443 | 6 | 0.13 | 0.15 |
| beta | rh-pericalcarine | 0.00463 | 6 | 0.064 | 0.087 |
| beta | rh-lateraloccipital | 0.0104 | 8 | 0.02 | **0.035** |
| beta | rh-lingual | 0.0135 | 7 | 0.082 | 0.11 |
| beta | rh-fusiform | 0.0104 | 8 | 0.014 | **0.026** |
| beta | rh-parahippocampal | 1.05E-02 | 7 | 0.027 | **0.043** |
| beta | rh-entorhinal | 0.00855 | 8 | 0.014 | **0.026** |
| beta | rh-temporalpole | 0.0105 | 6 | 0.1 | 0.13 |
| beta | rh-inferiortemporal | 0.00704 | 7 | 0.1 | 0.13 |
| beta | rh-middletemporal | 0.00809 | 7 | 0.0098 | **0.021** |
| beta | rh-bankssts | 0.00659 | 7 | 0.049 | 0.071 |
| beta | rh-superiortemporal | 0.00536 | 6 | 0.027 | **0.043** |
| beta | rh-transversetemporal | 0.000957 | 5 | 0.18 | 0.2 |
| beta | rh-insula | 0.00702 | 7 | 0.064 | 0.087 |
| beta | Right-Hippocampus | 0.0125 | 8 | 0.0098 | **0.021** |
| beta | Right-Amygdala | 0.00789 | 8 | 0.027 | **0.043** |
| beta | lh-lateralorbitofrontal | 0.00499 | 6 | 0.37 | 0.38 |
| beta | lh-parsorbitalis | -0.00182 | 4 | 0.46 | 0.47 |
| beta | lh-frontalpole | 0.00894 | 8 | 0.0039 | **0.013** |
| beta | lh-medialorbitofrontal | 0.00984 | 6 | 0.25 | 0.27 |
| beta | lh-parstriangularis | 0.00753 | 6 | 0.064 | 0.087 |
| beta | lh-parsopercularis | 0.0136 | 8 | 0.027 | **0.043** |
| beta | lh-rostralmiddlefrontal | 0.00137 | 5 | 0.13 | 0.15 |
| beta | lh-superiorfrontal | 0.00309 | 6 | 0.15 | 0.17 |
| beta | lh-caudalmiddlefrontal | 0.00243 | 6 | 0.13 | 0.15 |
| beta | lh-precentral | 0.00262 | 5 | 0.21 | 0.24 |
| beta | lh-paracentral | 0.00794 | 7 | 0.082 | 0.11 |
| beta | lh-rostralanteriorcingulate | 0.00894 | 6 | 0.18 | 0.2 |
| beta | lh-caudalanteriorcingulate | 0.00532 | 6 | 0.082 | 0.11 |
| beta | lh-posteriorcingulate | 0.009 | 7 | 0.014 | **0.026** |
| beta | lh-isthmuscingulate | 0.00925 | 9 | 0.002 | **0.01** |
| beta | lh-postcentral | -7.65E-05 | 4 | 0.25 | 0.27 |
| beta | lh-supramarginal | 0.00085 | 5 | 0.33 | 0.35 |
| beta | lh-superiorparietal | 0.00512 | 7 | 0.064 | 0.087 |
| beta | lh-inferiorparietal | 0.00306 | 7 | 0.15 | 0.17 |
| beta | lh-precuneus | 0.0157 | 7 | 0.0098 | **0.021** |
| beta | lh-cuneus | 0.0101 | 7 | 0.014 | **0.026** |
| beta | lh-pericalcarine | 0.0127 | 8 | 0.0059 | **0.016** |
| beta | lh-lateraloccipital | 0.0027 | 6 | 0.1 | 0.13 |
| beta | lh-lingual | 0.00739 | 6 | 0.15 | 0.17 |
| beta | lh-fusiform | 0.00327 | 6 | 0.21 | 0.24 |
| beta | lh-parahippocampal | 0.00573 | 7 | 0.037 | 0.055 |
| beta | lh-entorhinal | 0.00226 | 5 | 0.13 | 0.15 |
| beta | lh-temporalpole | -0.00135 | 3 | 0.75 | 0.75 |
| beta | lh-inferiortemporal | 0.0018 | 5 | 0.25 | 0.27 |
| beta | lh-middletemporal | 0.000214 | 5 | 0.37 | 0.38 |
| beta | lh-bankssts | -0.00399 | 4 | 0.54 | 0.55 |
| beta | lh-superiortemporal | 0.0038 | 6 | 0.064 | 0.087 |
| beta | lh-transversetemporal | 0.00519 | 7 | 0.049 | 0.071 |
| beta | lh-insula | 0.0158 | 8 | 0.0098 | **0.021** |
| beta | Left-Hippocampus | 0.00837 | 8 | 0.0098 | **0.021** |
| beta | Left-Amygdala | 0.00684 | 6 | 0.082 | 0.11 |
| #patients IED>no IED: number of patients for which the value in the ROI was higher in the scalp negative IED than the no IED condition. ROI names correspond to the ones of the Desikan atlas.  lh: left hemisphere, rh: right hemisphere, ROI: region of interest. | | | | | |

| Supplementary Table 15: iEEG(ROI) segregation changes between scalp-negative IEDs vs no IED | | | | | |
| --- | --- | --- | --- | --- | --- |
| Frequency band | **ROI name** | **Median difference** | **#patients IED>no IED** | **P-value** | **Adjusted P‑value** |
| delta | rh-lateralorbitofrontal | 0.0149 | 8 | 0.0039 | **0.023** |
| delta | rh-rostralmiddlefrontal | 0.0199 | 8 | 0.0039 | **0.023** |
| delta | rh-fusiform | 0.0197 | 6 | 0.039 | 0.051 |
| delta | rh-inferiortemporal | 0.0163 | 6 | 0.049 | 0.059 |
| delta | rh-middletemporal | 0.00861 | 6 | 0.082 | 0.086 |
| delta | Right-Hippocampus | 1.74E-02 | 6 | 0.027 | **0.044** |
| delta | Right-Amygdala | 0.0171 | 5 | 0.13 | 0.13 |
| delta | lh-lateralorbitofrontal | 0.0237 | 6 | 0.016 | **0.034** |
| delta | lh-rostralmiddlefrontal | 0.0304 | 6 | 0.016 | **0.034** |
| delta | lh-inferiortemporal | 0.0105 | 7 | 0.0078 | **0.027** |
| delta | lh-middletemporal | 0.0134 | 5 | 0.055 | 0.06 |
| delta | Left-Hippocampus | 7.79E-03 | 4 | 0.16 | 0.16 |
| theta | rh-lateralorbitofrontal | 0.0234 | 7 | 0.02 | **0.036** |
| theta | rh-rostralmiddlefrontal | 0.0246 | 7 | 0.02 | **0.036** |
| theta | rh-fusiform | 0.0174 | 7 | 0.0078 | **0.027** |
| theta | rh-inferiortemporal | 0.0264 | 8 | 0.037 | 0.051 |
| theta | rh-middletemporal | 0.0198 | 7 | 0.049 | 0.059 |
| theta | Right-Hippocampus | 2.46E-02 | 7 | 0.055 | 0.06 |
| theta | Right-Amygdala | 0.0169 | 7 | 0.055 | 0.06 |
| theta | lh-lateralorbitofrontal | 0.0203 | 6 | 0.016 | **0.034** |
| theta | lh-rostralmiddlefrontal | 0.0149 | 6 | 0.016 | **0.034** |
| theta | lh-inferiortemporal | 0.015 | 6 | 0.016 | **0.034** |
| theta | lh-middletemporal | 0.0245 | 7 | 0.0078 | **0.027** |
| theta | Left-Hippocampus | 0.0225 | 5 | 0.031 | **0.044** |
| alpha | rh-lateralorbitofrontal | 0.022 | 7 | 0.039 | 0.051 |
| alpha | rh-rostralmiddlefrontal | 0.0184 | 6 | 0.064 | 0.069 |
| alpha | rh-fusiform | 0.0477 | 6 | 0.023 | **0.04** |
| alpha | rh-inferiortemporal | 0.0255 | 8 | 0.027 | **0.044** |
| alpha | rh-middletemporal | 0.0151 | 7 | 0.049 | 0.059 |
| alpha | Right-Hippocampus | 4.71E-02 | 7 | 0.02 | **0.036** |
| alpha | Right-Amygdala | 0.0387 | 7 | 0.02 | **0.036** |
| alpha | lh-lateralorbitofrontal | 0.0221 | 6 | 0.016 | **0.034** |
| alpha | lh-rostralmiddlefrontal | 0.0156 | 5 | 0.031 | **0.044** |
| alpha | lh-inferiortemporal | 0.0113 | 6 | 0.023 | **0.04** |
| alpha | lh-middletemporal | 0.0183 | 5 | 0.055 | 0.06 |
| alpha | Left-Hippocampus | 0.0202 | 5 | 0.031 | **0.044** |
| beta | rh-lateralorbitofrontal | 0.0207 | 8 | 0.0039 | **0.023** |
| beta | rh-rostralmiddlefrontal | 0.0144 | 9 | 0.002 | **0.023** |
| beta | rh-fusiform | 0.048 | 7 | 0.0078 | **0.027** |
| beta | rh-inferiortemporal | 0.0293 | 8 | 0.0039 | **0.023** |
| beta | rh-middletemporal | 0.0238 | 9 | 0.002 | **0.023** |
| beta | Right-Hippocampus | 7.23E-02 | 8 | 0.0039 | **0.023** |
| beta | Right-Amygdala | 0.0586 | 8 | 0.0039 | **0.023** |
| beta | lh-lateralorbitofrontal | 0.0216 | 6 | 0.016 | **0.034** |
| beta | lh-rostralmiddlefrontal | 0.0109 | 5 | 0.031 | **0.044** |
| beta | lh-inferiortemporal | 0.0209 | 7 | 0.0078 | **0.027** |
| beta | lh-middletemporal | 0.0127 | 7 | 0.0078 | **0.027** |
| beta | Left-Hippocampus | 0.0134 | 6 | 0.016 | **0.034** |
| #patients IED>no IED: number of patients for which the value in the ROI was higher in the scalp negative IED than the no IED condition. ROI names correspond to the ones of the Desikan atlas.  lh: left hemisphere, rh: right hemisphere, ROI: region of interest. | | | | | |
